# Mapping the distribution of Nipah virus infections: a geospatial modelling analysis

**DOI:** 10.1016/S2542-5196(24)00119-0

**Published:** 2024-07

**Authors:** Yan-Qun Sun, Yuan-Yuan Zhang, Mei-Chen Liu, Jin-Jin Chen, Ting-Ting Li, Yan-Ning Liu, Ling-Yu Zhang, Tao Wang, Lin-Jie Yu, Tian-Le Che, Tian Tang, Qiang Xu, Chen-Long Lv, Bao-Gui Jiang, Nick Golding, Max L. Mehlman, Simon I. Hay, Li-Qun Fang, Wei Liu

**Affiliations:** State Key Laboratory of Pathogen and Biosecurity, Beijing Institute of Microbiology and Epidemiology, Beijing, China; Nanjing Municipal Center for Disease Control and Prevention, Affiliated Nanjing Center for Disease Control and Prevention of Nanjing Medical University, Nanjing, China; State Key Laboratory of Pathogen and Biosecurity, Beijing Institute of Microbiology and Epidemiology, Beijing, China; State Key Laboratory of Pathogen and Biosecurity, Beijing Institute of Microbiology and Epidemiology, Beijing, China; The First Affiliated Hospital, Anhui Medical University, Hefei, China; State Key Laboratory of Pathogen and Biosecurity, Beijing Institute of Microbiology and Epidemiology, Beijing, China; State Key Laboratory of Pathogen and Biosecurity, Beijing Institute of Microbiology and Epidemiology, Beijing, China; School of Public Health, Guizhou Medical University, Guiyang, China; State Key Laboratory of Pathogen and Biosecurity, Beijing Institute of Microbiology and Epidemiology, Beijing, China; State Key Laboratory of Pathogen and Biosecurity, Beijing Institute of Microbiology and Epidemiology, Beijing, China; State Key Laboratory of Pathogen and Biosecurity, Beijing Institute of Microbiology and Epidemiology, Beijing, China; State Key Laboratory of Pathogen and Biosecurity, Beijing Institute of Microbiology and Epidemiology, Beijing, China; State Key Laboratory of Pathogen and Biosecurity, Beijing Institute of Microbiology and Epidemiology, Beijing, China; State Key Laboratory of Pathogen and Biosecurity, Beijing Institute of Microbiology and Epidemiology, Beijing, China; State Key Laboratory of Pathogen and Biosecurity, Beijing Institute of Microbiology and Epidemiology, Beijing, China; State Key Laboratory of Pathogen and Biosecurity, Beijing Institute of Microbiology and Epidemiology, Beijing, China; State Key Laboratory of Pathogen and Biosecurity, Beijing Institute of Microbiology and Epidemiology, Beijing, China; Telethon Kids Institute, Nedlands, Western Australia, Australia; School of Population Health, Curtin University, Bentley, Western Australia, Australia; Melbourne School of Population and Global Health, University of Melbourne, Parkville, Victoria, Australia; Department of Health Metrics Sciences, School of Medicine, University of Washington; Institute for Health Metrics and Evaluation, University of Washington; Department of Health Metrics Sciences, School of Medicine, University of Washington; Institute for Health Metrics and Evaluation, University of Washington; State Key Laboratory of Pathogen and Biosecurity, Beijing Institute of Microbiology and Epidemiology, Beijing, China; State Key Laboratory of Pathogen and Biosecurity, Beijing Institute of Microbiology and Epidemiology, Beijing, China; The First Affiliated Hospital, Anhui Medical University, Hefei, China

## Abstract

**Background:**

Nipah virus is a zoonotic paramyxovirus responsible for disease outbreaks with high fatality rates in south and southeast Asia. However, knowledge of the potential geographical extent and risk patterns of the virus is poor. We aimed to establish an integrated spatiotemporal and phylogenetic database of Nipah virus infections in humans and animals across south and southeast Asia.

**Methods:**

In this geospatial modelling analysis, we developed an integrated database containing information on the distribution of Nipah virus infections in humans and animals from 1998 to 2021. We conducted phylodynamic analysis to examine the evolution and migration pathways of the virus and meta-analyses to estimate the adjusted case-fatality rate. We used two boosted regression tree models to identify the potential ecological drivers of Nipah virus occurrences in spillover events and endemic areas, and mapped potential risk areas for Nipah virus endemicity.

**Findings:**

749 people and eight bat species across nine countries were documented as being infected with Nipah virus. On the basis of 66 complete genomes of the virus, we identified two clades—the Bangladesh clade and the Malaysia clade—with the time of the most recent common ancestor estimated to be 1863. Adjusted case-fatality rates varied widely between countries and were higher for the Bangladesh clade than for the Malaysia clade. Multivariable meta-regression analysis revealed significant relationships between case-fatality rate estimates and viral clade (p=0·0021), source country (p=0·016), proportion of male patients (p=0·036), and travel time to health-care facilities (p=0·036). Temperature-related bioclimate variables and the probability of occurrence of *Pteropus medius* were important contributors to both the spillover and the endemic infection models.

**Interpretation:**

The suitable niches for Nipah virus are more extensive than previously reported. Future surveillance efforts should focus on high-risk areas informed by updated projections. Specifically, intensifying zoonotic surveillance efforts, enhancing laboratory testing capacity, and implementing public health education in projected high-risk areas where no human cases have been reported to date will be crucial. Additionally, strengthening wildlife surveillance and investigating potential modes of transmission in regions with documented human cases is needed.

**Funding:**

The Key Research and Development Program of China

## Introduction

The genus *Henipavirus*, which belongs to the family Paramyxoviridae, contains several viruses that have been identified in bats, including Nipah virus,^1^ Hendra virus,^2^ Cedar virus,^3^ Ghana virus,^4^ and Angavokely virus.^5^ Nipah virus and Hendra virus emerged in the late 20th century and cause severe illness in humans.^1,2^ Nipah virus was first isolated from patients with encephalitis during a 1998–99 epidemic among pig farmers in Malaysia,^6^ during which pigs were identified as the source of human infections.^7^ Although most infected pigs had mild illness, severe cases were reported in humans, characterised by encephalitis or respiratory illness and resulting in considerable human mortality.^6^ Since then, other outbreaks have been reported in south Asia, including in western and northwestern Bangladesh,^8^ over the Bangladesh–India border in West Bengal, India, and in Kerala, India.^9,10^ Although Nipah virus is geographically limited to particular regions, it can infect a wide range of animals and cause severe disease with a case-fatality rate ranging from 43% to 100% in humans, making it a considerable public health concern.^11^ WHO has included Nipah virus on its list of top ten diseases requiring prioritised research and intervention in the Research and Development Blueprint.^12^

Epidemiological studies suggest that Nipah virus has multiple modes of transmission.^8,13–15^ Most infections are sporadic and zoonotic in nature.^16^ Cross-species transmission is primarily driven by reservoir bats,^16^ particularly through the consumption of food products that have been contaminated by bats,^17,18^ although other mammals can also serve as hosts.^13,19^ *Pteropus* bats, also known as flying foxes or fruit bats, serve as the main natural reservoirs for henipaviruses, and Nipah virus has been detected in these bats across the Indo-Pacific region.^16,20–22^ As one of the largest bat populations globally, *Pteropus bats* inhabit a vast range that spans south Asia and southeast Asia as well as Australia, east Africa, and numerous oceanic islands within the Indian and Pacific Oceans;^23^ as such, areas known to harbour these bats should remain highly vigilant regarding the potential threat of Nipah virus. However, owing to the scarcity of sensitive surveillance systems capable of detecting human infections, cases of Nipah virus are likely to be under-reported,^24^ and efforts to predict risk patterns and their associated uncertainties are urgently needed.

The epidemiological characteristics of Nipah virus in Bangladesh and India differ from those in Malaysia and Singapore, with notable variations observed in fatality rates and transmission patterns. In Bangladesh, Nipah virus infections have a high fatality rate and are rarely associated with pig-to-human transmission.^11^ The routes of infection, modes of transmission, pathogenicity, capabilities of the health-care systems (such as the ability to treat severe cases and the amount of care required), and follow-up outcomes vary among regions,^11^ for which the underlying reasons and clinical implications remain unclear. Although several reviews have been conducted, most have been at the country level and have often been inconsistent in terms of the areas and timelines covered.^25–28^

Through a literature review and multiple analyses of public data and official reports, we conducted an in-depth examination of the epidemiological characteristics of Nipah virus infection since it was first identified in 1999. Additionally, we explored the relationship between various environmental, socioeconomic, and biological factors and the risk of Nipah virus infection. By constructing an integrated model, we mapped the probability of Nipah virus infection in areas where few epidemiological investigations had previously been conducted.

## Methods

### Database on Nipah virus

In this geospatial modelling analysis, we established a multisource database of Nipah virus infections. This database comprises information obtained through systematic searches of English-language databases: Web of Science, PubMed, Indian Journals, MyJurnal (Citation and Infometric Division, Malaysia), the Thai Journal Citation Index, and Bangladesh Journals Online (appendix p 18). Additionally, our database included information from grey literature from government, academic, or professional organisations: WHO, the National Centre for Disease Control of India, the Institute of Epidemiology, Disease Control and Research (IEDCR) of Bangladesh, and the International Centre for Diarrhoeal Disease Research, spanning Jan 1, 1999 to Nov 19, 2022 (appendix pp 19–21). The search strategy was designed to identify relevant sources reporting on Nipah virus infection in humans or animals using appropriate methods. The following search terms were used: “Nipah virus”, “Nipah”, “NiV”, “*Henipavirus*”, or “Hendra like virus”. Two authors (Y-YZ and M-CL) independently screened the titles and abstracts of all retrieved studies for inclusion. Two additional independent reviewers (J-JC and T-TL) retrieved the full text of all studies identified as potentially eligible and further assessed their eligibility according to predetermined criteria. We excluded editorials or opinion articles and reviews, studies focused on the structure of the virus or its effects at the molecular or cellular level, experimental animal studies investigating Nipah virus transmission, drug or vaccine trials without geographical or clinical information pertaining to cases, qualitative studies or economic assessments related to Nipah virus infection, and duplicated data associated with identical infection events; detailed inclusion and exclusion criteria are listed in the appendix (p 22). The criteria for human infections were defined on the basis of guidelines from WHO, the US Centers for Disease Control and Prevention (CDC), and the IEDCR;^29–31^ that is, cases were considered as human infections if they had one or more of the following laboratory test results: a positive Nipah virus culture, a positive detection of Nipah virus RNA through molecular testing, the presence of IgM antibodies against Nipah virus, seroconversion, or a greater than four-fold increase in Nipah virus antibody response between acute and convalescent serum samples.^29,31^ The criteria for animal infections were based on guidelines by the UN Food and Agriculture Organization and the World Organisation for Animal Health (appendix p 23).^32^ Serological results from healthy individuals or from animals were excluded owing to the potential of cross-reactivity with other henipaviruses or related paramyxoviruses; for example, positive serological tests for antibodies against Henipa-like viruses found in bats in Brazil,^33^ Madagascar,^34^ Ghana,^35^ and Uganda^36^ are not sufficient indicators of the risk of zoonotic Nipah virus transmission.

To extract data from selected literature sources, we used a standard form (appendix p 24). Transmission routes for human infections were classified as zoonotic transmission (direct contact with infected animals or consumption of food products contaminated by body fluids of infected animals) or person-to-person transmission (close contact with an individual infected with Nipah virus or their body fluids) according to US CDC criteria.^37^ Zoonotic transmission was further defined as either direct exposure to Nipah virus hosts or indirect contact with objects contaminated by bats.^38^

### Phylogenetic and phylogeographic analysis

To conduct a phylogenetic and phylogeographic analysis of Nipah virus, we retrieved genome records from GenBank. For this phylodynamic analysis, we included only genomes that had at least 90% of the full length (18246 bp) sequenced and fewer than 110 degenerate bases. The evolutionary analysis of Nipah virus was conducted using BEAST 2 version 2.6.3.^39^ Bayesian stochastic search variable selection was used to model the evolution, with ModelFinder used to identify the best-fitting model (GTR+F+G4) for maximum likelihood analyses.^40^ The temporal structure of Nipah virus complete genomes was explored using TempEst version 1.5.3.^41^ For Bayesian phylogeny construction we used a general time-reversible substitution model with gamma distribution and invariant sites (GTR+F+G4), an uncorrelated log-normal relaxed molecular clock model, and a Bayesian skyline prior. Convergence of Markov chain Monte Carlo was assessed, and the maximum clade credibility tree was identified after discarding the initial 10% burn-in using Tracer version 1.7.1.^42^ The resulting tree was visualized using FigTree version 1.4.4, and phylogeographic transmission routes were visualised using SpreaD3 version 0.9.7.^43^ Details of the analysis process are given in appendix(p 5).

### Meta-analyses of case-fatality rate and clinical manifestations

The meta-analyses were conducted using the R package meta to estimate the case-fatality rate, the frequency of clinical manifestations in human infections, and the positive detection rate of Nipah virus in reservoir hosts.^44^ In brief, we used the Freeman-Tukey double arcsine transformation to estimate the weight of each study, from which the pooled estimates (case-fatality rate, frequency of clinical manifestations, and positive detection rate) and their 95% CIs were calculated. Heterogeneity was assessed using the Higgin’s *I*^2^ statistic; if its value exceeded 50% we used the DerSimonian-Laird random-effects model, otherwise we applied the fixed-effects model. Publication bias was evaluated through funnel plots of outcomes. We did subgroup meta-analyses in addition to univariable and multivariable meta-regression analyses on case-fatality rates, to explore possible sources of heterogeneity and examine the effects of the study characteristics (country, year of publication, mean age of patients, proportion of male patients, viral clade, and risk of bias) and four socioeconomic indicators (travel time to health-care facilities, gross domestic product [GDP] per capita, human development index, and Gini index) reflecting access to health care, poverty levels, social vulnerability, and income inequality at the province level (appendix pp 7–8).

### Georeferencing and modelling of date palms, Nipah virus reservoir hosts, and Nipah virus infections

We collected data on 50 variables that were thought to be potentially associated with the occurrence of Nipah virus: 31 environment-related, nine human activity-related, and ten animal-related (data sources, resolutions, and rescaling are described in the appendix, pp 28–31). These variables were included because of their relevance to the ecology and transmission dynamics of Nipah virus or to the distribution and abundance of main Nipah virus reservoir hosts, as indicated by previous studies (appendix pp 32–41), and we aimed to explore their potential association with the occurrence of Nipah virus or its primary reservoir hosts while assessing their contribution in predicting the ecological niche of this virus.

We included study areas in south Asia, southeast Asia, east Asia, and Oceania, encompassing countries or regions with documented historical records of Nipah virus infection or relevant bat species (appendix p 9). Because date palm sap is suggested to be an important pathway for Nipah virus transmission,^45,46^ we first compiled data on the occurrence data of date palms (*Phoenix sylvestris*) from reputable databases: the Global Biodiversity Information Facility (GBIF), Integrated Digitized Biocollections (iDigBio), and iNaturalist (all last updated on Dec 10, 2023). Data on the occurrence of bat species associated with Nipah virus infections were obtained from several databases: DarkCideS 1.0, Bat Eco-Interactions, GBIF, iDigBio, and iNaturalist. The bat data were further supplemented by information resulting from searching PubMed and the China National Knowledge Infrastructure, from Nov 21, 2003, to Nov 21, 2022, using the species names as search terms; publications retrieved are described in the appendix (pp 134–38). To ensure taxonomic accuracy and range consistency of the bat data, they were cross-referenced with shapefile coverage from the International Union for Conservation of Nature (IUCN) Red List of Threatened Species (last updated Dec 10, 2022). Details of the data processing are provided in the appendix (pp 9–13).

The boosted regression tree approach used the R package dismo to generate the ecological niche model for date palms and seven main Nipah virus reservoir hosts: *Pteropus lylei*, *Pteropus medius* (formerly *Pteropus giganteus*), *Pteropus vampyrus*, *Pteropus hypomelanus*, *Rousettus leschenaultii*, *Rousettus amplexicaudatus*, and *Hipposideros larvatus*.^47^ The occurrences of Nipah virus infection were predicted by applying two distinct models: a spillover model that specifically identifies areas conducive to Nipah virus spillover by focusing on the human index case or confirmed spillover event for each outbreak, while excluding any outbreaks or human cases involving only human-to-human transmission or transmission from intermediary hosts to humans; and an endemic infection model that evaluates areas where Nipah virus is present in humans, intermediary hosts, and reservoir hosts while excluding outbreak events or cases transmitted only between humans or between intermediary hosts. This clear separation ensures a precise delineation between spillover risk and the broader risk of endemic infection.

The boosted regression tree models effectively address correlations between variables while mitigating overfitting and improving model performance through cross-validation-based selection of the number of trees, shrinkage, bagging, and relative contribution techniques.^48^ The use of these techniques improve the robustness and regularisation of the model and its ability to generalise when dealing with complex datasets containing correlated predictors. For our primary analysis, we used a tree complexity of 5, cross-validation folds of 10, a learning rate of 0·005, and a bagging fraction of 75% on the basis of their satisfactory performance in our previous research.^49–51^ The training set consisted of 80% of the data points, randomly sampled without replacement, whereas the remaining 20% of the data points served as the test set. To select the optimal number of trees in the boosted regression tree models, we did the cross-validation step and used gbm.perf for model regularisation. To enhance the robustness of the model’s predictions and to quantify the associated uncertainties, we fitted an ensemble of 100 boosted regression tree submodels. To further assess model robustness, 80% of the presence data were randomly subsampled and the top 20 predictor variables were selected on the basis of their contribution to model performance and ecological relevance. The ecological niche models using the boosted regression tree approach were then retrained as a sensitivity analysis for both date palms and Nipah virus reservoir hosts, as well as for Nipah virus infection. Absolute out-of-sample performance metrics were then calculated. The detailed modelling processes are described in the appendix (pp 9–13).

### Role of the funding source

The funder of the study had no role in study design, data collection, data analysis, data interpretation, or writing of the report.

## Results

**Figure 1:**
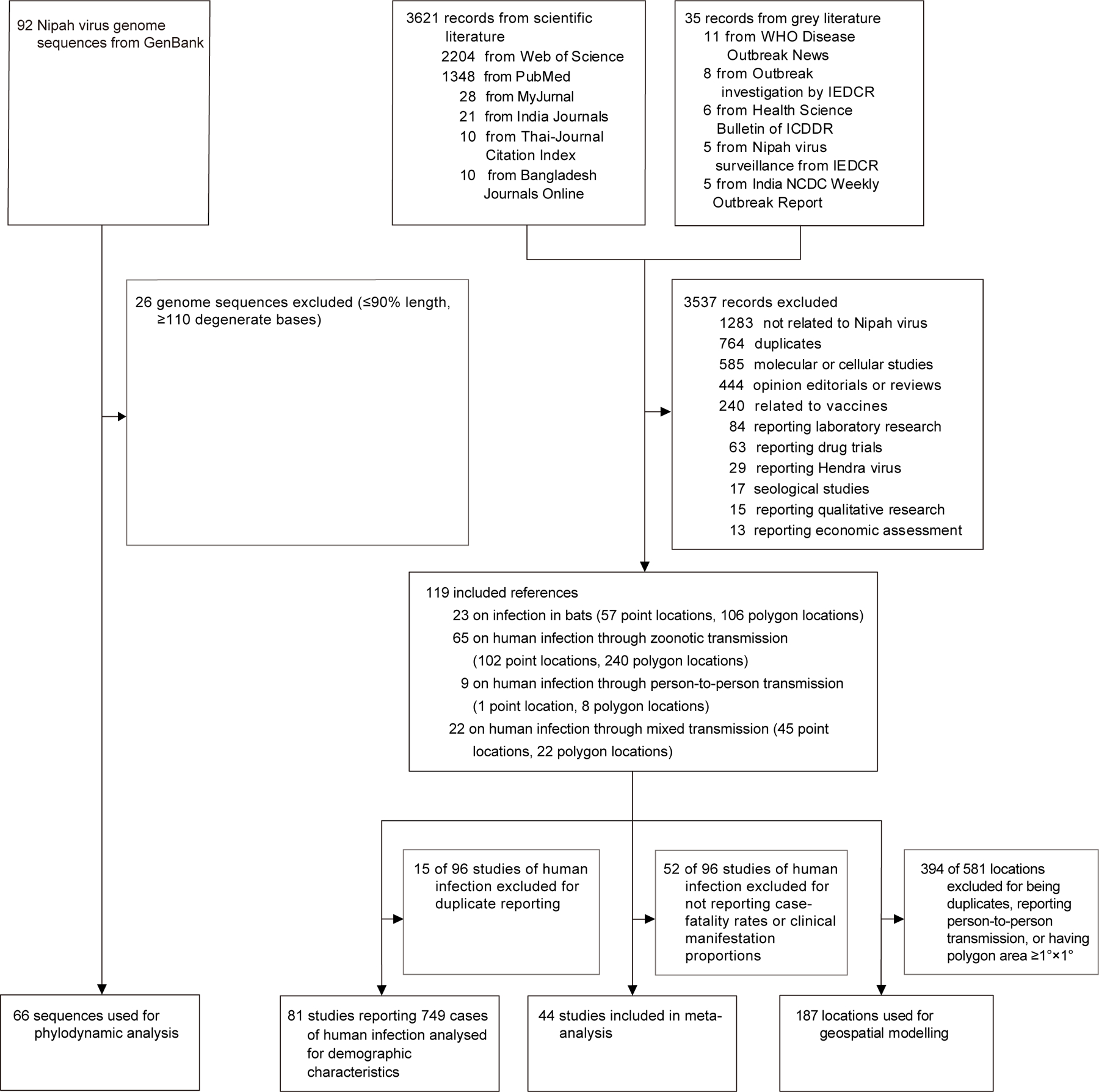
Flow chart of the literature review and study design ICDDR=International Centre for Diarrhoeal Disease Research. IEDCR=Institute of Epidemiology, Disease Control and Research. NCDC=National Centre for Disease Control.

We reviewed 3621 records from scientific literature and 35 records from grey literature published between Jan 1, 1999, and Nov 19, 2022. Of these 3656 records, 119 (97 from scientific literature and 22 from grey literature) met the criteria and were included in the data extraction process (appendix pp 125–30). The pooled data yielded an integrated database comprising bat infections (23 sources recording 57 point locations and 106 polygon locations), human infections through zoonotic transmission (65 sources recording 102 point locations and 240 polygon locations), human infections through person-to-person transmission (nine sources recording one point location and eight polygon locations), and human infections resulting from outbreak events involving both zoonotic and person-to-person transmission (22 sources recording 45 point locations and 22 polygon locations; figure 1).

749 human cases of Nipah virus infection were reported in five countries (Bangladesh, India, Malaysia, Singapore, and the Philippines; table 1). Among those with known demographic information, adults aged 15–59 years accounted for 89% (358 of 402) of cases, men constituted 74% (391 of 530) of cases, and livestock practitioner was the most frequently recorded occupation (238 [68%] of 351 cases). Among the 489 patients with known exposure history, 336 (69%) reported animal contact, 127 (26%) reported exposure to patients with the disease, and 26 (5%) reported exposure to palm (eating date palm fruits, drinking date palm sap, or climbing date palm trees; table 1). Three epidemiological characteristics of patients – sex, exposure pattern, and temporal pattern of the disease – varied among five reporting countries (appendix p 14).

Nipah virus infections were documented in 425 bats across seven countries: Thailand, Cambodia, Malaysia, Indonesia, and Timor-Leste (all in southeast Asia) and Bangladesh and India (both in south Asia; appendix p 65). Eight bat species belonging to four genera within three families were identified as naturally infected with Nipah virus. These species were *R amplexicaudatus* with a positive rate of 8% (95% CI 2–16) computed by subgroup meta-analyses, followed by *Pipistrellus pipistrellus* (3%; 0–12), *R leschenaultii* (3%; 0–10), *P lylei* (1%; 1–2), *P hypomelanus* (1%; 0–4), *P medius* (<1%; 0–1), *P vampyrus* (<1%; 0–0), and *H larvatus* (not included in meta-analyses; appendix pp 43, 65, 92).

**Figure 2:**
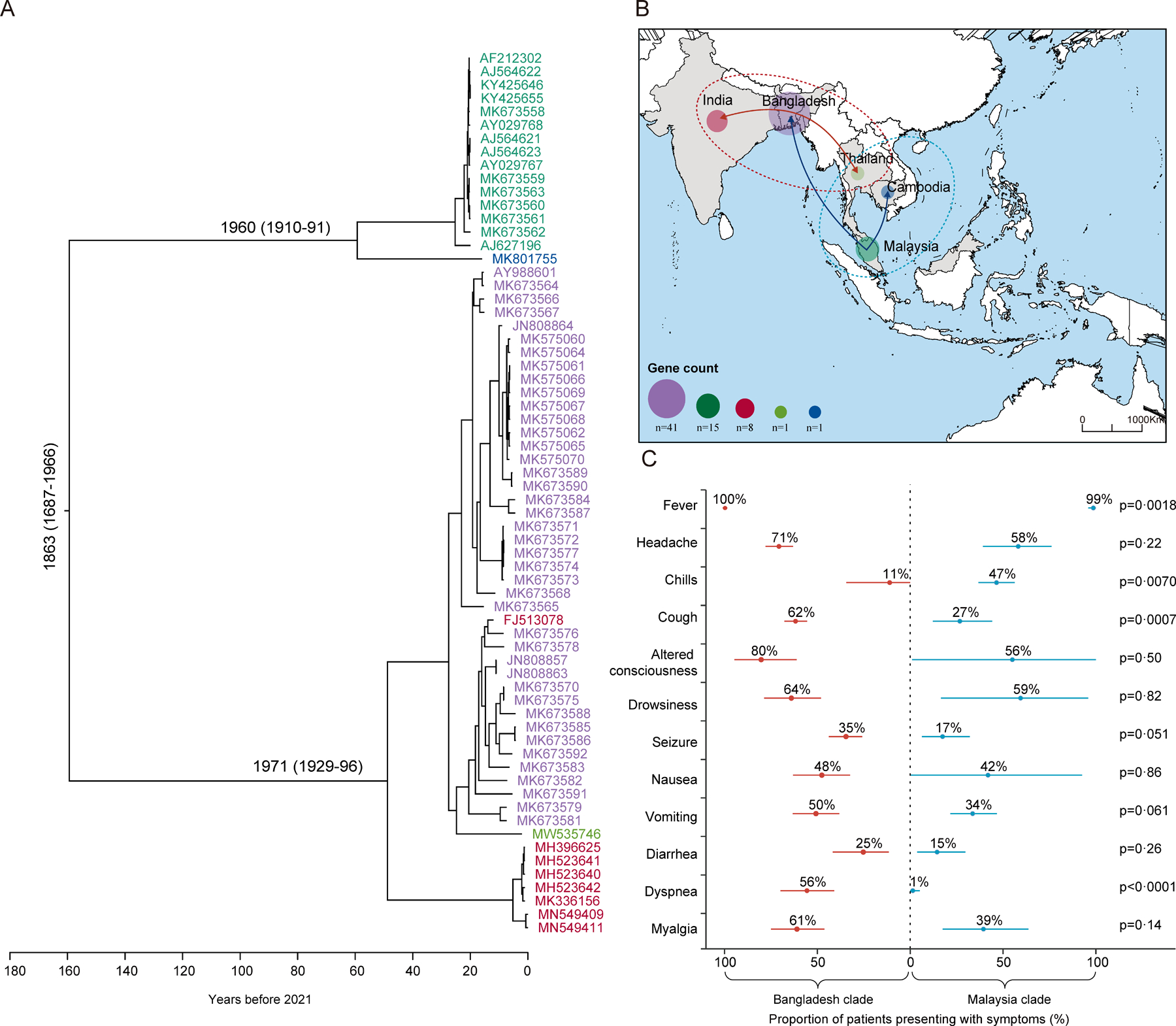
Evolution of Nipah virus and the clinical symptoms of Nipah virus infection (A) Maximum likelihood tree of Nipah virus based on 66 complete Nipah virus genome sequences from 1999 to 2021. (B) Map illustrating the spatial distribution of the Bangladesh clade (circles surrounded by red dotted lines) and the Malaysia clade (circles surrounded by blue dotted lines) across south and southeast Asia. The migration route of Nipah virus evolution based on evolutionary analysis using 66 complete Nipah virus genome sequences is shown by arrows. (C) Proportion of patients presenting with various clinical manifestations by viral clade. Error bars are 95% CI.

We used 66 complete Nipah virus genome sequences for our analysis (43 from human patients, 14 from bats, eight from pigs, and one from a dog; dated from 1999 to 2021 and obtained from GenBank; appendix pp 25–27). Five countries contributed genome sequence data, with the majority coming from Bangladesh (41 of 66; 62%), followed by Malaysia (15; 23%), India (eight; 12%), Thailand (one; 2%), and Cambodia (one; 2%). Phylogenetic analysis of the complete genome sequences revealed that the circulating Nipah viruses could be clustered into two clades: a Malaysia clade (consisting of strains from Malaysia, Thailand, and Cambodia) and a Bangladesh clade (encompassing strains from Bangladesh, India, and Thailand; figure 2A). Through phylogeographic analysis we estimated that the most recent common ancestor of Nipah virus occurred in approximately 1853 (95% CI 1687–1966), with the emergence of the Malaysia clade around 1960 (95% CI 1910–91) and the Bangladesh clade around 1971 (1929–96; figure 2A). Spatiotemporal transmission patterns indicated that, initially, the Malaysia clade first spread from Malaysia to Bangladesh and then to Cambodia, whereas the Bangladesh clade first spread from Bangladesh to India and subsequently to Thailand (figure 2B). The Nipah virus sequences from India were further clustered into two subclades within the Bangladesh clade on the basis of their phylogenetic relationship with other sequences. One subclade comprised sequences detected in Bangladesh and a single sequence (GenBank FJ513078) from West Bengal, India, whereas all other sequences from India formed another subclade within this group.

We included 22 studies in the meta-analysis on case-fatality rate. The overall case-fatality rate was estimated to be 71%, and the adjusted values differed by country: 78% (95% CI 53–96) in India, 77% (68–84) in Bangladesh, 53% (29–76) in the Philippines, 40% (25–55) in Malaysia, and 9% (0–35) in Singapore. The adjusted case-fatality rate of the Bangladesh clade (77%; 69–84) was substantially higher than that of the Malaysia clade (37%; 24–52). Univariable meta-regression analysis revealed significant associations between the heterogeneity of case-fatality rate estimates and Nipah virus clade (p<0·0001), source country (p=0·0026), mean age of patients (p=0·011), GDP per capita (p<0·0001), human development index (p=0·0078), and travel time to health-care facilities (p=0·036; appendix pp 46–48, 81). In multivariable analyses, four variables remained significant: viral clade (p=0·0021), source country (p=0·016), proportion of male patients (p=0·036), and travel time to health-care facilities (p=0·036). Permutation tests indicated the robustness (p=0·036) of our multivariable regression model. Results of the case-fatality rate meta-analysis are provided in the appendix (pp 68–77).

We included 31 studies in the meta-analysis of clinical manifestations. Fever (proportion 100; 95% CI 100–100), altered consciousness (77%; 58–92), and headache (67%; 60–75) were the most frequent among 11 clinical symptoms recorded. Subgroup meta-analyses revealed a significant difference in four symptoms between infections with the Bangladesh clade and the Malaysia clade: fever (100% vs 99%, p=0·0018), cough (62% vs 27%, p=0·0007), dyspnoea (56% vs 1%, p<0·0001), and chills (11% vs 47%, p=0·0070; figure 2C, appendix pp 82–91).

**Figure 3:**
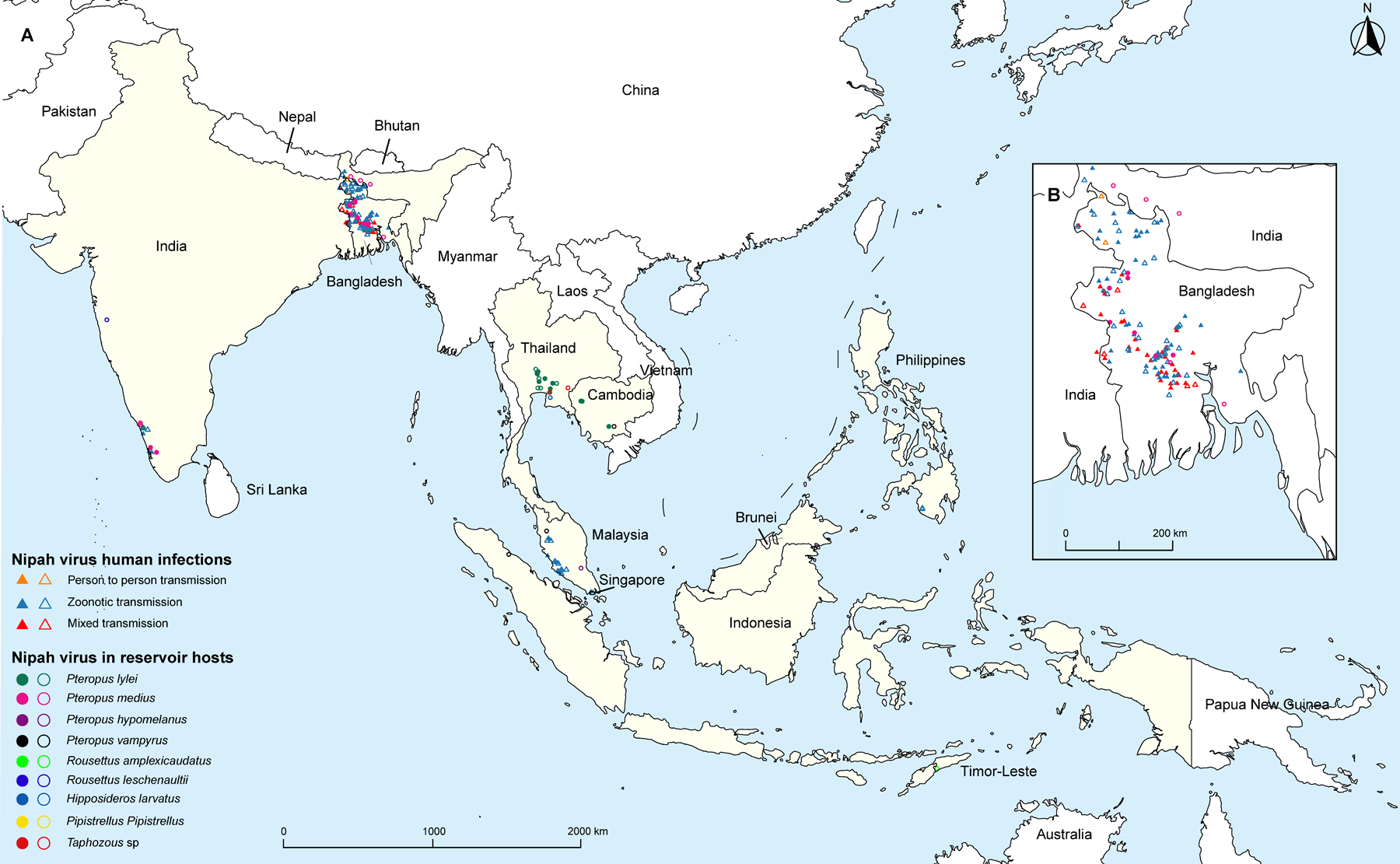
Distribution of Nipah virus infections in humans and Nipah virus detection in reservoir hosts. (A) Occurrences of Nipah virus in the study area. (B) A magnified map displaying the occurrences in Bangladesh. The cases of human infection are categorised by transmission mechanism (triangles) and cases in reservoir hosts are classified by species (circles). The area coloured in beige indicates the countries that are affected by Nipah virus: India, Bangladesh, Thailand, Cambodia, Malaysia, Singapore, the Philippines, Indonesia, and Timor-Leste. Mixed transmission refers to the coexistence of both zoonotic and person-to-person transmission. The occurrence of the intermediate host is consistent with the occurrence of some human zoonotic infections (appendix pp 51–55).

We identified 195 occurrences of Nipah virus infections in humans, intermediary hosts, and reservoir hosts across nine countries (figure 3A). These occurrences consisted of 115 point locations and 80 polygon locations, mostly observed in Bangladesh (130 occurrences; figure 3B), followed by Malaysia (22 occurrences), India (19 occurrences), and Thailand (16 occurrences). After excluding person-to-person transmission, we retained 187 occurrences to simulate zoonotic transmission of Nipah virus using both a spillover model (124 locations) and an endemic infection model involving humans, intermediary hosts, and reservoir hosts (all 187 locations; appendix pp 51–55). The predicted niche for date palms (*P sylvestris*) is located mainly in the south Asian subcontinent (appendix p 107). Predicted niches for seven Nipah virus reservoir hosts align closely with their spatial ranges identified from the IUCN data (appendix p 108).

**Figure 4:**
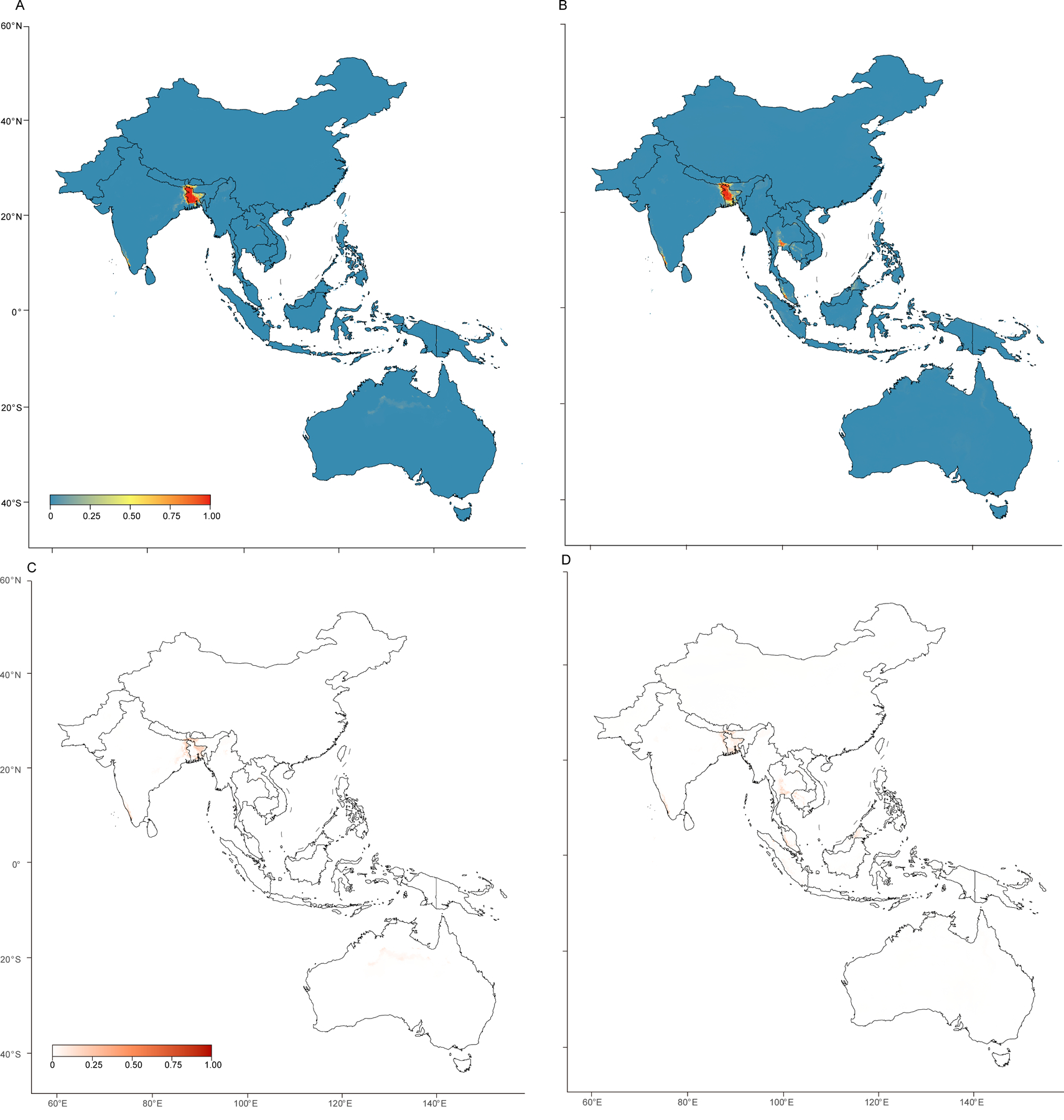
Predicted environmental suitability for spillover events and Nipah virus endemicity (A) Environmental suitability for spillover events, as predicted by the spillover model, based on occurrences of Nipah virus infections in humans. (B) Environmental suitability for Nipah virus endemicity, as predicted by the endemic infection model, based on occurrences of Nipah virus infections in humans, intermediary hosts, and reservoir hosts. Predicted suitability values in (A) and (B) are scaled from 0·00 to 1·00. (C) Map illustrating the uncertainty associated with the spillover model. (D) Map illustrating the uncertainty associated with the endemic infection model. Predicted uncertainty values in (C) and (D) are scaled from 0·00 to 1·00.

Areas predicted by the spillover model to be at potential risk of spillover events (figure 4A) were highly consistent with those forecast as endemic areas by the endemic infection model (figure 4B), although with slight geo-graphical disparities as no spillover events were predicted in Thailand or Cambodia. Uncertainty distributions for both models are shown in figure 4C, D. The accuracy of these two ecological models is shown by the average area under the receiver operating characteristic curves reaching 99·9% for the spillover model and 97·7% for the endemic infection model (appendix p 120). The estimated drivers varied between the models. Temperature-related bioclimate variables made important contributions to both the spillover and the endemic infection models. Specifically, the mean temperature of the driest quarter, the annual mean temperature, and the mean temperature of the coldest quarter were the top three driving factors in the spillover model, whereas the mean temperature of the driest quarter and the mean temperature of the warmest quarter were the first and third largest factors in the endemic infection model. The probability of occurrence of *P medius* was also an important contributor to both models (ranked fourth in the spillover model and second in the endemic infection model). Details of the drivers and their relative contributions can be found in the appendix (pp 59–60).

By overlaying population information onto the Nipah virus-suitable areas (calculated on the basis of the cutoff value of the model), we assessed both the at-risk population size and the geographical range of potential Nipah virus endemic areas (appendix p 121). The regions identified as potential risk areas for Nipah virus endemicity spanned 185 312 km^2^ (table 2). The country with the largest predicted areas for potential Nipah virus endemicity was Bangladesh (104 947 km^2^), followed by India (40 856 km^2^), Thailand (20 075 km^2^), Malaysia (11 886 km^2^), and Cambodia (2728 km^2^; table 2). In total, approximately 176·2 million people reside within predicted endemic areas, with Bangladesh having the largest at-risk population (106·7 million), followed by India (36·4 million), Thailand (16·5 million), Malaysia (10·2 million), and Indonesia (2·6 million; table 2). These risk areas are predominantly located in densely populated regions. Overall, the regions predicted by our models to have medium to high potential risk of Nipah virus endemicity are broader than those that reported Nipah virus presence in humans (excluding human-to-human transmission) and reservoir hosts, and appear to be well within estimates from previous studies, some of which indicate that much of south and southeast Asia is potentially at risk based only on the occurrence of *Pteropus* bats.^53–55^

## Discussion

Growing interest in Nipah virus over the past 20 years has resulted in increased reporting of new infections and the submission of viral sequences from an expanding geographical range and a growing number of bat species. The recent accumulation of data presents an opportunity to use a robust framework to assess the disease over a wide area. We conducted an extensive investigation into Nipah virus, encompassing epidemiology, phylogenetics, clinical medicine, host ecology, and spatial distribution.

The descriptive characteristics in our study offer insight into building a comprehensive Nipah virus dataset, particularly regarding the variability of patient follow-up across different regions and over time. This variability underscores the importance of implementing standardised data collection practices, specifically tailored to Nipah virus surveillance and research, to mitigate such biases and ensure the reliability and comparability of findings across different regions and time periods affected by Nipah virus outbreaks. The necessity of standardised data collection practices remains crucial in ecological niche modelling analysis within epidemiological research. We advocate for persistent efforts to develop and implement standardised data collection tools and guidelines, aiming to facilitate more rigorous and consistent data collection practices in future research endeavours.

The estimates of time to most recent common ancestor suggest that Nipah virus could have been circulating for more than 100 years, diverging over time and evolving independently into two distinct clades. The two clades have different epidemiological and ecological histories. We observed substantial variation between clades in the case-fatality rate, influenced by multiple factors. The case-fatality rate of the Bangladesh clade is higher than that of the Malaysia clade, which could be partially attributed to the more severe clinical symptoms observed in patients infected with the Bangladesh clade. However, animal studies comparing virulence between these two clades did not consistently recapitulate clinical phenotypes. An experimental animal study using African green monkeys showed more severe clinical symptoms and a higher case-fatality rate associated with infection with the Bangladesh clade;^56^ however, studies in ferrets^57^ and hamsters^58^ showed an opposite pattern. Although the pathogenicity of various strains has a crucial role, other factors, such as socioeconomic inequality in affected areas, also contribute substantially. The multivariable analysis presented here reveals an association between heterogeneity in case-fatality rate estimates and source country as well as travel time to health-care facilities, indicating adverse effects and significantly worse outcomes among lower-income regions and those with fragile social and public health infrastructure. Sex and gender could have an important role in the epidemiology and clinical outcomes of Nipah virus infection. Biological factors, such as differences in immune responses or hormonal influences^59^—reflecting complex interactions among hormones, genes, and the environment— could affect the course of Nipah virus infection. Further research is needed to elucidate the specific mechanisms by which sex and gender influence Nipah virus infection and outcomes.

Despite various hypotheses on the mechanisms of bat-to-livestock and bat-to-human transmission, the scarcity of data hinders the development of integrative models for virus transmission or effective strategies for disease control. Our boosted regression tree models highlight the pivotal roles of the primary reservoir bat species and bioclimatic factors in Nipah virus transmission. With expanding human populations encroaching upon natural habitats, closer proximity to bats increases the likelihood of direct contact between humans and these virus carriers, while also posing a risk of infecting domestic animals through exposure to bat droppings or urine.^60^ Bioclimatic variables have a substantial effect on Nipah virus transmission by influencing bat behaviour and migration patterns, thereby indirectly contributing to variations in both the timing and the extent of Nipah virus transmission.^61^

Phylogenetic analysis and ecological niche studies have revealed host diversity in the genetic evolution of Nipah virus, geographical overlaps among host ecological niches, and a wide area at risk of Nipah virus endemicity. Gregarious bats with similar ecological niches are more prone to sharing viruses than those that are more solitary, whereas regionally migrating bats have an important role in spreading viruses through networks due to their high degree of geographical overlap and taxonomic similarities, which suggest closer contact and greater opportunities for interaction.^62^ These factors contribute to natural advantages in viral connectivity or horizontal transmission within the same genus, promoting virus sharing and dissemination among these hosts. Collectively, these insights enhance our understanding of the complexity of Nipah virus transmission dynamics while informing prevention and control strategies.

In our analysis, we excluded serological results from healthy individuals and from animals, owing to the potential for cross-reactivity with other henipaviruses or related paramyxoviruses. These serological data can be applied in a broader model in further research to assess the distribution of all known members of the genus *Henipavirus*. Moreover, the dynamic measurement of animal antibodies can provide additional data on the duration of the antibody response. This information can serve as an alternative indicator within a broader probabilistic modelling framework, offering valuable insights into the spatial and temporal dynamics of *Henipavirus* transmission and facilitating more accurate predictions of the distribution of henipaviruses and their prevalence in reservoir hosts in high-risk areas projected by our ecological niche models. In addition, comparing uncertainty results derived from different data sources can facilitate the identification of the origins of uncertainty,^63^ thereby assisting in pinpointing weaknesses in Nipah virus surveillance within reservoir hosts and enhancing our predictive capabilities. We cautiously extrapolate the results of our model in areas where Nipah virus occurs or *Pteropus* bats inhabit, but we avoid global extrapolation. When operating outside the range of the training data during extrapolation, the model encounters new environmental combinations for which it has little or no reference. Furthermore, during extrapolation, the model assumes that those same functions continue to hold true even if the underlying ecological processes might be different outside of the observed range. This approach can lead to inaccurate predictions and increased uncertainty, especially if the true relationships exhibit sharp changes or non-monotonic trends beyond the training data. Although the complexity of the model might offer more flexibility to fit diverse scenarios, it can also render the model more prone to making unrealistic predictions owing to the lack of data to constrain the model behaviour. Therefore, a deeper understanding of precisely where extrapolation occurs is crucial for us and other researchers to effectively propagate uncertainty estimates. This iterative process acknowledges that extrapolation is not an endpoint but rather a step within a cycle of applying and refining our knowledge about Nipah virus. Our study has several limitations. First, the infection of humans with Nipah virus and subsequent development of disease is influenced by the nature of the initial exposure to the pathogen and its associated transmission probability. Our study aimed to understand the risk of disease emergence at the population level within a complex ecological and epidemiological context. Individual-level factors contributing to human infection, such as con-sumption of date palm sap and exposure to infected individuals or animals with Nipah virus infection, are rarely reported in public data and were not considered when predicting the population-level risk in our study.^8,46,64^ Second, it is highly likely that Nipah virus cases in humans are under-reported owing to limited surveillance efforts and inadequate diagnostic methods. Current approaches for disease surveillance could fail to identify mild or asymptomatic Nipah virus infections in affected countries, particularly in resource-poor regions.^65^ Also, our findings might still be influenced by potential imbalances in data or different biases for modelling analysis due to different levels of surveillance across regions. These factors could undermine the predictive accuracy of the model through misclassification of cases and controls.

Despite these limitations, our study maps potential niches suitable for the zoonotic transmission of Nipah virus and represents a robust assessment path for identifying regions at high risk of disease spillover. This information is valuable to health authorities, which can use it to prioritise public health interventions and allocate resources more effectively. Within the predicted high-risk areas—particularly Thailand and Cambodia, where no human cases have been reported to date—we recommend intensifying zoonotic surveillance efforts, enhancing laboratory testing capacity, and conducting public education campaigns. Furthermore, in India and Bangladesh, strengthening of wildlife surveillance and monitoring of potential modes of transmission will be crucial to effectively mitigate the risk of Nipah virus transmission. Additionally, further research should investigate the role of diverse animal populations in disease transmission and analyse extensive libraries of viral genomes collected at different times and locations to better understand the risk of Nipah virus infection in humans.

## Supplementary Material

Supplementary appendix

## Figures and Tables

**Table 1:** Epidemiological features of Nipah virus infections in humans, 1998–2021

	Overall (n=749)	Malaysia (n=283)	Singapore (n=11)	Bangladesh (n=322)	India (n=116)	Philippines (n=17)
Number of deaths	415	109	1	226	70	9
Crude case-fatality rate, %	55%	39%	9%	70%	60%	53%
Country-adjusted case-fatality rate, %	71%	40%	9%	77%	78%	53%
Case type						
Confirmed case	451/573 (79%)	275/275 (100%)	11 (100%)	73/154 (47%)	89 (77%)	3 (18%)
Probable case	122/573 (21%)	0	0	81/154 (53%)	27 (23%)	14 (82%)
Laboratory methods						
Serological methods	285/407 (70%)	191/275 (69%)	10 (91%)	75/84 (89%)	7/34 (21%)	2/3 (67%)
PCR	38/407 (9%)	0	1 (9%)	9/84 (11%)	27/34 (79%)	1/3 (33%)
Viral culture	84/407 (21%)	84/275 (30·6%)	0	0	0	0
Sex						
Male	391/530 (74%)	231 (82%)	11 (100%)	112/189 (59%)	21/30 (70%)	16 (94%)
Female	139/530 (26%)	52 (18%)	0	77/189 (41%)	9/30 (30%)	1 (6%)
Age group						
0–14 years	24/402 (6%)	12 (4%)	0	11/58 (19%)	1/33 (3%)	0
15–59 years	358/402 (89%)	259 (92%)	11 (100%)	44/58 (76%)	28/33 (85%)	16 (94%)
≥60 years	20/402 (5%)	12 (4%)	0	3/58 (5%)	4/33 (12%)	1 (6%)
Occupation						
Medical staff	10/351 (3%)	0	0	1/48 (2%)	7/15 (47%)	2/9 (22%)
Livestock practitioner	238/351 (68%)	219/268 (82%)	11 (100)	0	1/15 (7%)	7/9 (78%)
Farmer	42/351 (12%)	0	0	38/48 (79%)	4/15 (27%)	0
Soldier or policeman	7/351 (2%)	6/268 (2%)	0	1/48 (2%)	0	0
Student	12/351 (3%)	9/268 (3%)	0	2/48 (4%)	1/15 (7%)	0
Driver	23/351 (7%)	19/268 (7%)	0	3/48 (6%)	1/15 (7%)	0
Houseworker	17/351 (5%)	15/268 (6%)	0	1/48 (2%)	1/15 (7%)	0
Businessperson	2/351 (1%)	0	0	2/48 (4%)	0	0
Contact history						
Animals	336/489 (69%)	265/265 (100%)	11 (100%)	49/152 (32%)	1/46 (2%)	10/15 (67%)
Pigs	276/489 (56%)	265/265 (100%)	11 (100%)	0	0	0
Bats	5/489 (1%)	0	0	4/152 (3%)	1/46 (2%)	0
Horses	10/489 (2%)	0	0	0	0	10/15 (67%)
Cats	7/489 (1%)	0	0	7/152 (5%)	0	0
Dogs	14/489 (3%)	0	0	14/152 (9%)	0	0
Cows	14/489 (3%)	0	0	14/152 (9%)	0	0
Goats	2/489 (<1%)	0	0	2/152 (1%)	0	0
Chickens	5/489 (1%)	0	0	5/152 (3%)	0	0
Ducks	3/489 (1%)	0	0	3/152 (2%)	0	0
Patients	127/489 (26%)	0	0	78/152 (51%)	44/46 (96%)	5/15 (33%)
Palm-associated	26/489 (5%)	0	0	25/152 (16%)	1/46 (2%)	0
Exposure site						
Community	433/477 (91%)	283 (100%)	11 (100%)	114/118 (97%)	10/48 (21%)	15/17 (88%)
Hospital	44/477 (9%)	0	0	4/118 (3%)	38/48 (79%)	2/17 (12%)

Data are n, %, or n (%). Percentages might not total 100 owing to rounding.

**Table 2:** Predicted areas and populations at risk of potential Nipah virus endemic

	Area at risk (km^2^)	Population at risk (millions)
**Asia**		
South Asia		
Bangladesh*†	104947 (95386–116424)	106·7 (93·5–119·9)
India*†	40856 (34467–48496)	36·4 (31·6–44·5)
Nepal	2032 (1648–2705)	1·3 (0·8–2·8)
Sri Lanka	161 (50–423)	0·2 (0·0–0·6)
Southeast Asia		
Thailand†	20075 (15 705–25 333)	16·5 (8·7–21·5)
Malaysia*†	11886 (7328–20541)	10·2 (9·2–11·0)
Indonesia†	958 (100–1794)	2·6 (0·0–3·8)
Singapore*	153 (94–288)	1·1 (0·8–1·7)
Cambodia†	2728 (400–5812)	0·8 (0·2–1·4)
Viet Nam	184 (100–362)	0·1 (0·0–0·5)
Myanmar	432 (99–1217)	0·0 (0·0–0·1)
Philippines*	181 (100–300)	0·0 (0·0–0·1)
Laos	466 (100–1409)	0·0 (0·0–0·1)
Timor-Leste†	100 (100–100)	0·0 (0·0–0·0)
East Asia		
China	100 (100–100)	0·1 (0·1–0·1)
**Oceania**		
Australia and New Zealand		
Australia	44 (44–44)	0·0 (0·0–0·0)
Melanesia		
Papua New Guinea	10 (10–10)	0·0 (0·0–0·0)

Data are estimate (95% CI).

* Countries that have declared an emergency owing to human cases.

† Countries in which animals have tested positive for Nipah virus. Countries are listed within a region and a subregion; these geographical regions are based on continental divisions, which are further subdivided into subregions according to the UN standard country or area codes for statistical use.^52^

## Data Availability

All the data collected in this study are available in the appendixes.

## *Panel*: Research in context

### Evidence before this study

We searched PubMed and the Web of Science for articles published in English from April 30, 1999, to Dec 31, 2022, using the search terms “Nipah virus”, “Nipah”, “NiV”, “Henipavirus”, or “Hendra like virus”, in combination with “infection”, “outbreak”, “transmission”, “modelling”, “mapping”, “suitable environment”, “geographical distribution”, or “spatial distribution”. Additionally, we examined the reference lists of identified articles to find other pertinent papers that aligned with the objectives of our study. Of the seven papers we found, two focused on analysing the spatial distribution of infections (one of human infections in Bangladesh and one of bat infections in southeast Asia and Australia), one examined the potential for outbreak in and transmission to China, and four projected the risk of zoonotic Nipah virus transmission in south and southeast Asia using MaxEnt, Genetic Algorithm for Rule-set Prediction, an inhomogeneous Poisson model, and generalised boosted regression. However, these studies used data on Nipah virus outbreaks and animal seropositivity to address the overall risk of Nipah virus infection; an integrated assessment of transmission risk in terms of spillover and endemic infections was not conducted. Furthermore, despite the distinct habitats of different bat species within the genus Pteropus, previous studies have treated the entire genus as a single entity in their models, potentially overlooking variations in Nipah virus transmission risks. To our knowledge, no studies to date have explored Nipah virus infection across different geographical locations by integrating inferred geographical ancestral source and spatiotemporal diffusion dynamics, or investigated the complex interplay between multiple factors and their collective effect on population-level transmission dynamics of Nipah virus.

### Added value of this study

For the first time to our knowledge, we have established an integrated spatiotemporal and phylogenetic database of Nipah virus infections in humans and animals across south and southeast Asia since the first identified cases of Nipah virus infection in humans in 1998–99.. The overall adjusted casefatality rate was 71%, but varied considerably by country, ranging from 9% in Singapore to 78% in India. Additionally, we generated maps illustrating potential distribution patterns of Nipah virus through spillover events and in endemic areas. Temperature-related bioclimate variables and the probability of occurrence of Pteropus medius were important contributors to both the spillover and the endemic infection models.. Furthermore, we identified specific populations residing within regions predicted to be at risk of Nipah virus infection in endemic areas. In addition to having the largest predicted endemic area for potential infections, Bangladesh has the largest at-risk population, followed by India, Thailand, and Malaysia.

### Implications of all the available evidence

Regions with high predicted zoonotic exposure to Nipah virus and potential zoonotic niches suitable for Nipah virus transmission should prioritise the enhancement of zoonotic surveillance efforts and laboratory testing capacity. We strongly recommend the implementation of concurrent surveillance programmes in both human and animal populations under the guidance of One Health policy.

## References

[1] Chua KB, Goh KJ, Wong KT, Fatal encephalitis due to Nipah virus among pig-farmers in Malaysia. Lancet 1999; 354: 1257–59. 10520635 10.1016/S0140-6736(99)04299-3

[2] Murray K, Selleck P, Hooper P, A morbillivirus that caused fatal disease in horses and humans. Science 1995; 268: 94–97. 7701348 10.1126/science.7701348

[3] Marsh GA, de Jong C, Barr JA, Cedar virus: a novel Henipavirus isolated from Australian bats. PLoS Pathog 2012; 8: e1002836. 22879820 10.1371/journal.ppat.1002836 PMC3410871

[4] Drexler JF, Corman VM, Müller MA, Bats host major mammalian paramyxoviruses. Nat Commun 2012; 3: 796. 22531181 10.1038/ncomms1796 PMC3343228

[5] Madera S, Kistler A, Ranaivoson HC, Discovery and genomic characterization of a novel *Henipavirus*, Angavokely virus, from fruit bats in Madagascar. J Virol 2022; 96: e0092122. 36040175 10.1128/jvi.00921-22 PMC9517717

[6] Farrar JJ. Nipah-virus encephalitis—investigation of a new infection. Lancet 1999; 354: 1222–23. 10520625 10.1016/S0140-6736(99)90124-1

[7] Paton NI, Leo YS, Zaki SR, Outbreak of Nipah-virus infection among abattoir workers in Singapore. Lancet 1999; 354: 1253–56. 10520634 10.1016/S0140-6736(99)04379-2

[8] Nikolay B, Salje H, Hossain MJ, Transmission of Nipah virus—14 years of investigations in Bangladesh. N Engl J Med 2019; 380: 1804–14. 31067370 10.1056/NEJMoa1805376 PMC6547369

[9] Chadha MS, Comer JA, Lowe L, Nipah virus-associated encephalitis outbreak, Siliguri, India. Emerg Infect Dis 2006; 12: 235–40. 16494748 10.3201/eid1202.051247 PMC3373078

[10] Chatterjee P Nipah virus outbreak in India. Lancet 2018; 391: 2200. 31876482 10.1016/S0140-6736(18)31252-2

[11] WHO. Nipah baseline situation analysis. Aug 27, 2018. https://www.who.int/publications/m/item/nipah-baseline-situation-analysis (accessed Dec 16, 2022).

[12] WHO. Prioritizing diseases for research and development in emergency contexts. 2022. https://www.who.int/activities/prioritizing-diseases-for-research-and-development-in-emergency-contexts (accessed Dec 16, 2022).

[13] AbuBakar S, Chang LY, Ali AR, Sharifah SH, Yusoff K, Zamrod Z. Isolation and molecular identification of Nipah virus from pigs. Emerg Infect Dis 2004; 10: 2228–30. 15663869 10.3201/eid1012.040452 PMC3323361

[14] Gurley ES, Montgomery JM, Hossain MJ, Person-to-person transmission of Nipah virus in a Bangladeshi community. Emerg Infect Dis 2007; 13: 1031–37. 18214175 10.3201/eid1307.061128 PMC2878219

[15] Sazzad HM, Hossain MJ, Gurley ES, Nipah virus infection outbreak with nosocomial and corpse-to-human transmission, Bangladesh. Emerg Infect Dis 2013; 19: 210–17. 23347678 10.3201/eid1902.120971 PMC3559054

[16] Epstein JH, Anthony SJ, Islam A, Nipah virus dynamics in bats and implications for spillover to humans. Proc Natl Acad Sci USA 2020; 117: 29190–201. 33139552 10.1073/pnas.2000429117 PMC7682340

[17] Rahman MA, Hossain MJ, Sultana S, Date palm sap linked to Nipah virus outbreak in Bangladesh, 2008. Vector Borne Zoonotic Dis 2012; 12: 65–72. 21923274 10.1089/vbz.2011.0656

[18] Islam MS, Sazzad HM, Satter SM, Nipah virus transmission from bats to humans associated with drinking traditional liquor made from date palm sap, Bangladesh, 2011–2014. Emerg Infect Dis 2016; 22: 664–70. 26981928 10.3201/eid2204.151747 PMC4806957

[19] Ching PK, de los Reyes VC, Sucaldito MN, Outbreak of *Henipavirus* infection, Philippines, 2014. Emerg Infect Dis 2015; 21: 328–31. 25626011 10.3201/eid2102.141433 PMC4313660

[20] Reynes JM, Counor D, Ong S, Nipah virus in Lyle’s flying foxes, Cambodia. Emerg Infect Dis 2005; 11: 1042–47. 16022778 10.3201/eid1107.041350 PMC3371782

[21] Cappelle J, Hoem T, Hul V, Nipah virus circulation at human-bat interfaces, Cambodia. Bull World Health Organ 2020; 98: 539–47. 32773899 10.2471/BLT.20.254227 PMC7411325

[22] Epstein JH, Prakash V, Smith CS, *Henipavirus* infection in fruit bats (Pteropus giganteus), India. Emerg Infect Dis 2008; 14: 1309–11. 18680665 10.3201/eid1408.071492 PMC2600370

[23] Tsang SM, Wiantoro S, Veluz MJ, Dispersal out of Wallacea spurs diversification of *Pteropus* flying foxes, the world’s largest bats (Mammalia: Chiroptera). J Biogeogr 2020; 47: 527–37. 33041434 10.1111/jbi.13750 PMC7546435

[24] Plowright RK, Becker DJ, Crowley DE, Prioritizing surveillance of Nipah virus in India. PLoS Negl Trop Dis 2019; 13: e0007393. 31246966 10.1371/journal.pntd.0007393 PMC6597033

[25] Ambat AS, Zubair SM, Prasad N, Nipah virus: a review on epidemiological characteristics and outbreaks to inform public health decision making. J Infect Public Health 2019; 12: 634–39. 30808593 10.1016/j.jiph.2019.02.013

[26] Aditi SM, Shariff M. Nipah virus infection: a review. Epidemiol Infect 2019; 147: e95. 30869046 10.1017/S0950268819000086 PMC6518547

[27] Soman Pillai V, Krishna G, Valiya Veettil M. Nipah virus: past outbreaks and future containment. Viruses 2020; 12: 465. 32325930 10.3390/v12040465 PMC7232522

[28] Ang BSP, Lim TCC, Wang L. Nipah virus infection. J Clin Microbiol 2018; 56: e01875–17. 29643201 10.1128/JCM.01875-17 PMC5971524

[29] Denis M NIPHA Baseline Situation Analysis. 2018. https://cdn.who.int/media/docs/default-source/documents/health-topics/nipah/who_nipah_baseline_situation_analysis_27jan20188b415c76-8649-4e98-b18f-18c6134f3819.pdf?sfvrsn=8d3de3b2_1&download=true (accessed Dec 16 2022).

[30] US Centers for Disease Control & Prevention. Nipah virus: facts for clinicians. 2024. https://www.cdc.gov/nipah-virus/hcp/clinical-overview/ (accessed Jun 6, 2024).

[31] Institute of Epidemiology, Disease Control and Research. National guideline for management, prevention and control of Nipah virus infection including encephalitis. December, 2011. https://www.iedcr.org/pdf/files/nipah/National_Nipah.pdf (accessed Dec 16, 2022).

[32] Food and Agriculture Organization of the United Nations. Manual on the diagnosis of Nipah virus infection in animals. January, 2002. https://www.fao.org/3/ac449e/ac449e.pdf (accessed Dec 16, 2022).

[33] Hernández LHA, da Paz TYB, Silva SPD, First genomic evidence of a Henipa-like virus in Brazil. Viruses 2022; 14: 2167. 36298723 10.3390/v14102167 PMC9608811

[34] Iehlé C, Razafitrimo G, Razainirina J, *Henipavirus* and Tioman virus antibodies in pteropodid bats, Madagascar. Emerg Infect Dis 2007; 13: 159–61. 17370536 10.3201/eid1301.060791 PMC2725826

[35] Hayman DT, Wang LF, Barr J, Antibodies to Henipavirus or henipa-like viruses in domestic pigs in Ghana, West Africa. PLoS One 2011; 6: e25256. 21966471 10.1371/journal.pone.0025256 PMC3178620

[36] Atherstone C, Diederich S, Weingartl HM, Evidence of exposure to henipaviruses in domestic pigs in Uganda. Transbound Emerg Dis 2019; 66: 921–28. 30576076 10.1111/tbed.13105 PMC6849855

[37] Centers for Disease Control and Prevention. About Nipah virus. 2024. https://www.cdc.gov/nipah-virus/about/ (accessed Jun 6, 2024).

[38] Glennon EE, Restif O, Sbarbaro SR, Domesticated animals as hosts of henipaviruses and filoviruses: a systematic review. Vet J 2018; 233: 25–34. 29486875 10.1016/j.tvjl.2017.12.024

[39] Bouckaert R, Heled J, Kühnert D, BEAST 2: a software platform for Bayesian evolutionary analysis. PLoS Comput Biol 2014; 10: e1003537. 24722319 10.1371/journal.pcbi.1003537 PMC3985171

[40] Kalyaanamoorthy S, Minh BQ, Wong TKF, von Haeseler A, Jermiin LS. ModelFinder: fast model selection for accurate phylogenetic estimates. Nat Methods 2017; 14: 587–89. 28481363 10.1038/nmeth.4285 PMC5453245

[41] Rambaut A, Lam TT, Max Carvalho L, Pybus OG. Exploring the temporal structure of heterochronous sequences using TempEst (formerly Path-O-Gen). Virus Evol 2016; 2: vew007. 27774300 10.1093/ve/vew007 PMC4989882

[42] Rambaut A, Drummond AJ, Xie D, Baele G, Suchard MA. Posterior summarization in Bayesian phylogenetics using Tracer 1.7. Syst Biol 2018; 67: 901–04. 29718447 10.1093/sysbio/syy032 PMC6101584

[43] Bielejec F, Baele G, Vrancken B, Suchard MA, Rambaut A, Lemey P. SpreaD3: interactive visualization of spatiotemporal history and trait evolutionary processes. Mol Biol Evol 2016; 33: 2167–69. 27189542 10.1093/molbev/msw082 PMC6398721

[44] Schwarzer GCJ, Rücker G. Meta-analysis with R. Use R! 2015. https://github.com/guido-s/meta/(accessed Dec 16, 2022).

[45] McKee CD, Islam A, Luby SP, The ecology of Nipah virus in Bangladesh: a nexus of land use change and opportunistic feeding behavior in bats. Viruses 2021; 13: 169. 33498685 10.3390/v13020169 PMC7910977

[46] Gurley ES, Hegde ST, Hossain K, Convergence of humans, bats, trees, and culture in Nipah virus transmission, Bangladesh. Emerg Infect Dis 2017; 23: 1446–53. 28820130 10.3201/eid2309.161922 PMC5572889

[47] Hijmans RJ, Phillips S, Leathwick J, Elith J, Hijmans MRJ. Package ‘dismo’. Circles 2017; 9: 1–68.

[48] Elith J, Leathwick JR, Hastie T. A working guide to boosted regression trees. J Anim Ecol 2008; 77: 802–13. 18397250 10.1111/j.1365-2656.2008.01390.x

[49] Zhang YY, Sun YQ, Chen JJ, Mapping the global distribution of spotted fever group rickettsiae: a systematic review with modelling analysis. Lancet Digit Health 2023; 5: e5–15. 36424337 10.1016/S2589-7500(22)00212-6 PMC10039616

[50] Zhao GP, Wang YX, Fan ZW, Mapping ticks and tick-borne pathogens in China. Nat Commun 2021; 12: 1075. 33597544 10.1038/s41467-021-21375-1 PMC7889899

[51] Miao D, Dai K, Zhao GP, Mapping the global potential transmission hotspots for severe fever with thrombocytopenia syndrome by machine learning methods. Emerg Microbes Infect 2020; 9: 817–26. 32212956 10.1080/22221751.2020.1748521 PMC7241453

[52] UN Statistics Division. Standard country or area codes for statistical use. https://unstats.un.org/unsd/methodology/m49/ (accessed Dec 16, 2022).

[53] Deka MA, Morshed N. Mapping disease transmission risk of Nipah virus in south and southeast Asia. Trop Med Infect Dis 2018; 3: 57. 30274453 10.3390/tropicalmed3020057 PMC6073609

[54] Walsh MG. Mapping the risk of Nipah virus spillover into human populations in south and southeast Asia. Trans R Soc Trop Med Hyg 2015; 109: 563–71. 26179654 10.1093/trstmh/trv055

[55] Chaiyes A, Duengkae P, Suksavate W, Mapping risk of Nipah virus transmission from bats to humans in Thailand. EcoHealth 2022; 19: 175–89. 35657574 10.1007/s10393-022-01588-6 PMC10116436

[56] Mire CE, Satterfield BA, Geisbert JB, Pathogenic differences between Nipah virus Bangladesh and Malaysia strains in primates: implications for antibody therapy. Sci Rep 2016; 6: 30916. 27484128 10.1038/srep30916 PMC4971471

[57] Clayton BA, Middleton D, Bergfeld J, Transmission routes for Nipah virus from Malaysia and Bangladesh. Emerg Infect Dis 2012; 18: 1983–93. 23171621 10.3201/eid1812.120875 PMC3557903

[58] DeBuysscher BL, de Wit E, Munster VJ, Scott D, Feldmann H, Prescott J. Comparison of the pathogenicity of Nipah virus isolates from Bangladesh and Malaysia in the Syrian hamster. PLoS Negl Trop Dis 2013; 7: e2024. 23342177 10.1371/journal.pntd.0002024 PMC3547834

[59] Klein SL, Flanagan KL. Sex differences in immune responses. Nat Rev Immunol 2016; 16: 626–38. 27546235 10.1038/nri.2016.90

[60] Gonzalez V, Banerjee A. Molecular, ecological, and behavioral drivers of the bat-virus relationship. iScience 2022; 25: 104779. 35875684 10.1016/j.isci.2022.104779 PMC9296223

[61] Frick WF, Stepanian PM, Kelly JF, Climate and weather impact timing of emergence of bats. PLoS One 2012; 7: e42737. 22876331 10.1371/journal.pone.0042737 PMC3411708

[62] Luis AD, O’Shea TJ, Hayman DTS, Network analysis of host-virus communities in bats and rodents reveals determinants of cross-species transmission. Ecol Lett 2015; 18: 1153–62. 26299267 10.1111/ele.12491 PMC5014217

[63] Beale CM, Lennon JJ. Incorporating uncertainty in predictive species distribution modelling. Philos Trans R Soc Lond B Biol Sci 2012; 367: 247–58. 22144387 10.1098/rstb.2011.0178 PMC3223803

[64] Hegde ST, Sazzad HM, Hossain MJ, Investigating rare risk factors for Nipah virus in Bangladesh: 2001–2012. EcoHealth 2016; 13: 720–28. 27738775 10.1007/s10393-016-1166-0 PMC5164848

[65] Coker RJ, Hunter BM, Rudge JW, Liverani M, Hanvoravongchai P. Emerging infectious diseases in southeast Asia: regional challenges to control. Lancet 2011; 377: 599–609. 21269678 10.1016/S0140-6736(10)62004-1 PMC7159088

