# Supplementary material for: Mapping the distribution of Nipah virus infections: a geospatial modelling analysis
Source: Lancet Planet Health. Author manuscript; Available in PMC 2025 Oct 10. (PMC12513742; doi:10.1016/S2542-5196(24)00119-0)
Supplement: Supplementary appendix [file NIHMS2109452-supplement-Supplementary_appendix.docx]

**Supplementary appendix**

Supplement to: Mapping the distribution of Nipah virus infections: a geospatial modelling analysis

**Table of Contents**

[Appendix Text S1: Phylogenetic and phylogeographic analysis	5](#_Toc161734201)

[Appendix Text S2: The process of meta-analysis for case fatality rate (CFR), clinical manifestation proportion and positive detection rate	7](#_Toc161734202)

[Appendix Text S3: The ecological modelling of date palms, NiV reservoir hosts and NiV infections	9](#_Toc161734203)

[Appendix Text S4: Demographic characteristics of patients and temporal pattern of NiV infections	14](#_Toc161734204)

[Appendix Table S1: PRISMA checklist	15](#_Toc161734205)

[Appendix Table S2: Literature search syntax of NiV	18](#_Toc161734206)

[Appendix Table S3: Information of NiV infections from grey literatures	19](#_Toc161734207)

[Appendix Table S4: Guideline for inclusion and exclusion of literatures of NiV infections	22](#_Toc161734208)

[Appendix Table S5: Criteria of human and animal infection with NiV	23](#_Toc161734209)

[Appendix Table S6: Data extracted from literature	24](#_Toc161734210)

[Appendix Table S7: Genome sequence used in the phylogenetic and phylogeographic analysis	25](#_Toc161734211)

[Appendix Table S8: Source of data used in the modelling analysis	28](#_Toc161734212)

[Appendix Table S9: Description of 50 potential predictive variables used in the modelling	30](#_Toc161734213)

[Appendix Table S10: Ecological factors potentially associated with the bat and bat-borne pathogens used in the modelling analysis.	32](#_Toc161734214)

[Appendix Table S11: Occurrence data for bats using to build the ecological niche model	42](#_Toc161734215)

[Appendix Table S12 Definition of NiV infection in reservoir hosts	43](#_Toc161734216)

[Appendix Table S13: Risk of bias assessments for the meta-analysis of CFR and clinical manifestation proportions	44](#_Toc161734217)

[Appendix Table S14: Subgroup meta-analysis and meta-regression of the case fatality rates of NiV infection	46](#_Toc161734218)

[Appendix Table S15: Sensitivity analysis of meta-analysis of the case fatality rates of NiV infection	49](#_Toc161734219)

[Appendix Table S16: Subgroup meta-analysis of NiV positive detection rate in reservoir hosts	50](#_Toc161734220)

[Appendix Table S17: Locations with NiV infection in animals and humans used to build BRT models	51](#_Toc161734221)

[Appendix Table S18: Literature search syntax of bat-borne virus in PubMed in the study area	56](#_Toc161734222)

[Appendix Table S19: Mean (95% percentiles) relative contributions of variables (95% percentiles) of host niche models in this study	57](#_Toc161734223)

[Appendix Table S20: Mean (95% percentiles) relative contributions of variables (95% percentiles) of NiV models	59](#_Toc161734224)

[Appendix Table S21: Comparison of performance metrics across different cutoff methods for Binary Classification in the NiV endemic infection model using the R “OptimalCutpoints” package.	61](#_Toc161734225)

[Appendix Table S22: The absolute out-of-sample performance between ecological model and sensitivity analysis.	62](#_Toc161734226)

[Appendix Figure S1: Timeline of NiV cases from 1999 to 2021 (A) and radar chart (B) illustrating the monthly cumulative NiV case numbers.	63](#_Toc161734227)

[Appendix Figure S2: Chord diagram representing affected countries and host diversity according to the infectious cases.	64](#_Toc161734228)

[Appendix Figure S3: Geographical distribution of eight bat species linked to NiV infection	65](#_Toc161734229)

[Appendix Figure S4: Root-to-tip plot of the genetic distance against sampling time of the NiV phylogeny.	66](#_Toc161734230)

[Appendix Figure S5: Bayesian skyline plot of effective population size.	67](#_Toc161734231)

[Appendix Figure S6: Case fatality rates of NiV infection based on subgroup meta-analysis by clades.	68](#_Toc161734232)

[Appendix Figure S7: Case fatality rates of NiV infection based on subgroup meta-analysis by countries	69](#_Toc161734233)

[Appendix Figure S8: Case fatality rates of NiV infection based on subgroup meta-analysis by publish year	70](#_Toc161734234)

[Appendix Figure S9: Case fatality rates of NiV infection based on subgroup meta-analysis by mean age	71](#_Toc161734235)

[Appendix Figure S10: Case fatality rates of NiV infection based on subgroup meta-analysis by male proportion	72](#_Toc161734236)

[Appendix Figure S11: Case fatality rates of NiV infection based on subgroup meta-analysis by risk of bias	73](#_Toc161734237)

[Appendix Figure S12: Case fatality rates of NiV infection based on subgroup meta-analysis by GDP per capita	74](#_Toc161734238)

[Appendix Figure S13: Case fatality rates of NiV infection based on subgroup meta-analysis by HDI index	75](#_Toc161734239)

[Appendix Figure S14: Case fatality rates of NiV infection based on subgroup meta-analysis by Gini index	76](#_Toc161734240)

[Appendix Figure S15: Case fatality rates of NiV infection based on subgroup meta-analysis by travel time to healthcare facilities	77](#_Toc161734241)

[Appendix Figure S16: Funnel plot of meta-analysis of CFR (not sub-grouped)	78](#_Toc161734242)

[Appendix Figure S17: Funnel plots of subgroup meta-analysis of CFR.	79](#_Toc161734243)

[Appendix Figure S18: Bubble plot with a fitted meta-regression for the variation of the case fatality rate of NiV infection.	81](#_Toc161734244)

[Appendix Figure S19: Meta-analysis of clinical manifestation proportion.	82](#_Toc161734245)

[Appendix Figure S20: Funnel plots of clinical subgroup meta-proportion of NiV infections.	90](#_Toc161734246)

[Appendix Figure S21: Positive detection rate of NiV in reservoir hosts based on subgroup meta-analysis.	92](#_Toc161734247)

[Appendix Figure S22: Funnel plot of meta-analysis of NiV detection rate in reservoir hosts (not sub-grouped)	93](#_Toc161734248)

[Appendix Figure S23: Funnel plots of subgroup meta-analysis of NiV detection rate in reservoir hosts.	94](#_Toc161734249)

[Appendix Figure S24: Geographic range and occurrences of](#_Toc161734250) *[Pteropus lylei](#_Toc161734250)* [according to IUCN Red List of Threatened Species.	95](#_Toc161734250)

[Appendix Figure S25: Geographic range and occurrences of](#_Toc161734251) *[Pteropus medius](#_Toc161734251)* [according to IUCN Red List of Threatened Species.	96](#_Toc161734251)

[Appendix Figure S26: Geographic range and occurrences of](#_Toc161734252) *[Pteropus vampyrus](#_Toc161734252)* [according to IUCN Red List of Threatened Species.	97](#_Toc161734252)

[Appendix Figure S27: Geographic range and occurrences of](#_Toc161734253) *[Pteropus hypomelanus](#_Toc161734253)* [according to IUCN Red List of Threatened Species.	98](#_Toc161734253)

[Appendix Figure S28: Geographic range and occurrences of](#_Toc161734254) *[Rousettus leschenaultii](#_Toc161734254)* [according to IUCN Red List of Threatened Species.	99](#_Toc161734254)

[Appendix Figure S29: Geographic range and occurrences of](#_Toc161734255) *[Rousettus amplexicaudatus](#_Toc161734255)* [according to IUCN Red List of Threatened Species.	100](#_Toc161734255)

[Appendix Figure S30: Geographic range and occurrences of](#_Toc161734256) *[Hipposideros larvatus](#_Toc161734256)* [according to IUCN Red List of Threatened Species.	101](#_Toc161734256)

[Appendix Figure S31: Geographic range and occurrences of](#_Toc161734257) *[Pipistrellus pipistrellus](#_Toc161734257)* [according to IUCN Red List of Threatened Species.	102](#_Toc161734257)

[Appendix Figure S32: Palm occurrences used as the background to generate pseudo-absence data in the date palms (](#_Toc161734258)*[Phoenix sylvestris](#_Toc161734258)*[) ecological niche models.	103](#_Toc161734258)

[Appendix Figure S33: Bat occurrences used as the background to generate pseudo-absence data in the host ecological niche models.	104](#_Toc161734259)

[Appendix Figure S34: Human virus occurrences used as the background to generate pseudo-absence data in the NiV spillover model.	105](#_Toc161734260)

[Appendix Figure S35: Bat-borne virus occurrences used as the background to generate pseudo-absence data in the NiV reservoir host model.	106](#_Toc161734261)

[Appendix Figure S36: Predicted ecological niche of date palms (](#_Toc161734262)*[Phoenix sylvestris](#_Toc161734262)*[).	107](#_Toc161734262)

[Appendix Figure S37: Predicted ecological niches of NiV reservoir hosts.	108](#_Toc161734263)

[Appendix Figure S38: Uncertainty maps for ecological niche model of NiV reservoir hosts	109](#_Toc161734264)

[Appendix Figure S39: The relative contribution of top nine predictors and response curve in the BRT models for](#_Toc161734265) *[Pteropus lylei](#_Toc161734265)*[.	110](#_Toc161734265)

[Appendix Figure S40: The relative contribution and response curve of top nine predictors in the BRT models for](#_Toc161734266) *[Pteropus medius](#_Toc161734266)*	[111](#_Toc161734266)

[Appendix Figure S41: The relative contribution and response curve of top nine predictors in the BRT models for](#_Toc161734267) *[Pteropus vampyrus](#_Toc161734267)*[.	112](#_Toc161734267)

[Appendix Figure S42: The relative contribution and response curve of top nine predictors in the BRT models for](#_Toc161734268) *[Pteropus hypomelanus](#_Toc161734268)*[.	113](#_Toc161734268)

[Appendix Figure S43: The relative contribution and response curve of top nine predictors in the BRT models for](#_Toc161734269) *[Rousettus leschenaultii](#_Toc161734269)*[.	114](#_Toc161734269)

[Appendix Figure S44: The relative contribution and response curve of top nine predictors in the BRT models for](#_Toc161734270) *[Rousettus amplexicaudatus](#_Toc161734270)*[.	115](#_Toc161734270)

[Appendix Figure S45: The relative contribution and response curve of top nine predictors in the BRT models for](#_Toc161734271) *[Hipposideros larvatus](#_Toc161734271)*[.	116](#_Toc161734271)

[Appendix Figure S46: The relative contribution and response curve of top nine predictors in the NiV spillover model.	117](#_Toc161734272)

[Appendix Figure S47: The relative contribution and response curve of top nine predictors in the NiV endemic infection model.	118](#_Toc161734273)

[Appendix Figure S48: Predictive performance of ecological niches of NiV reservoir hosts.	119](#_Toc161734274)

[Appendix Figure S49: Predictive performance of ecological niches of NiV	120](#_Toc161734275)

[Appendix Figure S50: Geographic representation of binary risky areas based on NiV endemic infection model.	121](#_Toc161734276)

[Appendix Figure S51: The sensitivity analysis of ecological niche model.	122](#_Toc161734277)

[Appendix Figure S52: Plots of two variables which may be related to NiV transmission.	123](#_Toc161734278)

[Appendix Figure S53: Plots of main covariates (except for bats) in the BRT model	124](#_Toc161734279)

[Appendix Text S5: References included in the NiV study (Reference ID)	125](#_Toc161734280)

[Appendix Text S6: References searched out in PubMed for the distribution of bat reservoirs	131](#_Toc161734281)

[Appendix Text S7: References searched out in CNKI for the distribution of bat reservoirs (in Chinese)	134](#_Toc161734282)

[Appendix Text S8: References searched out in PubMed for the occurrence of bat-borne viruses	136](#_Toc161734283)

# Appendix Text S1: Phylogenetic and phylogeographic analysis

**Download and selection of NiV genomes**

A comprehensive collection of NiV genome records was initiated to facilitate thorough phylogenetic and phylogeographic analyses with extensive spatiotemporal coverage. In this endeavor, a total of 92 complete (18,027–18,252 bp) and nearly complete (>17,000 bp) NiV genome sequences were meticulously obtained from GenBank as of November 21, 2022. Stringent criteria were applied for sequence selection, involving the exclusion of patent sequences, NCBI redundant entries, and unverified data. The process aimed to ensure robustness and reliability of the genomic dataset. For complete genome sequences, stringent length and quality thresholds were employed. Sequences with lengths greater than or equal to 0·9 of the full length (18246 bp) and less than 110 degenerate bases were retained, resulting in a final set of 66 complete genome sequences. To enhance dataset comprehensiveness, we supplemented our sequences with data from literature sources, including host, collection time, and geographic location. For sequences lacking specific collection dates, we imputed mid-year (July 1st) or mid-month (15th) dates if only year or year and month were available, respectively.

**Evolution analysis of NiV using BEAST2**

A comprehensive evolutionary analysis of NiV was conducted using BEAST2 v 2.6.3,^1^ employing the Bayesian stochastic search variable selection (BSSVS) approach. ModelFinder was utilized to identify the best-fitting model (GTR+F+G4) for maximum likelihood analyses.^2^ This model was subsequently employed to conduct maximum likelihood analyses and construct an ML tree. TempEst v1.5.3 was utilized to explore the temporal structure of NiV complete genomes.^3^

The Bayesian phylogeny construction involved the utilization of a general time reversible substitution model with gamma distribution and invariant sites (GTR+F+G4), an uncorrelated log-normal relaxed molecular clock model, and a Bayesian skyline prior. Bayesian analyses were run for 600 million Markov Chain Monte Carlo (MCMC) steps, with parameters and trees sampled every 20,000 generations. After confirming convergence of the chains, the output files were combined using LogCombiner, with a 10% burn-in removed. Convergence of MCMC chains was further evaluated by calculating effective sample sizes of parameters using Tracer v1.7.1 (http://beast.bio.ed.ac.uk/Tracer),^4^ ensuring values >200 for all parameters. Clock rate and tMRCA estimates were obtained using Tracer v1.7.1, and the maximum clade credibility (MCC) tree was identified using TreeAnnotator v1.8.4 (node height = median) after discarding the initial 10% burn-in.^5^ The resulting tree was visualized using FigTree v1.4.4 (http://tree.bio.ed.ac.uk/software/figtree/), while phylogeographic transmission routes were visualized using spreaD3 v0.9.7.^6^

**Reference**:

1.	Bouckaert R, Heled J, Kühnert D, et al. BEAST 2: a software platform for Bayesian evolutionary analysis. *PLoS computational biology* 2014; **10**: e1003537.

2.	Kalyaanamoorthy S, Minh BQ, Wong TKF, von Haeseler A, Jermiin LS. ModelFinder: fast model selection for accurate phylogenetic estimates. *Nat Methods* 2017; **14**: 587-9.

3.	Rambaut A, Lam TT, Max Carvalho L, Pybus OG. Exploring the temporal structure of heterochronous sequences using TempEst (formerly Path-O-Gen). *Virus evolution* 2016; **2**: vew007.

4.	Rambaut A, Drummond AJ, Xie D, Baele G, Suchard MA. Posterior summarization in Bayesian phylogenetics using Tracer 1.7. *Systematic biology* 2018; **67**: 901-4.

5.	Drummond AJ, Rambaut A. BEAST: Bayesian evolutionary analysis by sampling trees. *BMC evolutionary biology* 2007; **7**: 1-8.

6.	Bielejec F, Baele G, Vrancken B, Suchard MA, Rambaut A, Lemey P. SpreaD3: interactive visualization of spatiotemporal history and trait evolutionary processes. *Molecular biology and evolution* 2016; **33**: 2167-9.

# Appendix Text S2: The process of meta-analysis for case fatality rate (CFR), clinical manifestation proportion and positive detection rate

**Method**

We followed the Preferred Reporting Items for Systematic Reviews and Meta-analysis (PRISMA) statement.1 The protocol was registered with PROSPERO (CRD42023390049).

In the meta-analysis, we examined three outcome variables: case fatality rate (CFR), clinical manifestation proportions (CMP), and positive detection rate of NiV in reservoir hosts (PDR).

(1) CFR: The CFR is calculated as the proportion of the number of deaths attributed to NiV infection to the total number of diagnosed cases, represented by the formula:

$$CFR=\frac{Number of Deaths}{Number of cases}$$

Where: "Number of Deaths" refers to the total number of deaths attributed to NiV infection. "Number of Cases" refers to the total number of cases with NiV infections

(2) CMR: The specific clinical manifestation proportion is calculated as the proportion of cases displaying a specific clinical manifestation relative to the total number of cases, represented by the formula:

$$CMP=\frac{Number of cases with specific clinical manifestation}{Total number of cases}$$

Where: "Number of cases with specific clinical manifestation" refers to the count of cases exhibiting the particular clinical manifestation being considered. "Total number of cases" refers to the overall count of diagnosed cases included in the analysis.

(3) PDR: The positive detection rate of NiV in reservoir hosts is calculated as the proportion of cases displaying a positive detection animal number of NiV relative to the total number of tested animals, represented by the formula:

$$PDR=\frac{Number of Reservoir Hosts Testing Positive}{Total number of tested animals}$$

Where: "Number of Reservoir Hosts Testing Positive " refers to the number of reservoir hosts testing positive for NiV infection based on guidelines by FAO and OIE. "Total number of tested animals" refers to the total number of tested animals.

Two investigators (YN Liu and LJ Yu) independently extracted CFR, clinical manifestation information and study characteristics by using a standardized form. Due to the lack of a specific tool for assessing the bias of three proportions (CFR and CMR in human cases and PDR in reservoir hosts), we resort to utilizing the Joanna Briggs Institute (JBI) critical appraisal tool to evaluate the bias risk in the literature.^2^ Two reviewers (YY Zhang and MC Liu) independently completed the assessments for each study. Conflicts were resolved by discussion and consensus among the two authors. The two investigators cross-verified the data collected for possible errors. If the proportion in a study was reported as zero, we added a continuity correction of 0·5 to the number of positive cases before statistical analysis. Freeman-Tukey double arcsine transformation was used to stabilize the variances of three proportions before weighting measures using the inverse variance method. Heterogeneity was quantified using the statistic Higgin’s *I*², and pooled three proportions and associated 95% CIs were then estimated using DerSimonian and Laird random-effects models (REM) to factor the geographical and study-level heterogeneities if the value of Higgin’s *I*² was greater than 50%, otherwise, fixed effects model was applied.

We performed subgroup meta-analyses, as well as univariable and multivariable meta-regression analyses of CFR, to explore possible sources of heterogeneity and examine the effects of study characteristics (viral clade, country, year of publication, mean/median age and proportion of male patients, and risk of bias) and four provincial-level indices (travel time to healthcare facilities, GDP per capita, human development index, and Gini index) associated with access to healthcare, poverty, social vulnerability, and income inequality.^3–6^ The results of the meta-regression analysis, including regression coefficients, 95% CIs, and *p* values, were reported. To enhance the robustness of the multivariable model, a two-step approach was used. Firstly, we incorporated factors with *p* values less than 0·20 in the univariable model into the multivariable model. Secondly, based on which those with *p* values less than 0·10 were reincorporated into the multivariable model.

The coefficient of determination (R^2^) was also calculated to indicate the percentage of variance explained by the effect modifiers. Permutation testing was performed for the meta-regression to eliminate possible false positives and minimize spurious results. Publication bias was assessed by funnel plots of outcomes. Bubble plots were conducted to show the estimated regression slope, as well as the effect size of each study. We also conducted sensitivity analysis to assess the robustness of pooled estimates by removing each study through the “leave-one-out” method.

Meta-analysis was carried out using the “metaprop” function in the R packages “meta”, “metafor”, “dmetar”.^7–9^ Four provincial-level indices (GDP, population, Gini index and travel time to healthcare facilities) were extracted from gridded map by using the R package “extractextractr”.^10^

**Reference**:

1. Page MJ, McKenzie JE, Bossuyt PM, et al. The PRISMA 2020 statement: an updated guideline for reporting systematic reviews. *BMJ*. 2021; **372**: n71.

2. Moola S, Munn Z, Tufanaru C, Aromataris E, Sears K, Sfetcu R, Currie M, Lisy K, Qureshi R, Mattis P, Mu P. Chapter 7: Systematic reviews of etiology and risk. In: Aromataris E, Munn Z (Editors). JBI Manual for Evidence Synthesis. *JBI*, 2020.

3. Weiss DJ, Nelson A, Vargas-Ruiz CA, et al. Global maps of travel time to healthcare facilities. *Nat Med*. 2020; **26**: 1835–1838.

4. Chen J, Gao M, Cheng S, et al. Global 1 km × 1 km gridded revised real gross domestic product and electricity consumption during 1992–2019 based on calibrated nighttime light data. *Sci Data*. 2022; **9**: 202.

5. Data retrieved from the Subnational HDI Database of the Global Data Lab, https://globaldatalab.org/shdi/, version v5.0.

6. Mirza MU, Xu C, Bavel BV, van Nes EH, Scheffer M. Global inequality remotely sensed. *Proc Natl Acad Sci U S A*. 2021;**118**: e1919913118.

7. Schwarzer G CJ, Rücker G Meta-Analysis with R. Use R! 2015. https://github.com/guido-s/meta/.

8. Viechtbauer W. Conducting meta-analysis in R with the metafor package. *J Stat Softw* 2010; **36**: 1–48.

9. Harrer M, Cuijpers P, Furukawa TA, Ebert DD. Doing meta-analysis with R: a hands-on guide. 1st ed. Boca Raton, FL and London: Chapman & Hall/CRC Press; 2021.

10. Baston D, ISciences L, Baston MD. Package ‘exactextractr’. *terra* 2022; **1**: 17.

# Appendix Text S3: The ecological modelling of date palms, NiV reservoir hosts and NiV infections

**Study area of NiV distribution**

We included the study areas covering the countries or regions where NiV is currently known to occur or the relevant bat species are found in South Asia, South-East Asia, East Asia and Oceania. These areas mainly included India, Pakistan, Bangladesh, Nepal, Bhutan, Sri Lanka, Indonesia, Philippines, Vietnam, Thailand, Myanmar, Malaysia, Cambodia, Lao, Singapore, East Timor, Brunei, as well as three countries (China, Australia, and Papua New Guinea) due to the extensive range of some of the reservoir bat species. All analysis was focused on these regions as defined above.

**Data processing of date palms (*Phoenix sylvestris*)**

Due to the present investigation suggested that date palms sap is an important pathway for NiV transmission, we first built the ecological niche model of date palms (*Phoenix sylvestris*). Occurrence data for palms (family Arecaceae) were sourced from reputable databases, including the Global Biodiversity Information Facility (GBIF) (https://www.gbif.org), Integrated Digitized Biocollections (iDigBio) (https://www.idigbio.org), and iNaturalist (https://www.inaturalist.org) (last update: Dec 10, 2023). Subsequently, data refinement was conducted using the R package “CoordinateCleaner,” resulting in 435 720 palm locations, with 1045 identified as *Phoenix sylvestris*. These refined locations formed the basis for constructing the ecological niche model.

**Data processing of NiV reservoir host occurrence**

To account for drivers of NiV transmission associated with reservoir hosts in the model, we aimed to predict the distributions of bat species linked to NiV infections. Occurrence data of these bat species was obtained from several databases or datasets including DarkCideS 1.0 (https://darkcides.org/), Bat Eco-Interaction (https://www.batbase.org/), GBIF, iDigBio, and iNaturalist. Additionally, a search of peer-reviewed literature was conducted using the species name to locate further reservoir host occurrence data (search terms for each reservoir bat were included in PubMed and CNKI, with a last update of Nov 21, 2022, details in appendix pp 131–135). The data were verified for taxonomic and range consistency using the calibration area by buffering the shapefile coverage from the IUCN Red List of Threatened Species (last update: Dec 10, 2022) by 100 km. To minimize spatial sampling biases due to uneven and under-sampling, a series of filtering steps were performed using the R package “CoordinateCleaner”,^1^ which removed data concentrated in political centroids of countries, provinces, national capitals, centroids for GBIF headquarters and museums, and duplicates in permanent water bodies and oceans. The IUCN shapefile of *Pipistrellus pipistrellus* was located outside of the study area, so we excluded this species from subsequent analysis. The potential distribution was predicted for seven bat species, by using data from 119 locations for *Pteropus lylei,* 883 for *Pteropus medius*, 390 for *Pteropus vampyrus*, 131 locations for *Pteropus hypomelanus*, 265 for *Rousettus leschenaultii*, 679 for *Rousettus amplexicaudatus*, and 366 for *Hipposideros larvatus* (appendix p 42).

**Georeference**

The point locations and polygons less than one square degree (about 110 km × 110 km in areas at the equator) of NiV occurrence were georeferenced using Google Map, and all occurrence data underwent manual checking and quality control to ensure the validity and accuracy of geo-positioning. Locations with only person-to-person transmission were excluded. All maps were produced using the R packages “ggplot2” and “sf”.^2,3^

**BRT modelling approach**

We collected 42 variables, along with predicted ecological niches of date palms and seven reservoir hosts, for a total of 50 variables which reflect the potential drivers of NiV occurrence. These variables were categorized into three types: 31 environmental related variables, nine human activity related variables, and ten animal related variables. The full details of data sources, original resolutions and rescaling are presented in appendix pp 28–31. Environmental related variables and two human activity related variables (cropland and urbanland) were used both in the ecological niche model of date palms and NiV reservoir hosts, and one animal related variables (Chiroptera richness) was also used in the NiV reservoir hosts models.

The boosted regression tree (BRT) approach was employed using the R package “dismo” to perform ecological model of date palms niche, NiV reservoir hosts’ niches and NiV infections.^4^ In the NiV ecological modelling, we developed two distinct models: a spillover model and an endemic infection model. The spillover model specifically identifies areas conducive to NiV spillover by focusing on the human index case/confirmed spillover event for each outbreak, while excluding any outbreaks or human cases involving only with human-to-human transmission or transmission from intermediary hosts to humans; whereas the endemic infection model evaluates areas with NiV presence in humans, intermediary hosts and reservoir hosts while excluding outbreak events or cases solely through human-to-human transmission or only between intermediary hosts.

Each occurrence data was matched to the grid-map according to its coordinate and only one presence grid was counted if multiple records were associated with the same grid. Pseudo-absence data (defined as “controls”) provide a sample of the set of conditions available of the species in the region rather than actual absences. To account for reporting bias in presence data (defined as “cases”), we followed Phillips et al. in sampling pseudo-absence data according to the same reporting bias likely to be present in occurrence records.^5^ For date palms, reservoir host and NiV infections modelling, we selected pseudo-absence data which was 10 times the number of date palms data from three datasets (GBIF, iDigBio and iNaturalist, see appendix p 103 for visualization), reservoir host presence data from five bat datasets (DarkCideS 1.0, Bat Eco-Interaction, GBIF, iDigBio and iNaturalist, omitting the records of the reservoir host, see appendix p 104 for visualization); for the two models of NiV infections, we selected pseudo-absence data from two human virus occurrence datasets (dengue occurrences by Messina JP et al.^6^, and Chikungunya occurrences by Tjaden NB et al.^7^, see appendix p 105 for visualization) in the spillover model, both human virus datasets and four bat-borne virus datasets (including coronavirus occurrences by Wang Z et al.^8^ and Warmuth VM et al.^9^, Global Biotic Interaction,^10^ PREDICT project in USAID’s Emerging Pandemic Threats program,^11^ and occurrences dataset by searching terms (search syntax in appendix p 56) in PubMed, see appendix p 106 for visualization) in the endemic infection model, respectively, which represented as a proxy for observation bias. We also applied a 20 km filtering distance as buffer zone to avoid excessive clustering within any particular region, as the known reservoir bats move from roost sites to foraging sites within or close this range.^12–16^

**Regularization and Robustness**

Regularization plays a critical role in Boosted Regression Tree (BRT) models due to their sequential model fitting, which permits the addition of trees until the data become excessively overfitted.^17^ Overfitting to training data diminishes the model's generality, necessitating the use of regularization methods to regulate the fitting process and achieve a balance between model fit and predictive performance. BRT regularization entails the concurrent optimization of the number of trees (nt), learning rate (lr), and tree complexity (tc) parameters.^17^ BRT models with cross-validation based (CV-based) selection of the number of trees, shrinkage, bagging, and relative contribution techniques effectively account for correlations between variables while preventing overfitting and improving model performance.^17,18^

In CV-based selection of the number of trees, the dataset is split into multiple folds. The model is trained on different combinations of folds and evaluated on the remaining fold(s) iteratively.^18^ CV provides a means for testing the model on withheld portions of data, while still using all data at some stage to fit the model. This technique helps in assessing the model's generalization ability and reduces the risk of overfitting to the training data.

The shrinkage or learning rate parameter controls the contribution of each tree to the final model. A smaller learning rate effectively shrinks the contribution of each individual tree, reducing the impact of overfitting. By introducing a learning rate less than 1, the algorithm becomes more conservative in updating the model with each tree, which helps prevent overfitting and allows the model to better capture the underlying patterns in the data, including correlations between variables.^17^

BRT models often introduce randomness through a process called bagging (bootstrap aggregating) or by using a subset of the data for each tree. This helps reduce overfitting by promoting model diversity. The "bag fraction" parameter controls the proportion of the dataset used for training each individual tree. By varying the bag fraction and comparing predictive performance, optimal bag fractions can be determined to balance model complexity and performance. Introducing randomness in the training process helps the model generalize better to unseen data and reduces the impact of noise and spurious correlations.^17^

BRT models can assess the relative contribution of each variable to the predictive performance of the model. Variables with less useful predictive information may have lower relative contributions and can be effectively excluded from the final model. By focusing on variables with higher relative contributions, the model can prioritize important predictors while disregarding less relevant ones, even in the presence of correlations between variables.^17^

**Validation and Modelling**

A tree complexity of five, cross-validation folds of 10, a learning rate of 0·005 and a bagging fraction of 75% were used for the primary analysis based on their satisfactory performance in our previous research.^19–21^ A training set with 80% of the data points was randomly sampled without replacement, and the remaining 20% served as the test set. To select the optimal number of trees in the BRT models, the CV step and the assessment with “gbm.perf” was performed to make model regularization.

To increase the robustness of the model predictions and quantify the uncertainty, we fitted an ensemble of 100 BRT sub-models. Each sub-model was fitted to a separate bootstrap resampling of the dataset to determine the number of trees that maximized the cross-validation predictive accuracy. This was used to generate a probability map for each species on a 10 km × 10 km resolution grid. The standard deviation of each grid cell was used as an uncertainty metric. The relative contributions (RCs) of all predictors and the area-under-curve (AUC) of receiver operating characteristic (ROC) curves for test sets were averaged over the 100 sub-models in the assembly to represent the final estimation results and predictive performance of the model assembly using the R packages “ROCR” and “pROC”.^22,23^

To identify high-risk areas for Nipah virus (NiV) zoonotic transmission, we optimized our predictive model by selecting the threshold that achieved the greatest combination of sensitivity and specificity on the Receiver Operating Characteristic (ROC) curve. This process involved a comparative analysis of performance metrics across various thresholding techniques using the “OptimalCutpoints” package in R. We ultimately determined the optimal cutoff value based on the largest kappa statistic, enhancing the generalizability of our findings. Grids with predicted probabilities above the cut-off value for the final model were considered as having a high risk of NiV zoonotic infections. We estimated the vulnerability of human populations by overlaying population datasets with maps of suitable areas predicted to be a zoonotic niche of NiV.

**Sensitivity analysis**

To further assess model robustness, we randomly subsampled 80% of the presence data and selected the top 20 predictor variables based on their contribution to model performance and ecological relevance. Ecological niche models were retrained using the selected predictor variables from each subsample, and absolute out-of-sample performance metrics, including Mean Absolute Error (MAE), Mean Squared Error (MSE), Root Mean Squared Error (RMSE), and coefficient of determination (R²), were calculated.

**Reference**:

1. Zizka A, Silvestro D, Andermann T, et al. CoordinateCleaner: Standardized cleaning of occurrence records from biological collection databases. *Methods Ecol Evol* 2019; **10**: 744–51.

2. Wickham H, Chang W, Wickham MH. Package ‘ggplot2’. Create elegant data visualisations using the grammar of graphics Version 2016; **2**: 1–189.

3. Pebesma EJ. Simple features for R: standardized support for spatial vector data. *R J* 2018; **10**: 439.

4. Hijmans RJ, Phillips S, Leathwick J, Elith J, Hijmans MRJ. Package ‘dismo’. *Circles* 2017; **9**: 1–68.

5. Phillips SJ, Dudík M, Elith J, et al. Sample selection bias and presence-only distribution models: implications for background and pseudo-absence data. *Ecol Appl*. 2009; **19**: 181-197.

6. Messina JP, Brady OJ, Golding N, et al. The current and future global distribution and population at risk of dengue. *Nat Microbiol*. 2019; **4**: 1508-1515.

7. Tjaden NB, Suk JE, Fischer D, Thomas SM, Beierkuhnlein C, Semenza JC. Modelling the effects of global climate change on Chikungunya transmission in the 21st century. *Sci Rep*. 2017; **7**: 3813.

8. Wang Z, Huang G, Huang M, et al. Global patterns of phylogenetic diversity and transmission of bat coronavirus. *Sci China Life Sci*. 2023; **66**: 861-874.

9. Warmuth VM, Metzler D, Zamora-Gutierrez V. Human disturbance increases coronavirus prevalence in bats. *Sci Adv*. 2023; **9**: eadd0688.

10. Poelen JH, Simons JD, Mungall CJ. Global biotic interactions: An open infrastructure to share and analyze species-interaction datasets. *Ecol Inform* 2014; **24**: 148-59.

11. Gilardi KVK, Mazet JAK. The United States Agency for International Development Emerging Pandemic Threats PREDICT Project—Global Detection of Emerging Wildlife Viral Zoonoses. *Fowler's Zoo and Wild Animal Medicine Current Therapy*, Volume 9. 2019:110–6.

12. Choden K, Ravon S, Epstein JH, et al. *Pteropus lylei* primarily forages in residential areas in Kandal, Cambodia. *Ecol Evol* 2019; **9**: 4181–91.

13. Jones DP, Kunz TH. *Pteropus hypomelanus*. *Mamm Spec* 2000; (639): 1–6.

14. Animal Diversity Web. *Pteropus giganteus*-Indian flying fox. https://animaldiversity.org/accounts/Pteropus_giganteus/#e2087ac3806bd0afb0ea4373c083970b (accessed Dec 16 2022).

15. Kompanje EJO, Moeliker CW. Some fruit bats from remote Moluccan and West-Papuan Islands, with the description of a new subspecies of *Macroglossus minimus* (Megachiroptera: Pteropodidae). *Deinsea* 2001; **8**: 143–67.

16. Tang Z, Chen Z, Ma J, et al. Foraging areas of *Rousettus leschenaulti* on the Hainan Island of China. *Curr Zool* 2010; **56**: 479–484.

17. Elith J, Leathwick JR, Hastie T. A working guide to boosted regression trees. *J Anim Ecol* 2008; **77**: 802-13.

18. Yates LA, Aandahl Z, Richards SA, Brook BW. Cross validation for model selection: A review with examples from ecology. *Ecol Monogr* 2023; **93**: e1557.

19. Zhao GP, Wang YX, Fan ZW, et al. Mapping ticks and tick-borne pathogens in China. *Nat Commun* 2021; **12**: 1075.

20. Miao D, Dai K, Zhao GP, et al. Mapping the global potential transmission hotspots for severe fever with thrombocytopenia syndrome by machine learning methods. *Emerg Microbes Infect* 2020; **9**: 817–26.

21. Zhang YY, Sun YQ, Chen JJ, et al. Mapping the global distribution of spotted fever group rickettsiae: a systematic review with modelling analysis. *Lancet Digit Health*. 2023; **5**: e5–e15.

22. Robin X, Turck N, Hainard A, et al. pROC: an open-source package for R and S+ to analyze and compare ROC curves. *BMC bioinform* 2011; **12**: 1–8.

23. Sing T, Sander O, Beerenwinkel N, Lengauer T. ROCR: visualizing classifier performance in R. *Bioinformatics* 2005; **21**: 3940–1.

# Appendix Text S4: Demographic characteristics of patients and temporal pattern of NiV infections

**Demographic characteristics**

The demographic characteristics of the patients differed among four reporting countries. More females were reported in Bangladesh compared with other countries (40·7% vs 18·2%). Contact manner differed among countries, with all cases in Malaysia and Singapore reporting exposure to pig, 95·7% of cases in India reporting exposure to patients, while 51·3% and 16·5% of the cases in Bangladesh reported exposure to patients and contact/exposure to palms, respectively. The exposure to human patients were mainly reported in communities, except India where reports were mainly from hospitals (79·2%, 38/48).

**Temporal pattern**

A temporal clustering was observed, with outbreak events that involved 39·3% (294/749) cases were reported in Malaysia and Singapore during 1998–1999. No case was reported in 2000, 2002, 2006 and 2016, while only sporadic cases were reported in other years. The peak month with NiV case reporting differed among countries, appearing in March in Malaysia (68·6%, 194/283), January in Bangladesh (44·4%, 79/178), and February in India (63·8%, 74/116) (appendix p 63).

# Appendix Table S1: PRISMA checklist

| **Topic** | **No.** | **Item** | **Location where item is reported** |
| --- | --- | --- | --- |
| TITLE |  |  |  |
| Title | 1 | Identify the report as a systematic review. | NA |
| ABSTRACT |  |  |  |
| Abstract | 2 | See the PRISMA 2020 for Abstracts checklist | Page 2 |
| INTRODUCTION |  |  |  |
| Rationale | 3 | Describe the rationale for the review in the context of existing knowledge. | Page 5 |
| Objectives | 4 | Provide an explicit statement of the objective(s) or question(s) the review addresses. | Pages 5–6 |
| METHODS |  |  |  |
| Eligibility criteria | 5 | Specify the inclusion and exclusion criteria for the review and how studies were grouped for the syntheses. | Pages 6–7, appendix pp 22–23 |
| Information sources | 6 | Specify all databases, registers, websites, organizations, reference lists and other sources searched or consulted to identify studies. Specify the date when each source was last searched or consulted. | Page 6 |
| Search strategy | 7 | Present the full search strategies for all databases, registers and websites, including any filters and limits used. | Appendix pp 18–19 |
| Selection process | 8 | Specify the methods used to decide whether a study met the inclusion criteria of the review, including how many reviewers screened each record and each report retrieved, whether they worked independently, and if applicable, details of automation tools used in the process. | Page 6, appendix pp 7–8 |
| Data collection process | 9 | Specify the methods used to collect data from reports, including how many reviewers collected data from each report, whether they worked independently, any processes for obtaining or confirming data from study investigators, and if applicable, details of automation tools used in the process. | Page 6, appendix pp 7–8 |
| Data items | 10a | List and define all outcomes for which data were sought. Specify whether all results that were compatible with each outcome domain in each study were sought (e.g. for all measures, time points, analysis), and if not, the methods used to decide which results to collect. | Appendix pp 7–8 |
|  | 10b | List and define all other variables for which data were sought (e.g. participant and intervention characteristics, funding sources). Describe any assumptions made about any missing or unclear information. | Page 2, appendix p 24 |
| Study risk of bias assessment | 11 | Specify the methods used to assess risk of bias in the included studies, including details of the tool(s) used, how many reviewers assessed each study and whether they worked independently, and if applicable, details of automation tools used in the process. | Pages 7–8, appendix pp 7–8 |
| Effect measures | 12 | Specify for each outcome the effect measure(s) (e.g. risk ratio, mean difference) used in the synthesis or presentation of results. | Pages 7–8, appendix pp 7–8 |
| Synthesis methods | 13a | Describe the processes used to decide which studies were eligible for each synthesis (e.g. tabulating the study intervention characteristics and comparing against the planned groups for each synthesis (item 5)). | Pages 7–8, appendix pp 7–8 |
|  | 13b | Describe any methods required to prepare the data for presentation or synthesis, such as handling of missing summary statistics, or data conversions. | Appendix pp 7–8 |
|  | 13c | Describe any methods used to tabulate or visually display results of individual studies and syntheses. | Appendix pp 7–8 |
|  | 13d | Describe any methods used to synthesize results and provide a rationale for the choice(s). If meta-analysis was performed, describe the model(s), method(s) to identify the presence and extent of statistical heterogeneity, and software package(s) used. | Pages 7–8, appendix pp 7–8 |
|  | 13e | Describe any methods used to explore possible causes of heterogeneity among study results (e.g. subgroup analysis, meta-regression). | Pages 7–8, appendix pp 7–8 |
|  | 13f | Describe any sensitivity analysis conducted to assess robustness of the synthesized results. | Appendix pp 7–8 |
| Reporting bias assessment | 14 | Describe any methods used to assess risk of bias due to missing results in a synthesis (arising from reporting biases). | Pages 7–8, appendix pp 7–8 |
| Certainty assessment | 15 | Describe any methods used to assess certainty (or confidence) in the body of evidence for an outcome. | NA |
| RESULTS |  |  |  |
| Study selection | 16a | Describe the results of the search and selection process, from the number of records identified in the search to the number of studies included in the review, ideally using a flow diagram. | Pages 9 |
|  | 16b | Cite studies that might appear to meet the inclusion criteria, but which were excluded, and explain why they were excluded. | NA |
| Study characteristics | 17 | Cite each included study and present its characteristics. | Pages 9 |
| Risk of bias in studies | 18 | Present assessments of risk of bias for each included study. | Appendix p 43 |
| Results of individual studies | 19 | For all outcomes, present, for each study: (a) summary statistics for each group (where appropriate) and (b) an effect-estimate and its precision (e.g. confidence/credible interval), ideally using structured tables or plots. | Appendix pp 68–77, pp 82–89 |
| Results of syntheses | 20a | For each synthesis, briefly summarize the characteristics and risk of bias among contributing studies. | Pages 8–9 |
|  | 20b | Present results of all statistical syntheses conducted. If meta-analysis was done, present for each the summary estimate and its precision (e.g. confidence/credible interval) and measures of statistical heterogeneity. If comparing groups, describe the direction of the effect. | Page 10, appendix pp 44–45 |
|  | 20c | Present results of all investigations of possible causes of heterogeneity among study results. | Appendix pp 44–45 |
|  | 20d | Present results of all sensitivity analysis conducted to assess the robustness of the synthesized results. | Appendix pp 46–48 |
| Reporting biases | 21 | Present assessments of risk of bias due to missing results (arising from reporting biases) for each synthesis assessed. | Appendix pp 78–79, p90 |
| Certainty of evidence | 22 | Present assessments of certainty (or confidence) in the body of evidence for each outcome assessed. | NA |
| DISCUSSION |  |  |  |
| Discussion | 23a | Provide a general interpretation of the results in the context of other evidence. | Page 10–11 |
|  | 23b | Discuss any limitations of the evidence included in the review. | Page 13 |
|  | 23c | Discuss any limitations of the review processes used. | NA |
|  | 23d | Discuss implications of the results for practice, policy, and future research. | Pages 12–13 |
| OTHER INFORMATION |  |  |  |
| Registration and protocol | 24a | Provide registration information for the review, including register name and registration number, or state that the review was not registered. | Appendix pp 7–8 |
|  | 24b | Indicate where the review protocol can be accessed, or state that a protocol was not prepared. | Appendix pp 7–8 |
|  | 24c | Describe and explain any amendments to information provided at registration or in the protocol. | NA |
| Support | 25 | Describe sources of financial or non-financial support for the review, and the role of the funders or sponsors in the review. | Page 14 |
| Competing interests | 26 | Declare any competing interests of review authors. | Page 14 |
| Availability of data, code and other materials | 27 | Report which of the following are publicly available and where they can be found: template data collection forms; data extracted from included studies; data used for all analysis; analytic code; any other materials used in the review. | Appendix pp 19–21/ Appendix pp 125–130 |

**Reference**:

Page MJ, McKenzie JE, Bossuyt PM, Boutron I, Hoffmann TC, Mulrow CD, et al. The PRISMA 2020 statement: an updated guideline for reporting systematic reviews. *MetaArXiv*. 2020, September 14.

# Appendix Table S2: Literature search syntax of NiV

| **Literature database** | **Website** | **Query** |
| --- | --- | --- |
| Web of Science | http://www.webofscience.com/ | (TS= (Nipah virus)) OR (TS= (Nipah)) OR (TS= (*Henipavirus*)) OR (TS= (Hendra like virus)) |
| PubMed | https://pubmed.ncbi.nlm.nih.gov/ | (Nipah [Title/Abstract]) OR (Nipah virus [Title/Abstract]) OR (*Henipavirus* [Title/Abstract]) OR (Hendra like virus [Title/Abstract]) |
| India Journals database | https://www.indianjournals.com/ | (Nipah in Topic) OR (Nipah virus in Topic) OR (*Henipavirus* in Topic) |
| MyJurnal-Malaysia Citation Centre | https://myjurnal.mohe.gov.my/ | Nipah (Article) |
| Thai Journal Citation Index Centre | https://tci-thailand.org/ | (Nipah [Article title]) OR (*Henipavirus* [Article title]) OR (Hendra like virus [Article title]) |
| Bangladesh Journals Online | https://www.banglajol.info/ | Nipah (Article) |

# Appendix Table S3: Information of NiV infections from grey literatures

| **Institution** | **Public report or data** | **Title** | **Website** |
| --- | --- | --- | --- |
| World Health Organization (WHO) | Disease outbreak news |  |  |
|  |  | 1999 - Janpanese Encephalitis and Hendra-like Viurs in Malaysia | https://www.who.int/emergencies/disease-outbreak-news/item/1999_03_22a-en |
|  |  | 1999 - Hendra-like virus in Malaysia and Singapore | https://www.who.int/emergencies/disease-outbreak-news/item/1999_04_07a-en |
|  |  | 1999 - Hendra-like virus (Nipah virus) in Malaysia - Update | https://www.who.int/emergencies/disease-outbreak-news/item/1999_04_15-en |
|  |  | 2001 - Acute neurological syndrome in Bangladesh | https://www.who.int/emergencies/disease-outbreak-news/item/2001_05_25a-en |
|  |  | 2001 - Acute neurological syndrome in Bangladesh - Update | https://www.who.int/emergencies/disease-outbreak-news/item/2001_05_30-en |
|  |  | 2001 - Acute neurological syndrome in Bangladesh - Update 2 | https://www.who.int/emergencies/disease-outbreak-news/item/2001_06_05e-en |
|  |  | 2002- Acute neurological syndrome in Bangladesh - Update 3 | https://www.who.int/emergencies/disease-outbreak-news/item/2002_08_30a-en |
|  |  | Nipah-like virus in Bangladesh | https://www.who.int/emergencies/disease-outbreak-news/item/2004_02_12-en |
|  |  | Nipah virus - India | https://www.who.int/emergencies/disease-outbreak-news/item/31-may-2018-nipah-virus-india-en |
|  |  | Nipah virus – India | https://www.who.int/emergencies/disease-outbreak-news/item/07-august-2018-nipah-virus-india-en |
|  |  | Nipah virus disease - India | https://www.who.int/emergencies/disease-outbreak-news/item/nipah-virus-disease---india |
| Institute of Epidemiology, Disease Control and Research (IEDCR) | Outbreak investigation |  |  |
|  |  | List of Outbreak Investigations done by IEDCR in 2007 | https://iedcr.gov.bd/outbreak/c4fb4e31-5bf4-4235-b4cf-f2dc5a710dda |
|  |  | List of Outbreak investigations done by IEDCR in 2008 | https://iedcr.gov.bd/outbreak/8f0513ca-0ec5-44d5-86db-ce9da823569b |
|  |  | List of Outbreak Investigations done by IEDCR in 2010 | https://iedcr.gov.bd/outbreak/625856e3-3c82-43ac-8ce1-1265bc834e8a |
|  |  | List of Outbreak Investigations done by IEDCR in 2011 | https://iedcr.gov.bd/outbreak/d87bbd62-33c7-4db1-9aa0-d8e866e8caa0 |
|  |  | List of Outbreak Investigations done by IEDCR in 2012 | https://iedcr.gov.bd/outbreak/eb23673b-e870-4442-b401-c3a4dbac3205 |
|  |  | List of Outbreak Investigations done by IEDCR in 2013 | https://iedcr.gov.bd/outbreak/74b00638-a980-4038-9b91-5964b2db856d |
|  |  | List of Outbreak Investigations done by IEDCR in 2014 | https://iedcr.gov.bd/outbreak/8b517ac1-a581-4f3d-bc7d-b9672614225f |
|  |  | List of Outbreak Investigations done by IEDCR in 2015 | https://iedcr.gov.bd/outbreak/fadfedab-7199-453a-9447-1760505e8bbf |
| Institute of Epidemiology, Disease Control and Research (IEDCR) | Nipah Virus surveillance |  |  |
|  |  | Nipah Outbreak at Lalmonirhat | https://old.iedcr.gov.bd/website/index.php/outbreak-at-lalmonirhat |
|  |  | Nipah Outbreak at Joypurhat in January 2012 | https://old.iedcr.gov.bd/website/index.php/outbreak-at-joypurhat |
|  |  | Nipah Infection in 2014 | https://old.iedcr.gov.bd/website/pdf/files/nipah/Nipah%20Infection_27.02.2014.pdf |
|  |  | Nipah Infection in 2015 | https://old.iedcr.gov.bd/website/pdf/files/nipah/Nipah%20Infection%20in%202015_Website%20update%20Feb%204%202015_for%20website.pdf |
|  |  | Yearly distribution of Nipah cases in Bangladesh 2001-2021 | https://iedcr.gov.bd/surveillances/93c87e70-9c22-4f21-9506-a1161ecf404f |
| International Centre for Diarrhoeal Disease Research, Bangladesh (ICDDR, B) | Health Science Bulletin |  |  |
|  |  | Health and science bulletin vol. 1 no. 5 | http://dspace.icddrb.org/jspui/handle/123456789/4811 |
|  |  | Health and science bulletin vol.2 no.2 | http://dspace.icddrb.org/jspui/handle/123456789/4813 |
|  |  | Health and science bulletin vol.3 no.4 | http://dspace.icddrb.org/jspui/handle/123456789/4852 |
|  |  | Health and science bulletin vol.5 no.4 | http://dspace.icddrb.org/jspui/handle/123456789/4834 |
|  |  | Health and science bulletin vol.9 no.2 | http://dspace.icddrb.org/jspui/handle/123456789/4874 |
|  |  | Health and science bulletin vol. 10 no.1 | http://dspace.icddrb.org/jspui/handle/123456789/4879 |
| National Centre for Disease Control of India (NCDC) | Weekly Outbreak Report |  |  |
|  |  | District wise disease alerts/outbreaks reported in the 20^th^ week 2018 | https://www.idsp.nic.in/WriteReadData/l892s/202018.pdf |
|  |  | District wise disease alerts/outbreaks reported in the 21^st^ week 2018 | https://www.idsp.nic.in/WriteReadData/l892s/212018.pdf |
|  |  | District wise disease alerts/outbreaks reported in the 22^nd^ week 2018 | https://www.idsp.nic.in/WriteReadData/l892s/222018.pdf |
|  |  | District wise disease alerts/outbreaks reported in the 23^rd^ week 2018 | https://www.idsp.nic.in/WriteReadData/l892s/93428147651531734413.pdf |
|  |  | District wise disease alerts/outbreaks reported in the 22^nd^ week 2019 | https://idsp.nic.in/WriteReadData/l892s/82640712431561703101.pdf |

# Appendix Table S4: Guideline for inclusion and exclusion of literatures of NiV infections

| **Inclusion** | **Exclusion** |
| --- | --- |
| (i) Case report and surveillance of human NiV infection; | (i) Laboratory methods for NiV, including functions or animal transmission experiment of NiV; |
| (ii) Case report or surveillance of animal NiV infection; | (ii) Drug or vaccine trials without geographical or clinical information of cases; |
| (iii) Outbreak investigation of NiV infections; | (iii) Modelling or prediction of NiV, including dynamics or mathematical model of NiV infections, without background information or data source; |
| (iv) Epidemiology study of NiV infections in human or animals. | (iv) Molecular mechanism of NiV, or studies focusing on molecular or cellular level structures; |
|  | (v) Qualitative research or economic assessment of NiV infections; |
|  | (vi) Review or opinion-editorial articles of NiV; |
|  | (vii) Not related to NiV; |
|  | (viii) Duplicate literature of duplicate data that involved the same infection event |
|  | (ix) Serosurveys of health individuals; |
|  | (x) Serological study in animals; |

# Appendix Table S5: Criteria of human and animal infection with NiV

| **Case type** | **Note** | **Reference** |
| --- | --- | --- |
| **Human infection** |  |  |
| **Probable case** | A probable case is defined as the person have symptoms (e.g. fever, altered mental status) that are consistent with NiV infection during the same time and in the same community as patients with confirmed cases or had epidemiological links with confirmed human cases and from whom either serum or cerebrospinal fluid (CSF) was not available (i.e., because the patient died before specimen could be collected, or the person was detected immunoglobulin G (IgG) in a single serum/plasma specimen during the serological survey. | 1. WHO. Nipah baseline situation analysis. 2018. https://www.who.int/publications/m/item/nipah-baseline-situation-analysis.(accessed Dec 16 2022).  2. CDC. Nipah Virus: Diagnosis. 2020. https://www.cdc.gov/vhf/nipah/diagnosis/ (accessed Dec 16 2022).  3. IEDCR. National Guideline for Management, Prevention and Control of Nipah Virus Infection including Encephalitis. 2011. https://www.iedcr.org/pdf/files/nipah/National_Nipah.pdf (accessed Dec 16 2022). |
| **Confirmed case** | A confirmed case is defined as the probable case which is tested positive conducted using polymerase chain reaction (PCR) or real time polymerase chain reaction (RT-PCR) from human specimen (i.e. respiratory secretions, urine, or CSF), or tested positive of immunoglobulin M (IgM) antibody against NiV or seroconversion or >4-fold increase in specific antibody to NiV between acute and convalescent serum samples conducted using an enzyme-linked linked immunosorbent assay (ELISA) in serum or CSF, or NiV isolation positive in cell culture using brain, lung, kidney or spleen samples. |  |
| **Animal infection** |  |  |
| **Confirmed case** | Confirmation of animal cases contains the agent identification (e.g. virus isolation, PCR, RT-PCR, or immunohistochemistry). | 1. FAO. Manual on the diagnosis of Nipah virus infection in animals. 2002. https://www.fao.org/3/ac449e/ac449e.pdf (accessed Dec 16 2022).  2. OIE. Nipah and Hendra virus diseases. 2018. https://www.oie.int/fileadmin/Home/eng/Health_standards/tahm/3.01.14_NIPAH_HENDRA.pdf (accessed Dec 16 2022). |

# Appendix Table S6: Data extracted from literature

| **Variable** | **Guidance** |
| --- | --- |
| Basic information of literature |  |
| Literature ID | Unique identifier assigned to a literature. |
| Literature title | Literature title that included in the review. |
| First author | First author of the included article. |
| Publish year | Publication year of the article identified for data extraction. |
| Start year of study | Record the start year for the period over which the reported study was conducted. |
| End year of study | Record the end year for the period over which the reported study was conducted. |
| Geographic information of the study |  |
| Country | Geographic location information recorded at country-level. |
| First administrative level | Geographic location information recorded at first-level. |
| Second administrative level | Geographic location information recorded at second-level. |
| Third administrative level | Geographic location information recorded at third-level. |
| Specific location | Record the infection or detection out of third-level. |
| Laboratory information |  |
| Testing method | Record the detection testing method used, e.g. PCR, RT-PCR or ELISA. |
| Testing specimen | Record the sample tested for the diagnosis. e.g. blood, CSF. |
| Number of individuals tested all | Record the number of individuals tested for specific pathogen by testing method. |
| Number of individuals diagnosed as positive | Record the number of individuals diagnosed as positive for specific pathogen by testing method. |
| Human infections |  |
| Total cases (No) | Total number of human cases with NiV infection. |
| Death cases (No) | Total number of human due to NiV infections. |
| Male/Female ratio | The sex ratio of human cases with NiV infections. |
| Age (mean, median) | Mean or median age of human cases with NiV infections. |
| Occupation (n, %) | The number and proportion of the occupation of NiV infections. |
| Contact history (n, %) | The number and proportion of the contact history (e.g., animals, patients or palm trees). |
| Exposure site (n, %) | The number and proportion of the exposure site (e.g., community or history). |
| Clinical symptoms (n, %) | The number and proportion of human cases reporting each symptom (fever, headache, chills, cough, altered consciousness, drowsiness, seizure, nausea, vomiting, diarrhea, dyspnea, and myalgia) |
| Type of infections | Whether the cases were confirmed cases or probable cases. |
| Type of transmission | Whether the cases were zoonotic or person-to-person. |
| Animal infections |  |
| Type of animals | Whether the animals were bats or domestic animals (e.g., pig, dog, cattle or goat). |
| Family^*^ | Record the family name of bats in the taxonomy. |
| Genus^*^ | Record the genus name of bats in the taxonomy. |
| Species^*^ | Record the species name of bats in the taxonomy. |

**Note**:

^*^ The classification of taxonomy was determined by online taxonomy search websites, including Integrated Taxonomic Information System (ITIS) (https://www.itis.gov/) and National Center for Biotechnology Information (NCBI) Taxonomy (https://www.ncbi.nlm.nih.gov/taxonomy/).

# Appendix Table S7: Genome sequence used in the phylogenetic and phylogeographic analysis

| **Accession** | **Length** | **Completeness** | **Country** | **Host** | **Isolation source** | **Collection date** |
| --- | --- | --- | --- | --- | --- | --- |
| AJ627196 | 18246 | complete | Malaysia | Sus scrofa | - | 1999 |
| AJ564621 | 18246 | complete | Malaysia | Sus scrofa domesticus | lung | 1999 |
| AJ564622 | 18246 | complete | Malaysia | Sus scrofa domesticus | lung | 1999 |
| AJ564623 | 18246 | complete | Malaysia | Sus scrofa domesticus | lung | 1999 |
| AY029767 | 18246 | complete | Malaysia | Homo sapiens | cerebrospinal fluid | 1999 |
| AY029768 | 18246 | complete | Malaysia | Homo sapiens | throat secretion | 1999 |
| AF212302 | 18246 | complete | Malaysia | Homo sapiens | cerebrospinal fluid | 1999 |
| MK673558 | 18234 | complete | Malaysia | Sus scrofa domesticus | - | 1999 |
| MK673559 | 18231 | complete | Malaysia | Sus scrofa domesticus | - | 1999 |
| MK673560 | 18231 | complete | Malaysia | Sus scrofa domesticus | - | 1999 |
| MK673561 | 18231 | complete | Malaysia | Sus scrofa domesticus | - | 1999 |
| MK673562 | 18231 | complete | Malaysia | Homo sapiens | - | 1999 |
| MK673563 | 18231 | complete | Malaysia | Canis lupus familiaris | - | 1999 |
| KY425655 | 18214 | partial | Malaysia | Homo sapiens | - | 1999 |
| KY425646 | 18212 | partial | Malaysia | Homo sapiens | - | 1999 |
| AY858110 | 1599 | partial | Cambodia | *Pteropus lylei* | urine | 2000 |
| AF376747 | 11200 | partial | Malaysia | *Pteropus hypomelanus* | urine | 2000 |
| MK801755 | 18246 | complete | Cambodia | *Pteropus lylei* | - | 2003 |
| AY988601 | 18252 | complete | Bangladesh | Homo sapiens | - | 2004 |
| MK673567 | 18245 | complete | Bangladesh | Homo sapiens | oronasopharynx | 2004 |
| MK673564 | 18240 | complete | Bangladesh | Homo sapiens | oronasopharynx | 2004 |
| MK673566 | 18240 | complete | Bangladesh | Homo sapiens | urine | 2004 |
| MK673565 | 18235 | complete | Bangladesh | Homo sapiens | cerebrospinal fluid | 2004 |
| JN808858 | 1599 | partial | Bangladesh | Homo sapiens | oronasopharynx | 2004 |
| JN808861 | 1599 | partial | Bangladesh | Homo sapiens | oronasopharynx | 2004 |
| JN808862 | 1599 | partial | Bangladesh | Homo sapiens | oronasopharynx | 2004 |
| FJ513078 | 18252 | complete | India | Homo sapiens | lung | 2007 |
| JN808857 | 18252 | complete | Bangladesh | Homo sapiens | oronasopharynx | 2008 |
| JN808863 | 18252 | complete | Bangladesh | Homo sapiens | oronasopharynx | 2008 |
| MK673568 | 18237 | complete | Bangladesh | Homo sapiens | oronasopharynx | 2008 |
| FN869553 | 14973 | partial | Malaysia | *Pteropus vampyrus* | urine | 2008 |
| JN808864 | 18167 | partial | Bangladesh | Homo sapiens | oronasopharynx | 2010 |
| JN808859 | 1599 | partial | Bangladesh | Homo sapiens | oronasopharynx | 2010 |
| JN808860 | 1599 | partial | Bangladesh | Homo sapiens | oronasopharynx | 2010 |
| KT163251 | 1599 | partial | Thailand | *Pteropus lylei* | urine | 2010 |
| KT163249 | 1599 | partial | Thailand | *Pteropus hypomelanus* | urine | 2010 |
| KT163255 | 1599 | partial | Thailand | *Pteropus lylei* | urine | 2010 |
| KT163253 | 1599 | partial | Thailand | *Pteropus lylei* | urine | 2010 |
| KT163254 | 1599 | partial | Thailand | *Pteropus lylei* | urine | 2010 |
| KT163247 | 1599 | partial | Thailand | *Pteropus hypomelanus* | urine | 2010 |
| MK673571 | 18240 | complete | Bangladesh | Homo sapiens | oronasopharynx | 2011 |
| MK673575 | 18238 | complete | Bangladesh | Homo sapiens | oronasopharynx | 2011 |
| MK673576 | 18237 | complete | Bangladesh | Homo sapiens | oronasopharynx | 2011 |
| MK673573 | 18236 | complete | Bangladesh | Homo sapiens | oronasopharynx | 2011 |
| MK673578 | 18235 | complete | Bangladesh | Homo sapiens | blood | 2011 |
| MK673570 | 18231 | complete | Bangladesh | Homo sapiens | oronasopharynx | 2011 |
| MK673577 | 18219 | complete | Bangladesh | Homo sapiens | blood | 2011 |
| MK673574 | 18205 | complete | Bangladesh | Homo sapiens | oronasopharynx | 2011 |
| MK673572 | 18105 | partial | Bangladesh | Homo sapiens | oronasopharynx | 2011 |
| KT163248 | 1599 | partial | Thailand | *Pteropus hypomelanus* | urine | 2011 |
| KT163252 | 1599 | partial | Thailand | *Pteropus lylei* | urine | 2011 |
| KT163256 | 1599 | partial | Thailand | *Pteropus lylei* | urine | 2011 |
| KT163250 | 1599 | partial | Thailand | *Pteropus hypomelanus* | urine | 2011 |
| MK673582 | 18237 | complete | Bangladesh | Homo sapiens | oronasopharynx | 2012 |
| MK673581 | 18235 | complete | Bangladesh | Homo sapiens | oronasopharynx | 2012 |
| MK673580 | 18234 | complete | Bangladesh | Homo sapiens | oronasopharynx | 2012 |
| MK673579 | 18223 | complete | Bangladesh | Homo sapiens | oronasopharynx | 2012 |
| MT890702 | 1599 | partial | Bangladesh | Chiroptera | urine | 2012 |
| MK575060 | 18234 | complete | Bangladesh | *Pteropus medius* | urine | 2013 |
| MK575061 | 18234 | complete | Bangladesh | *Pteropus medius* | urine | 2013 |
| MK575062 | 18234 | complete | Bangladesh | *Pteropus medius* | urine | 2013 |
| MK575064 | 18234 | complete | Bangladesh | *Pteropus medius* | urine | 2013 |
| MK575065 | 18234 | complete | Bangladesh | *Pteropus medius* | urine | 2013 |
| MK575066 | 18234 | complete | Bangladesh | *Pteropus medius* | urine | 2013 |
| MK575067 | 18234 | complete | Bangladesh | *Pteropus medius* | urine | 2013 |
| MK575068 | 18234 | complete | Bangladesh | *Pteropus medius* | urine | 2013 |
| MK575069 | 18234 | complete | Bangladesh | *Pteropus medius* | urine | 2013 |
| MK575070 | 18234 | complete | Bangladesh | *Pteropus medius* | urine | 2013 |
| MK673583 | 18197 | complete | Bangladesh | Homo sapiens | oronasopharynx | 2013 |
| MT890728 | 1599 | partial | Bangladesh | Homo sapiens | - | 2013 |
| MT890729 | 1599 | partial | Bangladesh | Homo sapiens | - | 2013 |
| MT890713 | 1599 | partial | Bangladesh | Homo sapiens | - | 2013 |
| MT890714 | 1599 | partial | Bangladesh | Homo sapiens | - | 2013 |
| MT890715 | 1599 | partial | Bangladesh | Homo sapiens | - | 2013 |
| MT890712 | 1599 | partial | Bangladesh | Homo sapiens | - | 2013 |
| KM034755 | 1599 | partial | Cambodia | Pteropus lylei | - | 2013 |
| MK673592 | 18234 | complete | Bangladesh | Homo sapiens | oronasopharynx | 2014 |
| MK673589 | 18215 | complete | Bangladesh | Homo sapiens | oronasopharynx | 2014 |
| MK673590 | 18208 | complete | Bangladesh | Homo sapiens | oronasopharynx | 2014 |
| MK673591 | 18157 | complete | Bangladesh | Homo sapiens | oronasopharynx | 2014 |
| MT890717 | 1592 | partial | Bangladesh | Homo sapiens | - | 2014 |
| MK673585 | 18245 | complete | Bangladesh | Homo sapiens | oronasopharynx | 2015 |
| MK673584 | 18229 | complete | Bangladesh | Homo sapiens | oronasopharynx | 2015 |
| MK673587 | 18170 | complete | Bangladesh | Homo sapiens | oronasopharynx | 2015 |
| MK673586 | 18165 | complete | Bangladesh | Homo sapiens | oronasopharynx | 2015 |
| MK673588 | 18119 | partial | Bangladesh | Homo sapiens | oronasopharynx | 2015 |
| MT890723 | 1599 | partial | Bangladesh | Homo sapiens | - | 2015 |
| MW535746 | 18236 | complete | Thailand | *Pteropus lylei* | urine | 2017 |
| MT890730 | 1599 | partial | Bangladesh | Homo sapiens | - | 2018 |
| MH523642 | 18242 | complete | India | Homo sapiens | oronasopharynx | 2018 |
| MK336156 | 18233 | partial | India | Homo sapiens | oronasopharynx | 2018 |
| MK336155 | 18211 | partial | India | Homo sapiens | oronasopharynx | 2018 |
| MH396625 | 18210 | complete | India | Homo sapiens | oronasopharynx | 2018 |
| MH523640 | 18132 | complete | India | Homo sapiens | lung | 2018 |
| MH523641 | 18027 | complete | India | Homo sapiens | oronasopharynx | 2018 |
| MH891773 | 1611 | partial | India | Homo sapiens | oronasopharynx | 2018 |
| MN549409 | 18172 | complete | India | *Pteropus medius* | - | 2019 |
| MN549411 | 18124 | partial | India | *Pteropus medius* | lung | 2019 |
| OM135495 | 18005 | partial | India | Homo sapiens | - | 2021 |

# Appendix Table S8: Source of data used in the modelling analysis

| **Variables** | **Dataset** | **Temporal extent** | **Spatial resolution** | **Website** | **Reference** |
| --- | --- | --- | --- | --- | --- |
| Climate data | WorldClim | 1980-2018* | 0° 2.5′ | https://www.worldclim.org/ | Fick, S.E. and R.J. Hijmans. WorldClim 2: new 1km spatial resolution climate surfaces for global land areas. International Journal of Climatology 2017; **37**: 4302-4315.  Harris, I., P.D. Jones, T.J. Osborn, et al. Updated high-resolution grids of monthly climatic observations - the CRU TS3.10 Dataset. International Journal of Climatology 2014; **34**: 623-642. |
| Elevation | EarthEnv-DEM90 digital elevation model | 2010 | 1 km | http://www.earthenv.org/ | Robinson, N., Regetz, J., Guralnick, R. P.. EarthEnv-DEM90: A nearly-global, void-free, multi-scale smoothed, 90m digital elevation model from fused ASTER and SRTM data. ISPRS Journal of Photogrammetry and Remote Sensing 2014; **87**: 57-67. |
| Leaf area index | GLOBMAP Leaf Area Index (LAI) Version 3 | 1981-2019 | 8 km | https://www.resdc.cn/ | Liu, Y., R. Liu, and J. M. Chen. Retrospective retrieval of long-term consistent global leaf area index (1981–2011) from combined AVHRR and MODIS data, J. Geophys. Res 2012; **117**: G04003. |
| Land cover | The Land Cover CCI Climate Research Data Package (CRDP) | 1992-2019 | 0.3 km | https://maps.elie.ucl.ac.be/CCI/ | ESA Land Cover CCI project team; Defourny, P. (2019): ESA Land Cover Climate Change Initiative (Land_Cover_cci): Global Land Cover Maps, Version 2.0.7. Centre for Environmental Data Analysis. |
| Pig density | Livestock Production | 2015 | 1 km | https://livestock.geo-wiki.org/ | Gilbert M, Conchedda G, Van Boeckel TP, et al. Income Disparities and the Global Distribution of Intensively Farmed Chicken and Pigs. *PLoS One*. 2015; **10**: e0133381. |
| Mammalian richness | Maps of mammal diversity | 2018 | 10 km | https://biodiversitymapping.org/ | Jenkins CN, Pimm SL, Joppa LN. Global patterns of terrestrial vertebrate diversity and conservation. Proc Natl Acad Sci U S A. 2013; **110**: E2602-E2610. |
| Gridded Population density | Population counts / Unconstrained global mosaics 2000-2020 (1km resolution) | 2000-2020 | 1 km | https://www.worldpop.org/ | WorldPop (www.worldpop.org - School of Geography and Environmental Science, University of Southampton; Department of Geography and Geosciences, University of Louisville; Departement de Geographie, Universite de Namur) and Center for International Earth Science Information Network (CIESIN), Columbia University (2018). Global High-Resolution Population Denominators Project - Funded by The Bill and Melinda Gates Foundation (OPP1134076). https://dx.doi.org/10.5258/SOTON/WP00647 |
| Global Downscaled GDP | Global 15 x 15 Minute Grids of the Downscaled GDP Based on the SRES B2 Scenario, v1 (1990, 2025) | 1990, 2025 | 0° 15′ | https://sedac.ciesin.columbia.edu/ | Gaffin SR, Xing X, Yetman G. Global 15 x 15 Minute Grids of the Downscaled GDP Based on the SRES B2 Scenario, 1990 and 2025. Palisades, NY: NASA Socioeconomic Data and Applications Center (SEDAC); 2004. |
| Human Footprint | Human Footprint dataset from 2000 to 2018 | 2000-2018 | 1 km | https://doi.org/10.6084/m9.figshare.16571064 | Mu H, Li X, Wen Y, et al. A global record of annual terrestrial Human Footprint dataset from 2000 to 2018. *Sci Data* 2022; **9**: 176. |

**Note**: ^*^This dataset is recalculated according to historical monthly weather data for 1980-2018, by using the ‘biovars’ function in the R package “dismo”.

# Appendix Table S9: Description of 50 potential predictive variables used in the modelling

| **Group** | **Variable** | **Description (all used as continuous variable)** |
| --- | --- | --- |
| Environment |  |  |
|  | BIO1 | Annual mean temperature (℃) |
|  | BIO2 | Mean diurnal range (Mean of monthly (max temp-min temp)) (℃) |
|  | BIO3 | Isothermality (BIO2/ BIO7) (*100) |
|  | BIO4 | Temperature seasonality (standard deviation*100) |
|  | BIO5 | Max temperature of warmest month (℃) |
|  | BIO6 | Min temperature of coldest month (℃) |
|  | BIO7 | Annual range of temperature (BIO5- BIO6) (℃) |
|  | BIO8 | Mean temperature of wettest quarter (℃) |
|  | BIO9 | Mean temperature of driest quarter (℃) |
|  | BIO10 | Mean temperature of warmest quarter (℃) |
|  | BIO11 | Mean temperature of coldest quarter (℃) |
|  | BIO12 | Annual precipitation (mm) |
|  | BIO13 | Precipitation of wettest month (mm) |
|  | BIO14 | Precipitation of driest month (mm) |
|  | BIO15 | Precipitation seasonality (Coefficient of variation) |
|  | BIO16 | Precipitation of wettest quarter (mm) |
|  | BIO17 | Precipitation of driest quarter (mm) |
|  | BIO18 | Precipitation of warmest quarter (mm) |
|  | BIO19 | Precipitation of coldest quarter (mm) |
|  | Leaf area index | Area of leaves (m²) over a unit of land (m²) |
|  | Elevation | Average elevation (m) |
|  | Mixed cropland and nature vegetation | Percentage coverage of mixed cropland and nature vegetation (1%) |
|  | Forest | Percentage coverage of forest (1%) |
|  | Shrubland | Percentage coverage of shrubland (1%) |
|  | Mixed tree, shrub and herbaceous | Percentage coverage of mixed tree, shrub and herbaceous (1%) |
|  | Grassland | Percentage coverage of grassland (1%) |
|  | Sparse vegetation land | Percentage coverage of sparse vegetation land (1%) |
|  | Flooded vegetation | Percentage coverage of flooded vegetation (1%) |
|  | Bare areas | Percentage coverage of bare areas (1%) |
|  | Water body | Percentage coverage of inland water body (1%) |
|  | Date palms (*Phoenix sylvestris*) | Probability of *Phoenix sylvestris* occurrence |
| Human activity |  |  |
|  | Cropland | Percentage coverage of cropland (1%) |
|  | Cropland change (calculated) | Change in the proportion of cropland (1%) |
|  | Urban construction land | Percentage coverage of urban construction land (1%) |
|  | Urban change (calculated) | Change in the proportion of urban construction land (1%) |
|  | Gridded population density | Estimated total number of people per grid-cell |
|  | Gridded population change (calculated) | Population change from year 2000 to 2020 per grid-cell |
|  | Global Downscaled GDP | Geospatial distributions of GDP per unit area |
|  | GDP change (calculated) | Change in GDP per grid-cell |
|  | Human footprint | Annual records of the global human footprint (0-50) |
| Animal |  |  |
|  | Mammalian richness | The number of mammal species per grid-cell |
|  | Pig density with intensive production | Pig density with intensive production (heads per grid-cell) |
|  | Chiroptera richness | The number of Chiroptera species per grid-cell |
|  | *Pteropus lylei* | Probability of *Pteropus lylei* occurrence |
|  | *Pteropus medius* | Probability of *Pteropus medius* occurrence |
|  | *Pteropus vampyrus* | Probability of *Pteropus vampyrus* occurrence |
|  | *Pteropus hypomelanus* | Probability of *Pteropus hypomelanus* occurrence |
|  | *Rousettus leschenaultii* | Probability of *Rousettus leschenaultii* occurrence |
|  | *Rousettus amplexicaudatus* | Probability of *Rousettus amplexicaudatus* occurrence |
|  | *Hipposideros larvatus* | Probability of *Hipposideros larvatus* occurrence |

# Appendix Table S10: Ecological factors potentially associated with the bat and bat-borne pathogens used in the modelling analysis.

| **Variable** | **Reference** | **Content** | **Method** | **Usage and/or result** |
| --- | --- | --- | --- | --- |
| BIO1 | McKee CD, et al.^1^ | The ecology of NiV in Bangladesh | Model: GLM and RF | Used as the spatial covariate in the occupancy and abundance analysis of NiV reservoir hosts (*Pteropus medius*) |
|  | Daszak P, et al.^2^ | The future potential distribution of emerging *Henipavirus* reservoirs | Model: MaxEnt | Used in the ecological niche model of *Henipavirus* reservoirs |
|  | Walsh MG.^3^ | The risk of NiV spillover events in South and Southeast Asia | Model: MaxEnt and inhomogeneous Poisson point process model. | Used in the ecological niche model of NiV reservoir hosts |
|  | Peterson AT.^4^ | The risk of NiV transmission across Asia and across Bangladesh | Model: MaxEnt and GARP | Used in the ecological niche model |
|  | Nyakarahuka L, et al.^5^ | The zoonotic niche of Ebola and Marburg virus in Uganda | Model: MaxEnt | Used in the ecological niche model of Ebola and Marburg virus |
|  | Muylaert RL, et al.^6^ | The ecological niche sarbecoviruses | Model: MaxEnt | Used in the ecological niche sarbecoviruses |
|  | Zhang F, et al.^7^ | The discovery of human-infective RNA viruses | Model: BRT | Used in the ecological niche model |
| BIO2 | McKee CD, et al.^1^ | The ecology of NiV in Bangladesh | Model: GLM and RF | Used as the spatial covariate in the occupancy and abundance analysis of NiV reservoir hosts (*Pteropus medius*) |
|  | Daszak P, et al.^2^ | The future potential distribution of emerging *Henipavirus* reservoirs | Model: MaxEnt | Used in the ecological niche model of *Henipavirus* reservoirs |
|  | Peterson AT.^4^ | The risk of NiV transmission across Asia and across Bangladesh | Model: MaxEnt and GARP | Used in the ecological niche model |
|  | Nyakarahuka L, et al.^5^ | The zoonotic niche of Ebola and Marburg virus in Uganda | Model: MaxEnt | Used in the ecological niche model of Ebola and Marburg virus |
|  | Zhang F, et al.^7^ | The discovery of human-infective RNA viruses | Model: BRT | Used in the ecological niche model |
|  | Hassanin A, et al.^8^ | The ecological niche of *Rhinolophus* species | Model: MaxEnt | Used in the ecological niche of *Rhinolophus* species |
|  | Han Y, et al.^9^ | The bat ecological distributions in Southern China | Model: MaxEnt | Used in the ecological niche of bat |
|  | Brierley L, et al.^10^ | The drivers of zoonotic bat viruses | Model: Conditional autoregressive | Used in the spatially explicit linear model |
| BIO3 | Daszak P, et al.^2^ | The future potential distribution of emerging *Henipavirus* reservoirs | Model: MaxEnt | Used in the ecological niche model of *Henipavirus* reservoirs |
|  | Peterson AT.^4^ | The risk of NiV transmission across Asia and across Bangladesh | Model: MaxEnt and GARP | Used in the ecological niche model |
|  | Nyakarahuka L, et al.^5^ | The zoonotic niche of Ebola and Marburg virus in Uganda | Model: MaxEnt | Used in the ecological niche model of Ebola and Marburg virus |
|  | Hassanin A, et al.^8^ | The ecological niche of *Rhinolophus* species | Model: MaxEnt | Used in the ecological niche of *Rhinolophus* species |
|  | Han Y, et al.^9^ | The bat ecological distributions in Southern China | Model: MaxEnt | Used in the ecological niche of bat |
| BIO4 | Daszak P, et al.^2^ | The future potential distribution of emerging *Henipavirus* reservoirs | Model: MaxEnt | Used in the ecological niche model of *Henipavirus* reservoirs |
|  | Peterson AT.^4^ | The risk of NiV transmission across Asia and across Bangladesh | Model: MaxEnt and GARP | Used in the ecological niche model |
|  | Nyakarahuka L, et al.^5^ | The zoonotic niche of Ebola and Marburg virus in Uganda | Model: MaxEnt | Used in the ecological niche model of Ebola and Marburg virus |
|  | Muylaert RL, et al.^6^ | The ecological niche sarbecoviruses | Model: MaxEnt | Used in the ecological niche sarbecoviruses |
|  | Han Y, et al.^9^ | The bat ecological distributions in Southern China | Model: MaxEnt | Used in the ecological niche of bat |
| BIO5 | McKee CD, et al.^1^ | The ecology of NiV in Bangladesh | Model: GLM and RF | Used as the spatial covariate in the occupancy and abundance analysis of NiV reservoir hosts (*Pteropus medius*) |
|  | Daszak P, et al.^2^ | The future potential distribution of emerging *Henipavirus* reservoirs | Model: MaxEnt | Used in the ecological niche model of *Henipavirus* reservoirs |
|  | Peterson AT.^4^ | The risk of NiV transmission across Asia and across Bangladesh | Model: MaxEnt and GARP | Used in the ecological niche model |
|  | Zhang F, et al.^7^ | The discovery of human-infective RNA viruses | Model: BRT | Used in the ecological niche model |
| BIO6 | McKee CD, et al.^1^ | The ecology of NiV in Bangladesh | Model: GLM and RF | Used as the spatial covariate in the occupancy and abundance analysis of NiV reservoir hosts (*Pteropus medius*) |
|  | Daszak P, et al.^2^ | The future potential distribution of emerging *Henipavirus* reservoirs | Model: MaxEnt | Used in the ecological niche of *Henipavirus* reservoirs |
|  | Peterson AT.^4^ | The risk of NiV transmission across Asia and across Bangladesh | Model: MaxEnt and GARP | Used in the ecological niche model |
|  | Zhang F, et al.^7^ | The discovery of human-infective RNA viruses | Model: BRT | Used in the ecological niche model |
| BIO7 | McKee CD, et al.^1^ | The future potential distribution of emerging *Henipavirus* reservoirs | Model: MaxEnt | Used in the ecological niche model of *Henipavirus* reservoirs |
|  | Peterson AT.^4^ | The risk of NiV transmission across Asia and across Bangladesh | Model: MaxEnt and GARP | Used in the ecological niche model |
|  | Zhang F, et al.^7^ | The discovery of human-infective RNA viruses | Model: BRT | Used in the ecological niche model |
|  | Hassanin A, et al.^8^ | The ecological niche of *Rhinolophus* species | Model: MaxEnt | Used in the ecological niche of *Rhinolophus* species |
|  | Brierley L, et al.^10^ | The drivers of zoonotic bat viruses | Model: Conditional autoregressive | Used in the spatially explicit linear model |
| BIO8 | Daszak P, et al.^2^ | The future potential distribution of emerging *Henipavirus* reservoirs | Model: MaxEnt | Used in the ecological niche model of *Henipavirus* reservoirs |
|  | Peterson AT.^4^ | The risk of NiV transmission across Asia and across Bangladesh | Model: MaxEnt and GARP | Used in the ecological niche model |
| BIO9 | Daszak P, et al.^2^ | The future potential distribution of emerging *Henipavirus* reservoirs | Model: MaxEnt | Used in the ecological niche model of *Henipavirus* reservoirs |
|  | Peterson AT.^4^ | The risk of NiV transmission across Asia and across Bangladesh | Model: MaxEnt and GARP | Used in the ecological niche model |
| BIO10 | Daszak P, et al.^2^ | The future potential distribution of emerging *Henipavirus* reservoirs | Model: MaxEnt | Used in the ecological niche model of *Henipavirus* reservoirs |
|  | Peterson AT.^4^ | The risk of NiV transmission across Asia and across Bangladesh | Model: MaxEnt and GARP | Used in the ecological niche model |
|  | Hassanin A, et al.^8^ | The ecological niche of *Rhinolophus* species | Model: MaxEnt | Used in the ecological niche of *Rhinolophus* species |
|  | Han Y, et al.^9^ | The bat ecological distributions in Southern China | Model: MaxEnt | Used in the ecological niche of bat |
| BIO11 | Daszak P, et al.^2^ | The future potential distribution of emerging *Henipavirus* reservoirs | Model: MaxEnt | Used in the ecological niche model of *Henipavirus* reservoirs |
|  | Peterson AT.^4^ | The risk of NiV transmission across Asia and across Bangladesh | Model: MaxEnt and GARP | Used in the ecological niche model |
|  | Han Y, et al.^9^ | The bat ecological distributions in Southern China | Model: MaxEnt | Used in the ecological niche of bat |
| BIO12 | McKee CD, et al.^1^ | The ecology of NiV in Bangladesh | Model: GLM and RF | Used as the spatial covariate in the occupancy and abundance analysis of NiV reservoir hosts (*Pteropus medius*) |
|  | Daszak P, et al.^2^ | The future potential distribution of emerging *Henipavirus* reservoirs | Model: MaxEnt | Used in the ecological niche model of *Henipavirus* reservoirs |
|  | Walsh MG.^3^ | The risk of NiV spillover events in South and Southeast Asia | Model: MaxEnt and inhomogeneous Poisson point process model. | Used in the ecological niche model of NiV reservoir hosts |
|  | Peterson AT.^4^ | The risk of NiV transmission across Asia and across Bangladesh | Model: MaxEnt and GARP | Used in the ecological niche model |
|  | Nyakarahuka L, et al.^5^ | The zoonotic niche of Ebola and Marburg virus in Uganda | Model: MaxEnt | Used in the ecological niche model of Ebola and Marburg virus |
|  | Muylaert RL, et al.^6^ | The ecological niche sarbecoviruses | Model: MaxEnt | Used in the ecological niche sarbecoviruses |
|  | Zhang F, et al.^7^ | The discovery of human-infective RNA viruses | Model: BRT | Used in the ecological niche model |
|  | Han Y, et al.^9^ | The bat ecological distributions in Southern China | Model: MaxEnt | Used in the ecological niche of bat |
| BIO13 | Daszak P, et al.^2^ | The future potential distribution of emerging *Henipavirus* reservoirs | Model: MaxEnt | Used in the ecological niche model of *Henipavirus* reservoirs |
|  | Peterson AT.^4^ | The risk of NiV transmission across Asia and across Bangladesh | Model: MaxEnt and GARP | Used in the ecological niche model |
|  | Zhang F, et al.^7^ | The discovery of human-infective RNA viruses | Model: BRT | Used in the ecological niche model |
|  | Hassanin A, et al.^8^ | The ecological niche of *Rhinolophus* species | Model: MaxEnt | Used in the ecological niche of *Rhinolophus* species |
| BIO14 | McKee CD, et al.^1^ | The ecology of NiV in Bangladesh | Model: GLM and RF | Used as the spatial covariate in the occupancy and abundance analysis of NiV reservoir hosts (*Pteropus medius*) |
|  | Daszak P, et al.^2^ | The future potential distribution of emerging *Henipavirus* reservoirs | Model: MaxEnt | Used in the ecological niche model of *Henipavirus* reservoirs |
|  | Peterson AT.^4^ | The risk of NiV transmission across Asia and across Bangladesh | Model: MaxEnt and GARP | Used in the ecological niche model |
|  | Zhang F, et al.^7^ | The discovery of human-infective RNA viruses | Model: BRT | Used in the ecological niche model |
|  | Han Y, et al.^9^ | The bat ecological distributions in Southern China | Model: MaxEnt | Used in the ecological niche of bat |
| BIO15 | McKee CD, et al.^1^ | The ecology of NiV in Bangladesh | Model: GLM and RF | Used as the spatial covariate in the occupancy and abundance analysis of NiV reservoir hosts (*Pteropus medius*) |
|  | Daszak P, et al.^2^ | The future potential distribution of emerging *Henipavirus* reservoirs | Model: MaxEnt | Used in the ecological niche model of *Henipavirus* reservoirs |
|  | Peterson AT.^4^ | The risk of NiV transmission across Asia and across Bangladesh | Model: MaxEnt and GARP | Used in the ecological niche model |
|  | Nyakarahuka L, et al.^5^ | The zoonotic niche of Ebola and Marburg virus in Uganda | Model: MaxEnt | Used in the ecological niche model of Ebola and Marburg virus |
|  | Muylaert RL, et al.^6^ | The ecological niche sarbecoviruses | Model: MaxEnt | Used in the ecological niche sarbecoviruses |
|  | Hassanin A, et al.^8^ | The ecological niche of *Rhinolophus* species | Model: MaxEnt | Used in the ecological niche of *Rhinolophus* species |
|  | Han Y, et al.^9^ | The bat ecological distributions in Southern China | Model: MaxEnt | Used in the ecological niche of bat |
| BIO16 | Daszak P, et al.^2^ | The future potential distribution of emerging *Henipavirus* reservoirs | Model: MaxEnt | Used in the ecological niche model of *Henipavirus* reservoirs |
|  | Peterson AT.^4^ | The risk of NiV transmission across Asia and across Bangladesh | Model: MaxEnt and GARP | Used in the ecological niche model |
| BIO17 | Daszak P, et al.^2^ | The future potential distribution of emerging *Henipavirus* reservoirs | Model: MaxEnt | Used in the ecological niche model of *Henipavirus* reservoirs |
|  | Peterson AT.^4^ | The risk of NiV transmission across Asia and across Bangladesh | Model: MaxEnt and GARP | Used in the ecological niche model |
|  | Nyakarahuka L, et al.^5^ | The zoonotic niche of Ebola and Marburg virus in Uganda | Model: MaxEnt | Used in the ecological niche model of Ebola and Marburg virus |
|  | Hassanin A, et al.^8^ | The ecological niche of *Rhinolophus* species | Model: MaxEnt | Used in the ecological niche of *Rhinolophus* species |
| BIO18 | McKee CD, et al.^1^ | The ecology of NiV in Bangladesh | Model: GLM and RF | Used as the spatial covariate in the occupancy and abundance analysis of NiV reservoir hosts (*Pteropus medius*) |
|  | Daszak P, et al.^2^ | The future potential distribution of emerging *Henipavirus* reservoirs | Model: MaxEnt | Used in the ecological niche model of *Henipavirus* reservoirs |
|  | Peterson AT.^4^ | The risk of NiV transmission across Asia and across Bangladesh | Model: MaxEnt and GARP | Used in the ecological niche model |
|  | Hassanin A, et al.^8^ | The ecological niche of *Rhinolophus* species | Model: MaxEnt | Used in the ecological niche of *Rhinolophus* species |
| BIO19 | Daszak P, et al.^2^ | The future potential distribution of emerging *Henipavirus* reservoirs | Model: MaxEnt | Used in the ecological niche model of *Henipavirus* reservoirs |
|  | Peterson AT.^4^ | The risk of NiV transmission across Asia and across Bangladesh | Model: MaxEnt and GARP | Used in the ecological niche model |
| Leaf area index | Walsh MG.^3^ | The risk of NiV spillover events in South and Southeast Asia | Model: MaxEnt and inhomogeneous Poisson point process model. | Green vegetation cover was used in the spatially explicit analysis of NiV spillover events |
|  | Pigott DM, et al.^11^ | The zoonotic niche of Ebola virus disease in Africa | Model: BRT | Enhanced Vegetation Index was used in the zoonotic niche of Ebola |
|  | Escobar LE, et al.^12^ | Predicting the risk of bat-borne rabies in Chile | Model: MaxEnt | Normalized Difference Vegetation Index was used in the the ecological niche of bat-borne rabies |
| Elevation | McKee CD, et al.^1^ | The ecology of NiV in Bangladesh | Model: GLM and RF | Used as the spatial covariate in the occupancy and abundance analysis of NiV reservoir hosts (*Pteropus medius*) |
|  | Deka MA, et al.^13^ | The distribution of NiV transmission risk in South and Southeast Asia | Medel: MaxEnt | Used in the NiV ecological niche model |
|  | Pigott DM, et al.^11^ | The zoonotic niche of Ebola virus disease in Africa | Model: BRT | Used in the Ebola niche model |
| Mixed cropland and nature vegetation | Zhang F, et al.^7^ | The discovery of human-infective RNA viruses | Model: BRT | Used in the ecological niche model |
|  | Deka MA, et al.^13^ | The distribution of NiV transmission risk in South and Southeast Asia | Model: MaxEnt | Land cover data was used in the NiV ecological niche model |
| Forest | Deka MA, et al.^13^ | The distribution of NiV transmission risk in South and Southeast Asia | Model: MaxEnt | Land cover data was used in the NiV ecological niche model |
| Shrubland | Deka MA, et al.^13^ | The distribution of NiV transmission risk in South and Southeast Asia | Model: MaxEnt | Land cover data was used in the NiV ecological niche model |
| Mixed tree, shrub and herbaceous | Deka MA, et al.^13^ | The distribution of NiV transmission risk in South and Southeast Asia | Model: MaxEnt | Land cover data was used in the NiV ecological niche model |
| Grassland | Deka MA, et al.^13^ | The distribution of NiV transmission risk in South and Southeast Asia | Model: MaxEnt | Land cover data was used in the NiV ecological niche model |
| Sparse vegetation land | Deka MA, et al.^13^ | The distribution of NiV transmission risk in South and Southeast Asia | Medel: MaxEnt | Land cover data was used in the NiV ecological niche model |
| Flooded vegetation | Deka MA, et al.^13^ | The distribution of NiV transmission risk in South and Southeast Asia | Medel: MaxEnt | Land cover data was used in the NiV ecological niche model |
| Bare areas | Deka MA, et al.^13^ | The distribution of NiV transmission risk in South and Southeast Asia | Model: MaxEnt | Land cover data was used in the NiV ecological niche model |
| Water body | Deka MA, et al.^13^ | The distribution of NiV transmission risk in South and Southeast Asia | Model: MaxEnt | Land cover data was used in the NiV ecological niche model |
| Cropland  Cropland change (calculated) | Zhang F, et al.^7^ | The discovery of human-infective RNA viruses | Model: BRT | Used in the ecological niche model |
|  | Brierley L, et al.^10^ | The drivers of zoonotic bat viruses | Model: Conditional autoregressive | Cropland was used in the spatially explicit linear model |
|  | Deka MA, et al.^13^ | The distribution of NiV transmission risk in South and Southeast Asia | Model: MaxEnt | Land cover data was used in the NiV ecological niche model |
| Urban construction land  Urban change (calculated) | Zhang F, et al.^7^ | The discovery of human-infective RNA viruses | Model: BRT | Used in the ecological niche model |
|  | Deka MA, et al.^13^ | The distribution of NiV transmission risk in South and Southeast Asia | Model: MaxEnt | Land cover data was used in the NiV ecological niche model |
| Gridded population density  Gridded population change (calculated) | McKee CD, et al.^1^ | The ecology of NiV in Bangladesh | Model: GLM and RF | Used as the spatial covariate in the occupancy and abundance analysis of Nipah reservoir hosts (*Pteropus medius*) |
|  | Zhang F, et al.^7^ | The discovery of human-infective RNA viruses | Model: BRT | Used in the ecological niche model |
|  | Brierley L, et al.^10^ | The drivers of zoonotic bat viruses | Model: Conditional autoregressive | Used in the spatially explicit linear model |
| Global Downscaled GDP  GDP change (calculated) | Zhang F, et al.^7^ | The discovery of human-infective RNA viruses | Model: BRT | GDP was used in the ecological niche model |
| Human footprint | Walsh MG.^3^ | The risk of NiV spillover events in South and Southeast Asia | Model: MaxEnt and inhomogeneous Poisson point process model. | Used in the spatially explicit analysis of NiV spillover events |
| Date palms (*Phoenix sylvestris*) | McKee CD, et al.^1^ | The ecology of NiV in Bangladesh | Model: GLM and RF | The location and number of *Phoenix sylvestris* were used in the occupancy and abundance analysis of Nipah reservoir hosts (*Pteropus medius*). |
| Mammalian richness | Zhang F, et al.^7^ | The discovery of human-infective RNA viruses | Model: BRT | Used in the ecological niche model |
| Pig density with intensive production | Walsh MG.^3^ | The risk of NiV spillover events in South and Southeast Asia | Model: MaxEnt and inhomogeneous Poisson point process model. | Pig distribution was used in the spatially explicit analysis of NiV spillover events |
|  | Brierley L, et al.^10^ | The drivers of zoonotic bat viruses | Model: Conditional autoregressive | Pig distribution was used in the spatially explicit linear model |
|  | Deka MA, et al.^13^ | The distribution of NiV transmission risk in South and Southeast Asia | Model: MaxEnt | Pig distribution was used in the NiV ecological niche model |
| Chiroptera richness | Brierley L, et al.^10^ | The drivers of zoonotic bat viruses | Model: Conditional autoregressive | Used in the spatially explicit linear model |
| *Pteropus lylei*  *Pteropus medius*  *Pteropus vampyrus*  *Pteropus hypomelanus*  *Pteropus hypomelanus*  *Rousettus leschenaultii*  *Rousettus amplexicaudatus*  *Hipposideros larvatus* | Walsh MG.^3^ | The risk of NiV spillover events in South and Southeast Asia | Model: MaxEnt and inhomogeneous Poisson point process model. | Ecological niche of *Pteropus* bats was used in the spatially explicit analysis of NiV spillover events |
|  | Deka MA, et al.^13^ | The distribution of NiV transmission risk in South and Southeast Asia | Model: MaxEnt | Measuring niche overlap between *Pteropus* bats and NiV |

**Reference**:

1. McKee CD, Islam A, Luby SP, et al. The Ecology of Nipah Virus in Bangladesh: A Nexus of Land-Use Change and Opportunistic Feeding Behavior in Bats. *Viruses*. 2021; **13**: 169.
2. Daszak P, Zambrana-Torrelio C, Bogich TL, et al. Interdisciplinary approaches to understanding disease emergence: the past, present, and future drivers of Nipah virus emergence. *Proc Natl Acad Sci U S A*. 2013; **110 Suppl 1**: 3681-3688.
3. Walsh MG. Mapping the risk of Nipah virus spillover into human populations in South and Southeast Asia. *Trans R Soc Trop Med Hyg*. 2015; **109**: 563-571.
4. Peterson AT. Mapping risk of Nipah virus transmission across Asia and across Bangladesh. *Asia Pac J Public Health*. 2015; **27**: NP824-NP832.
5. Nyakarahuka L, Ayebare S, Mosomtai G, et al. Ecological Niche Modeling for Filoviruses: A Risk Map for Ebola and Marburg Virus Disease Outbreaks in Uganda. **PLoS Curr**. 2017; **9**: ecurrents.outbreaks.07992a87522e1f229c7cb023270a2af1.
6. Muylaert RL, Kingston T, Luo J, et al. Present and future distribution of bat hosts of sarbecoviruses: implications for conservation and public health. *Proc Biol Sci*. 2022; **289**: 20220397.
7. Zhang F, Chase-Topping M, Guo CG, van Bunnik BAD, Brierley L, Woolhouse MEJ. Global discovery of human-infective RNA viruses: A modelling analysis. *PLoS Pathog*. 2020; **16**: e1009079.
8. Hassanin A, Tu VT, Curaudeau M, Csorba G. Inferring the ecological niche of bat viruses closely related to SARS-CoV-2 using phylogeographic analyses of *Rhinolophus* species. *Sci Rep*. 2021; **11**: 14276.
9. Han Y, Xu P, Wang Y, et al. Panoramic analysis of coronaviruses carried by representative bat species in Southern China to better understand the coronavirus sphere. *Nat Commun*. 2023; **14**: 5537.
10. Brierley L, Vonhof MJ, Olival KJ, Daszak P, Jones KE. Quantifying Global Drivers of Zoonotic Bat Viruses: A Process-Based Perspective. *Am Nat*. 2016; **187**: E53-E64.
11. Pigott DM, Golding N, Mylne A, et al. Mapping the zoonotic niche of Ebola virus disease in Africa. *Elife*. 2014; **3**: e04395.
12. Escobar LE, Peterson AT, Papeş M, et al. Ecological approaches in veterinary epidemiology: mapping the risk of bat-borne rabies using vegetation indices and night-time light satellite imagery. *Vet Res*. 2015; **46**: 92.
13. Deka MA, Morshed N. Mapping Disease Transmission Risk of Nipah Virus in South and Southeast Asia. *Trop Med Infect Dis*. 2018; **3**: 57.

Appendix Table S11: Occurrence data for bats using to build the ecological niche model, which were extracted from DarkCideS 1.0, Bat Eco-Interaction, GBIF, iDigBio, iNaturalist, PubMed, CNKI, NiV dataset.

| **Dataset** | ***Pteropus lylei*** | ***Pteropus medius*** | ***Pteropus vampyrus*** | ***Pteropus hypomelanus*** | ***Rousettus leschenaultii*** | ***Rousettus amplexicaudatus*** | ***Hipposideros larvatus*** | **Total** |
| --- | --- | --- | --- | --- | --- | --- | --- | --- |
| DarkCideS 1.0 | - | - | - | - | 33 | 24 | 21 | 78 |
| Bat Eco-Interaction | 2 | 10 | 4 | 1 | 6 | 3 | 2 | 28 |
| GBIF | 20 | 3 | 186 | 59 | 103 | 379 | 181 | 931 |
| iDigBio | 7 | 27 | 70 | 54 | 51 | 247 | 61 | 517 |
| iNaturalist | 20 | 800 | 116 | 8 | 42 | 18 | 49 | 1053 |
| PubMed | 53 | 27 | 10 | 9 | 10 | 7 | 28 | 144 |
| CNKI | - | - | - | - | 19 | - | 23 | 42 |
| Nipah dataset | 17 | 16 | 4 | - | 1 | 1 | 1 | 40 |
| Total | 119 | 883 | 390 | 131 | 265 | 679 | 366 | 2833 |

# Appendix Table S12 Definition of NiV infection in reservoir hosts

| **Species** | **Detection methods** | **Reference ID** |
| --- | --- | --- |
| *Pteropus lylei* | RT-PCR/Viris isolation | 34, 56, 72, 100, 108, 111, 114 |
| *Pteropus medius* | PCR/RT-PCR/qRT-PCR/Virus isolation | 67, 83, 92, 95, 98, 103, 113, 118 |
| *Pteropus vampyrus* | RT-PCR/qRT-PCR/Virus isolation | 43, 55, 60, 69, 71, 102 |
| *Pteropus hypomelanus* | RT-PCR | 20 |
| *Rousettus leschenaultii* | RT-PCR | 109 |
| *Rousettus amplexicaudatus* | RT-qPCR | 69 |
| *Hipposideros larvatus* | RT-PCR | 72 |
| *Pipistrellus pipistrellus* | RT-PCR | 109 |

# Appendix Table S13: Risk of bias assessments for the meta-analysis of CFR and clinical manifestation proportions

| **Reference ID** | **Evaluation criteria** | | | | | | | | | | |
| --- | --- | --- | --- | --- | --- | --- | --- | --- | --- | --- | --- |
|  | **[1]** | **[2]** | **[3]** | **[4]** | **[5]** | **[6]** | **[7]** | **[8]** | **[9]** | **[10]** | **Study** |
| 2 | N | Y | Y | N | Y | Y | Y | Y | Y | Y | C |
| 4 | N | Y | Y | N | Y | Y | Y | Y | Y | Y | C |
| 5 | Y | Y | Y | Y | Y | Y | N | Y | Y | Y | D |
| 8 | Y | Y | Y | Y | Y | Y | Y | Y | Y | Y | D/C |
| 9 | Y | Y | Y | Y | Y | Y | Y | Y | Y | Y | D/C |
| 11 | Y | Y | Y | N | Y | Y | Y | Y | N | Y | C |
| 14 | Y | Y | N | N | Y | Y | Y | N | N | Y | C |
| 19 | Y | Y | Y | Y | Y | Y | Y | Y | Y | Y | C |
| 23 | Y | Y | Y | N | Y | Y | Y | Y | Y | Y | C |
| 25 | Y | Y | Y | N | Y | Y | Y | Y | Y | Y | D/C |
| 26 | Y | Y | Y | Y | Y | Y | Y | Y | Y | Y | C |
| 27 | Y | Y | Y | N | Y | Y | Y | Y | Y | Y | C |
| 28 | Y | Y | Y | Y | Y | Y | Y | Y | Y | Y | D/C |
| 29 | Y | Y | Y | Y | Y | Y | Y | Y | Y | Y | C |
| 36 | Y | Y | Y | Y | Y | Y | Y | Y | Y | Y | D/C |
| 38 | Y | Y | Y | Y | Y | Y | Y | Y | Y | Y | D/C |
| 39 | Y | Y | Y | Y | Y | Y | Y | Y | Y | Y | D/C |
| 42 | Y | Y | Y | N | N | Y | Y | Y | Y | Y | C |
| 45 | Y | Y | Y | Y | Y | N | N | Y | N | Y | D |
| 46 | Y | Y | Y | Y | Y | Y | Y | Y | Y | Y | C |
| 52 | Y | Y | Y | Y | Y | Y | Y | Y | Y | Y | C |
| 53 | Y | Y | Y | Y | Y | Y | Y | Y | Y | Y | C |
| 54 | Y | Y | Y | Y | Y | Y | Y | Y | Y | Y | C |
| 58 | Y | Y | Y | Y | Y | Y | Y | Y | N | Y | D |
| 59 | Y | Y | Y | Y | Y | Y | Y | Y | Y | Y | C |
| 62 | Y | Y | Y | Y | Y | N | N | Y | N | Y | D |
| 65 | Y | Y | Y | Y | Y | N | Y | Y | N | Y | D |
| 66 | Y | Y | Y | Y | Y | Y | Y | Y | Y | Y | C |
| 75 | Y | Y | Y | Y | Y | N | N | Y | N | Y | D |
| 77 | Y | Y | Y | Y | Y | N | Y | Y | Y | Y | D |
| 78 | Y | Y | Y | Y | Y | Y | N | Y | Y | Y | D |
| 82 | Y | Y | Y | Y | Y | Y | Y | Y | Y | Y | D/C |
| 84 | Y | Y | Y | Y | Y | Y | N | Y | N | Y | D |
| 85 | Y | Y | Y | Y | Y | Y | Y | Y | Y | Y | C |
| 91 | N | Y | Y | N | Y | Y | Y | Y | Y | Y | C |
| 97 | Y | Y | Y | Y | Y | Y | Y | Y | Y | Y | C |
| 101 | Y | Y | Y | Y | Y | Y | Y | Y | Y | Y | C |
| 104 | Y | Y | Y | Y | Y | Y | Y | Y | Y | Y | D |
| 105 | Y | Y | Y | Y | Y | Y | Y | Y | Y | Y | D/C |
| 106 | Y | Y | Y | Y | Y | N | N | Y | Y | Y | D |
| 110 | N | Y | Y | N | N | Y | Y | Y | Y | Y | C |
| 111 | Y | Y | Y | Y | Y | Y | Y | Y | N | Y | D/C |
| 115 | Y | Y | Y | Y | Y | N | N | Y | N | Y | D |
| 119 | Y | Y | Y | Y | Y | Y | Y | Y | Y | Y | C |

**Note**: Due to the lack of a specific tool for assessing the bias of CFR and clinical manifestation proportion, we resort to utilizing JBI’s critical appraisal tool to evaluate the bias risk in the literature. In each study, <2 N is considered as low bias risk, 2≤N<4 is considered as medium bias risk, and ≥ 4 N is considered as high bias risk.

**Criteria**: [1] Were there clear criteria for inclusion in the study? [2] Was the condition measured in a standard, reliable way for all participants included in the study? [3] Were valid methods used for identification of the condition for all participants included in the study? [4] Did the study have consecutive inclusion of participants? [5] Did the study have complete inclusion of participants? [6] Was there clear reporting of the demographics of the participants in the study? [7] Was there clear reporting of clinical information of the participants? [8] Were the outcomes or follow up results of cases clearly reported? [9] Was there clear reporting of the presenting site(s)/clinic(s)/demographic information? [10] Was statistical analysis appropriate? Y: Yes; N: No. Study: D: Case fatality rate; C: Clinical manifestation proportion.

**Reference**:

Moola S, Munn Z, Tufanaru C, Aromataris E, Sears K, Sfetcu R, Currie M, Lisy K, Qureshi R, Mattis P, Mu P. Chapter 7: Systematic reviews of etiology and risk. In: Aromataris E, Munn Z (Editors). JBI Manual for Evidence Synthesis. JBI, 2020.

# Appendix Table S14: Subgroup meta-analysis and meta-regression of the case fatality rates of NiV infection

| **Variable** | **Univariable analysis** | | | | | | **Multivariable analysis ^b^** | |
| --- | --- | --- | --- | --- | --- | --- | --- | --- |
|  | **N studies** | **Case fatality rate (95% CI) (%) ^a^** | **Coefficient (95% CI)** | ***p* value** | **R^2^, %** | ***I*², %** | **Coefficient (95% CI)** | ***p* value** |
| Clade | 31 |  |  |  | 40·2 | 80·9 |  |  |
| Bangladesh-clade | 26 | 76·7 (69·0, 83·6) |  |  |  |  |  |  |
| Malaysia-clade | 5 | 37·4 (24·1, 51·7) | -0·380 (-0·565, -0·195) | <0·001 |  |  | -1·603 (-2·516, -0·690) | <0·001 |
| Country | 31 |  |  |  | 36·4 | 82·1 |  |  |
| Bangladesh | 22 | 76·7 (68·4, 84·2) |  |  |  |  |  |  |
| India | 4 | 77·8 (52·7, 95·8) | -0·003 (-0·217, 0·211) | 0·979 |  |  | 0·002 (-0·220, 0·225) | 0·983 |
| Malaysia | 3 | 39·5 (24·9, 55·0) | -0·350 (-0·577, -0·122) | 0·003 |  |  | 1·003 (0·188, 1·817) | 0·016 |
| Philippines | 1 | 52·9 (28·8, 76·4) | -0·233 (-0·649, 0·183) | 0·272 |  |  | 1·211 (0·152, 2·269) | 0·025 |
| Singapore | 1 | 9·1(0·0, 35·0) | -0·690 (-1·139, -0·241) | 0·003 |  |  |  |  |
| Publish Year | 31 |  |  |  | 0·0 | 88·2 |  |  |
| <2010 | 14 | 66·3 (53·0, 78·4) |  |  |  |  |  |  |
| 2010‒2019 | 9 | 72·8 (54·8, 88·0) | 0·088 (-0·134, 0·310) | 0·439 |  |  |  |  |
| ≥2020 | 8 | 75·3 (57·5, 90·0) | 0·064 (-0·147, 0·275) | 0·553 |  |  |  |  |
| Mean age (years) ^c^ |  |  |  |  | 22·8 | 84·1 |  |  |
| ≥30 | 9 | 53·0 (36·5, 69·2) |  |  |  |  |  |  |
| 20‒30 | 7 | 75·9 (61·7, 87·8) | 0·240 (0·033, 0·447) | 0·023 |  |  |  |  |
| Unknown | 15 | 77·5 (66·7, 86·8) | 0·239 (0·054, 0·424) | 0·011 |  |  |  |  |
| Male proportion (%) |  |  |  |  | 25·8 | 83·8 |  |  |
| 40‒60 | 6 | 68·6 (54·2, 81·4) |  |  |  |  |  |  |
| 60‒80 | 7 | 72·7 (59·0, 84·5) | 0·047 (-0·174, 0·268) | 0·680 |  |  | 0·163 (-0·075, 0·401) | 0·179 |
| 80‒100 | 6 | 51·4 (28·3, 74·3) | -0·185 (-0·426, 0·056) | 0·132 |  |  | 0·372 (0·031, 0·713) | 0·033 |
| Unknown | 12 | 80·9 (70·1, 90·0) | 0·104 (-0·111, 0·320) | 0·342 |  |  | 0·067 (-0·140, 0·275) | 0·524 |
| Risk of bias |  |  |  |  | 4·5 | 87·0 |  |  |
| Low | 19 | 66·9 (55·6, 77·3) |  |  |  |  |  |  |
| Moderate | 12 | 79·0 (79·8, 86·3) | 0·098 (-0·079, 0·276) | 0·278 |  |  |  |  |
| GDP per capita ($) ^d^ |  |  |  |  | 37·6 | 81·0 |  |  |
| High income (>12000) | 2 | 23·3 (6·2, 46·0） |  |  |  |  |  |  |
| Higher middle income (4001‒12000) | 2 | 47·4 (16·1, 79·8) | 0·242 (-0·134, 0·619) | 0·207 |  |  |  |  |
| Lower middle income (2001‒4000) | 11 | 81·3 (71·6, 89·6) | 0·586 (0·287, 0·886) | <0·001 |  |  |  |  |
| Low income (≤2000) | 16 | 72·5 (62·5, 81·5) | 0·496 (0·208, 0·783) | <0·001 |  |  |  |  |
| HDI ^e^ |  |  |  |  | 23·6 | 84·0 |  |  |
| High or very high (>0·700) | 5 | 46·6 (23·3, 70·6) |  |  |  |  |  |  |
| Medium (0·550‒0·700) | 14 | 77·5 (65·6, 87·8) | 0·298 (0·078, 0·518) | 0·008 |  |  |  |  |
| Low (<0·550) | 12 | 72·8 (62·1, 82·4) | 0·258 (0·040, 0·477) | 0·020 |  |  |  |  |
| Gini ^f^ |  |  |  |  | 0·7 | 88·1 |  |  |
| Equality (<0·5) | 29 | 72·4 (63·7, 80·4) |  |  |  |  |  |  |
| Inequality (≥0·5) | 2 | 36·6 (0,0, 91·8) | -0·331 (-0·688, 0·026) | 0·069 |  |  | 0·550 (-0·051, 1·152) | 0·073 |
| Travel time to healthcare facilities (min) ^g^ |  |  |  |  | 17·1 | 85·4 |  |  |
| Fast (<30) | 17 | 78·6 (68·7, 87·4) |  |  |  |  |  |  |
| Medium (30‒60) | 11 | 65·2 (51·6, 77·7) | -0·130 (-0·299, 0·039) | 0·131 |  |  | -0·206 (-0·397, -0·016) | 0·033 |
| Slow (>60) | 3 | 48·4 (26·3, 70·7) | -0·289 (-0·560, -0·019) | 0·036 |  |  | -0·204 (-0·696, 0·288) | 0·417 |

**Note**:

^a^ Case fatality rate was obtained from subgroup meta-analysis.

^b^ Residual heterogeneity I²=79·0%; Explained heterogeneity R² of the model=47·0%; Permutation testing p=0·021.

^c^ Mean age was directly pooled from the article or estimated using R package “estmeansd”.

^d^ GDP per capita at study-level was extracted and calculated from global 1km×1km gridded data for GDP during 1992-2019 and global population counts during 2000-2020 (1km resolution). ^1-2^ The ranges of GDP per capita were based on the country classification criteria of World Bank by income (https://datahelpdesk.worldbank.org/knowledgebase/articles/378834-how-does-the-world-bank-classify-countries), and were adjusted at the integer level.

^e^ HDI was obtained from Global Data Lab (https://globaldatalab.org/shdi/) at the first-level administrative division, and the classification was based on the standards of the United Nations Development Programme (https://hdr.undp.org/data-center/human-development-index#/indicies/HDI).^3^

^f^ Gini index at study level were extracted and calculated from global gridded Gini coefficient at a spatial resolution of 1°.^4^

^g^ Travel time to healthcare facilities was extracted and calculated from the global map of optimal travel time to healthcare with access to motorized transport.^5^

**Reference**:

1. Chen J, Gao M, Cheng S, et al. Global 1 km × 1 km gridded revised real gross domestic product and electricity consumption during 1992-2019 based on calibrated nighttime light data. *Sci Data*. 2022; **9**: 202.

2. WorldPop (www.worldpop.org - School of Geography and Environmental Science, University of Southampton; Department of Geography and Geosciences, University of Louisville; Departement de Geographie, Universite de Namur) and Center for International Earth Science Information Network (CIESIN), Columbia University (2018). Global High-Resolution Population Denominators Project - Funded by The Bill and Melinda Gates Foundation (OPP1134076).

3. Data retrieved from the Subnational HDI Database of the Global Data Lab, https://globaldatalab.org/shdi/, version v5.0.

4. Mirza MU, Xu C, Bavel BV, van Nes EH, Scheffer M. Global inequality remotely sensed. *Proc Natl Acad Sci U S A*. 2021;**118**: e1919913118.

5. Weiss DJ, Nelson A, Vargas-Ruiz CA, et al. Global maps of travel time to healthcare facilities. *Nat Med*. 2020; **26**: 1835-1838.

# Appendix Table S15: Sensitivity analysis of meta-analysis of the case fatality rates of NiV infection

| **Omitting study** | **Proportion (95% CI)** | **tau^2^** | **tau** | ***I*^2^** |
| --- | --- | --- | --- | --- |
| Rahman MZ [111] | 0·696 (0·608, 0·779) | 0·045 | 0·212 | 0·880 |
| Naser AM [78] | 0·716 (0·630, 0·796) | 0·042 | 0·206 | 0·860 |
| Hegde ST [84] | 0·702 (0·610, 0·787) | 0·049 | 0·221 | 0·877 |
| Luby SP [49] | 0·705 (0·612, 0·790) | 0·050 | 0·223 | 0·883 |
| Hossain MJ [46] | 0·704 (0·612, 0·788) | 0·049 | 0·220 | 0·883 |
| Luby SP [38] | 0·697 (0·609, 0·779) | 0·045 | 0·212 | 0·882 |
| Chakraborty A [82] | 0·696 (0·608, 0·778) | 0·044 | 0·211 | 0·877 |
| Arankalle VA [58] | 0·696 (0·608, 0·777) | 0·045 | 0·212 | 0·882 |
| Islam MS [85] | 0·709 (0·620, 0·791) | 0·046 | 0·214 | 0·884 |
| Gurley ES [39] | 0·703 (0·613, 0·786) | 0·047 | 0·216 | 0·883 |
| Sim BF [25] | 0·706 (0·617, 0·788) | 0·046 | 0·215 | 0·884 |
| Goh KJ [9] | 0·720 (0·637, 0·797) | 0·039 | 0·198 | 0·858 |
| Chua KB [8] | 0·720 (0·636, 0·797) | 0·040 | 0·201 | 0·862 |
| Hsu VP [28] | 0·705 (0·616, 0·787) | 0·046 | 0·215 | 0·884 |
| Ching PK [77] | 0·710 (0·622, 0·792) | 0·046 | 0·214 | 0·883 |
| Paton NI [5] | 0·722 (0·637, 0·800) | 0·043 | 0·207 | 0·876 |
| Sahay RR [106] | 0·695 (0·607, 0·778) | 0·045 | 0·211 | 0·879 |
| IEDCR [75] | 0·702 (0·615, 0·784) | 0·046 | 0·214 | 0·884 |
| IEDCR [115] | 0·698 (0·609, 0·780) | 0·045 | 0·213 | 0·882 |
| Lo MK [65] | 0·698 (0·610, 0·781) | 0·045 | 0·213 | 0·882 |
| IEDCR [115] | 0·707 (0·618, 0·789) | 0·046 | 0·214 | 0·884 |
| IEDCR [75] | 0·710 (0·623, 0·791) | 0·045 | 0·213 | 0·883 |
| Saha R· MS [105] | 0·714 (0·625, 0·796) | 0·046 | 0·215 | 0·879 |
| Lee KH [104] | 0·697 (0·609, 0·780) | 0·044 | 0·210 | 0·872 |
| Chadha MS [36] | 0·703 (0·612, 0·787) | 0·048 | 0·218 | 0·883 |
| Homaira N [45] | 0·703 (0·615, 0·785) | 0·046 | 0·214 | 0·884 |
| IEDCR [115] | 0·696 (0·608, 0·777) | 0·045 | 0·212 | 0·882 |
| IEDCR [62] | 0·703 (0·614, 0·786) | 0·046 | 0·215 | 0·883 |
| Chakraborty A [82] | 0·693 (0·605, 0·775) | 0·044 | 0·209 | 0·877 |
| IEDCR [115] | 0·706 (0·617, 0·788) | 0·046 | 0·214 | 0·884 |
| IEDCR [115] | 0·710 (0·623, 0·792) | 0·046 | 0·213 | 0·883 |

**Note**: Pooled estimate proportion (95% CI): 0·705 (0·618, 0·785), tau^2^=0·045, tau=0·213, *I*^2^=0·880

# Appendix Table S16: Subgroup meta-analysis of NiV positive detection rate in reservoir hosts

| **Variables** | **N testing** | **N positive** | **Crude detection rate** | ***I*^2^ (%)** | **Detection rate (95% CI)**  **(Random effect)** | **Detection rate (95% CI)**  **(Fixed effect)** |
| --- | --- | --- | --- | --- | --- | --- |
| Species |  |  |  |  |  |  |
| *Pteropus lylei* | 8833 | 183 | 0·0207 | 76·5 | 0·0135 (0·0069, 0·0216) | 0·0060 (0·0039, 0·0083) |
| *Pteropus medius* | 6419 | 72 | 0·0112 | 41·6 | 0·0023 (0·0004, 0·0052) | 0·0031 (0·0014, 0·0053) |
| *Pteropus vampyrus* | 4186 | 36 | 0·0086 | 44·6 | 0·0043 (0·0000, 0·0156) | 0·0000 (0·0000, 0·0002) |
| *Pteropus hypomelanus* | 765 | 8 | 0·0105 | 50·4 | 0·0100 (0·0000, 0·0378) | 0·0007 (0·0000, 0·0067) |
| *Rousettus leschenaultii* | 150 | 5 | 0·0333 | 51·5 | 0·0288 (0·0000, 0·1028) | 0·0137 (0·0000, 0·0479) |
| *Rousettus amplexicaudatus* | 60 | 5 | 0·0833 | 44·4 | 0·0769 (0·0060, 0·1978) | 0·0769 (0·0182, 0·1632) |
| *Pipistrellus Pipistrellus* | 50 | 3 | 0·0600 | 40·7 | 0·0368 (0·0000, 0·1568) | 0·0311 (0·0000, 0·1147) |
| Country |  |  |  |  |  |  |
| Bangladesh | 5954 | 49 | 0·0082 | 16·5 | 0·0017 (0·0003, 0·0039) | 0·0028 (0·0011, 0·0049) |
| India | 665 | 31 | 0·0466 | 39·9 | 0·0305 (0·0116, 0·0551) | 0·0272 (0·0133, 0·0443) |
| Malaysia | 649 | 6 | 0·0092 | 62·9 | 0·0194 (0·0000, 0·0853) | 0·0000 (0·0000, 0·0036) |
| Thailand | 4311 | 158 | 0·0367 | 69·6 | 0·0144 (0·0070, 0·0235) | 0·0183 (0·0137, 0·0234) |
| Cambodia | 8629 | 58 | 0·0067 | 23·7 | 0·0065 (0·0045, 0·0088) | 0·0064 (0·0048, 0·0083) |
| Indonesia | 144 | 4 | 0·0278 | 0·0 | 0·0161 (0·0000, 0·0519) | 0·0161 (0·0000, 0·0519) |
| East Timor | 111 | 6 | 0·0541 | 48·3 | 0·0498 (0·0039, 0·1287) | 0·0456 (0·0107, 0·0969) |
| Detection method |  |  |  |  |  |  |
| RT-PCR | 14126 | 269 | 0·0190 | 73·7 | 0·0150 (0·0092, 0·0217) | 0·0041 (0·0026, 0·0058) |
| PCR | 4755 | 23 | 0·0048 | 0 | 0·0006 (0·0000, 0·0021) | 0·0006 (0·0000, 0·0021) |
| RT-qPCR | 111 | 6 | 0·0541 | 48·3 | 0·0498 (0·0039, 0·1287) | 0·0456 (0·0107, 0·0969) |
| Virus isolation | 1471 | 14 | 0·0095 | 81·2 | 0·0030 (0·0000, 0·0235) | 0·0008 (0·0000, 0·0047) |
| Publish year |  |  |  |  |  |  |
| <2010 | 4095 | 51 | 0·0125 | 32·0 | 0·0053 (0·0017, 0·0102) | 0·0026 (0·0007, 0·0054) |
| 2010–2020 | 14491 | 140 | 0·0097 | 46·7 | 0·0029 (0·0009, 0·0055) | 0·0019 (0·0009, 0·0031) |
| >2020 | 1877 | 121 | 0·0645 | 80·9 | 0·0377 (0·0161, 0·0656) | 0·0371 (0·0276, 0·0477) |
| Overall | 20463 | 312 | 0·0152 | 66·3 | 0·0074 (0·0044, 0·0110) | 0·0030 (0·0020, 0·0043) |

# Appendix Table S17: Locations with NiV infection in animals and humans used to build BRT models

| **Point/Polygon** | **Latitude** | **Longitude** | **Positive Hosts** | **Country** | **Reference ID** |
| --- | --- | --- | --- | --- | --- |
| Polygon | -9.2440 | 125.2000 | Bat | East Timor | 69 |
| Polygon | 1.3587 | 103.8400 | Human | Singapore | 5, 15, 18, 27 |
| Point | 1.4415 | 103.7232 | Human | Malaysia | 3 |
| Polygon | 2.5347 | 101.8920 | Human | Malaysia | 7, 10 |
| Point | 2.6146 | 101.8012 | Human | Malaysia | 3 |
| Point | 2.6250 | 101.7605 | Human | Malaysia | 3 |
| Point | 2.6439 | 101.7535 | Human | Malaysia | 2, 3, 12, 24, 63 |
| Point | 2.6996 | 101.7502 | Human | Malaysia | 25 |
| Point | 2.7118 | 101.6588 | Human | Malaysia | 25 |
| Polygon | 2.7518 | 101.9180 | Human/Pig | Malaysia | 19, 32 |
| Polygon | 2.7741 | 102.2000 | Human | Malaysia | 1, 11, 51 |
| Point | 2.8032 | 101.8782 | Human | Malaysia | 25 |
| Polygon | 2.8700 | 103.1100 | Bat | Malaysia | 20 |
| Polygon | 3.1327 | 101.7000 | Human | Malaysia | 9, 22 |
| Point | 3.1993 | 101.6222 | Human | Malaysia | 25 |
| Point | 3.2100 | 101.5500 | Human/Pig | Malaysia | 24, 32 |
| Point | 3.2541 | 101.4394 | Human | Malaysia | 25 |
| Polygon | 3.2708 | 101.5000 | Human | Malaysia | 1, 11, 51 |
| Point | 3.6199 | 101.0383 | Human | Malaysia | 3, 24 |
| Polygon | 3.7010 | 98.6260 | Bat | Indonesian | 71 |
| Polygon | 4.5151 | 101.1580 | Human | Malaysia | 24 |
| Polygon | 4.6091 | 101.0932 | Human | Malaysia | 3 |
| Point | 4.6113 | 101.1401 | Human/Pig | Malaysia | 3, 32 |
| Point | 4.6922 | 101.0860 | Human | Malaysia | 25 |
| Polygon | 5.1170 | 100.9758 | Bat | Malaysia | 55, 60 |
| Polygon | 6.4404 | 124.2900 | Human | Philippines | 77 |
| Polygon | 6.4959 | 124.2970 | Human | Philippines | 77 |
| Point | 9.8788 | 76.4733 | Human | India | 117 |
| Point | 9.8843 | 76.7610 | Bat | India | 113 |
| Polygon | 9.9487 | 76.3428 | Human | India | 106, 113 |
| Point | 10.1728 | 76.3884 | Bat | India | 113 |
| Point | 11.0009 | 75.9314 | Human | India | 91 |
| Polygon | 11.2420 | 76.2088 | Human | India | 93 |
| Point | 11.3083 | 75.9106 | Human | India | 116 |
| Polygon | 11.3506 | 75.932 | Human/Bat | India | 100, 107, 112 |
| Polygon | 11.4400 | 105.0000 | Bat | Cambodia | 102, 108 |
| Polygon | 11.5597 | 75.7581 | Bat | India | 98 |
| Point | 11.6149 | 75.7777 | Human | India | 87, 97, 99, 100, 101, 106 |
| Polygon | 11.6549 | 75.7544 | Bat | India | 98 |
| Point | 12.9674 | 103.1811 | Bat | Cambodia | 34 |
| Point | 12.9800 | 103.1000 | Bat | Cambodia | 108 |
| Polygon | 13.1900 | 101.2000 | Bat | Thailand | 72 |
| Point | 13.5053 | 101.1653 | Bat | Thailand | 56 |
| Polygon | 13.5100 | 101.1700 | Bat | Thailand | 72, 114 |
| Point | 13.7223 | 101.2019 | Bat | Thailand | 56 |
| Polygon | 13.7700 | 100.4300 | Bat | Thailand | 72 |
| Polygon | 13.7700 | 100.6000 | Bat | Thailand | 72 |
| Polygon | 13.7800 | 102.3000 | Bat | Thailand | 72 |
| Polygon | 14.0500 | 101.6000 | Bat | Thailand | 72 |
| Point | 14.0573 | 101.3504 | Bat | Thailand | 56 |
| Point | 14.1537 | 100.5041 | Bat | Thailand | 56 |
| Polygon | 14.3400 | 100.5000 | Bat | Thailand | 72 |
| Point | 14.3407 | 100.8689 | Bat | Thailand | 56 |
| Polygon | 14.6200 | 100.4000 | Bat | Thailand | 72 |
| Point | 14.6704 | 100.3756 | Bat | Thailand | 56 |
| Point | 14.7664 | 100.4467 | Bat | Thailand | 56 |
| Polygon | 14.9100 | 100.3000 | Bat | Thailand | 72 |
| Polygon | 17.8991 | 73.6764 | Bat | India | 109 |
| Polygon | 22.8990 | 90.8530 | Bat | Bangladesh | 95 |
| Polygon | 23.0497 | 89.8441 | Human | Bangladesh | 29 |
| Point | 23.1707 | 89.8752 | Human | Bangladesh | 49 |
| Polygon | 23.1998 | 90.1782 | Human | Bangladesh | 65, 75 |
| Polygon | 23.2229 | 90.3232 | Human | Bangladesh | 75 |
| Point | 23.2389 | 89.8711 | Human | Bangladesh | 54 |
| Point | 23.2484 | 89.9900 | Human | Bangladesh | 54 |
| Point | 23.2484 | 90.1471 | Human | Bangladesh | 54 |
| Polygon | 23.2593 | 89.9210 | Human | Bangladesh | 111 |
| Point | 23.2921 | 89.6896 | Human | Bangladesh | 49 |
| Point | 23.3417 | 89.6677 | Human | Bangladesh | 82 |
| Point | 23.3570 | 89.8040 | Human | Bangladesh | 76 |
| Point | 23.3600 | 89.7534 | Human | Bangladesh | 76 |
| Point | 23.3711 | 89.9367 | Human | Bangladesh | 57 |
| Polygon | 23.3778 | 90.1688 | Human | Bangladesh | 111 |
| Polygon | 23.3863 | 90.0132 | Human | Bangladesh | 54, 62 |
| Point | 23.3920 | 89.6325 | Human | Bangladesh | 49 |
| Point | 23.3934 | 90.0010 | Human | Bangladesh | 66 |
| Point | 23.4195 | 89.6493 | Human | Bangladesh | 76 |
| Point | 23.4206 | 89.7895 | Human | Bangladesh | 49 |
| Point | 23.4388 | 89.6997 | Human | Bangladesh | 54 |
| Polygon | 23.4453 | 89.4134 | Human | Bangladesh | 75, 81, 111 |
| Point | 23.4492 | 91.1573 | Human | Bangladesh | 76 |
| Point | 23.4673 | 89.9520 | Human | Bangladesh | 54 |
| Point | 23.5057 | 89.5869 | Human | Bangladesh | 76 |
| Point | 23.5236 | 89.7266 | Human | Bangladesh | 76 |
| Point | 23.5355 | 89.4249 | Human | Bangladesh | 66 |
| Point | 23.5625 | 89.9044 | Human | Bangladesh | 54 |
| Polygon | 23.6010 | 89.8590 | Bat | Bangladesh | 83, 103 |
| Polygon | 23.6052 | 89.8195 | Human | Bangladesh | 29, 31, 46, 57, 70, 74, 75, 78, 79, 81, 88, 94, 111 |
| Point | 23.6065 | 88.7463 | Human | Bangladesh | 66 |
| Point | 23.6291 | 89.5094 | Human | Bangladesh | 54 |
| Point | 23.6396 | 89.5839 | Human | Bangladesh | 76 |
| Polygon | 23.6530 | 89.5530 | Human | Bangladesh | 48, 30, 31, 46, 61, 65, 66, 75, 78, 79, 81, 84, 85 |
| Point | 23.6668 | 88.6652 | Human | Bangladesh | 49 |
| Point | 23.7094 | 89.6823 | Human | Bangladesh | 82 |
| Point | 23.7150 | 89.6020 | Bat | Bangladesh | 103 |
| Point | 23.7238 | 89.9180 | Bat | Bangladesh | 118 |
| Point | 23.7274 | 89.7640 | Human | Bangladesh | 76 |
| Polygon | 23.7385 | 88.6516 | Human | Bangladesh | 26, 28, 46 |
| Point | 23.7407 | 89.6374 | Human | Bangladesh | 76 |
| Polygon | 23.7533 | 89.7691 | Human | Bangladesh | 29 |
| Point | 23.7561 | 90.2821 | Human | Bangladesh | 49 |
| Point | 23.7605 | 89.6385 | Human | Bangladesh | 82 |
| Point | 23.7668 | 89.7181 | Human | Bangladesh | 49 |
| Point | 23.7682 | 88.5165 | Human | India | 58 |
| Point | 23.7880 | 89.1093 | Human | Bangladesh | 66 |
| Point | 23.7918 | 88.7770 | Human | Bangladesh | 38 |
| Point | 23.8180 | 89.7772 | Human | Bangladesh | 76 |
| Point | 23.8195 | 89.2667 | Human | Bangladesh | 54 |
| Polygon | 23.8213 | 89.0928 | Human | Bangladesh | 44, 53, 75 |
| Polygon | 23.8356 | 90.0263 | Human | Bangladesh | 29, 30, 31, 74, 75 |
| Polygon | 23.8386 | 89.8136 | Human | Bangladesh | 73 |
| Point | 23.8453 | 89.7859 | Human | Bangladesh | 49 |
| Point | 23.8524 | 89.1042 | Human | Bangladesh | 49 |
| Point | 23.8906 | 89.8826 | Human | Bangladesh | 66 |
| Polygon | 23.9571 | 89.8010 | Human | Bangladesh | 48, 66 |
| Point | 23.9809 | 89.2220 | Human | Bangladesh | 49 |
| Polygon | 24.0079 | 89.2824 | Human | Bangladesh | 73, 111 |
| Point | 24.0641 | 89.2001 | Human | Bangladesh | 76 |
| Point | 24.0985 | 89.2087 | Bat | Bangladesh | 118 |
| Point | 24.1344 | 89.9822 | Human | Bangladesh | 49 |
| Point | 24.1737 | 89.9715 | Human | Bangladesh | 49 |
| Point | 24.1831 | 89.9883 | Human | Bangladesh | 76 |
| Point | 24.1945 | 90.0280 | Human | Bangladesh | 38 |
| Point | 24.1950 | 90.0002 | Human | Bangladesh | 76 |
| Polygon | 24.2052 | 88.8282 | Human | Bangladesh | 111 |
| Point | 24.2220 | 90.4271 | Human | Bangladesh | 66 |
| Point | 24.2247 | 89.0543 | Human | Bangladesh | 76 |
| Polygon | 24.2272 | 90.0406 | Human | Bangladesh | 33 |
| Polygon | 24.2295 | 89.3093 | Human | Bangladesh | 111 |
| Point | 24.2396 | 89.1049 | Human | Bangladesh | 76 |
| Point | 24.2764 | 88.7583 | Bat | Bangladesh | 118 |
| Point | 24.2772 | 88.9686 | Human | Bangladesh | 49 |
| Point | 24.2950 | 89.0186 | Human | Bangladesh | 49 |
| Point | 24.3728 | 90.1374 | Human | Bangladesh | 38 |
| Point | 24.4021 | 88.5903 | Human | Bangladesh | 49 |
| Polygon | 24.4449 | 88.9827 | Human | Bangladesh | 73, 75 |
| Polygon | 24.5494 | 88.2686 | Human | Bangladesh | 75 |
| Polygon | 24.7443 | 88.7118 | Human | Bangladesh | 111 |
| Point | 24.7483 | 88.6616 | Human | Bangladesh | 49 |
| Point | 24.7876 | 88.6581 | Human | Bangladesh | 49 |
| Point | 24.8000 | 88.6333 | Human | Bangladesh | 26, 28 |
| Polygon | 24.8077 | 88.8983 | Human | Bangladesh | 26, 28, 29, 31, 46, 73, 75, 80, 81, 84 |
| Point | 24.8466 | 88.7488 | Bat | Bangladesh | 118 |
| Point | 24.8697 | 88.5795 | Human | Bangladesh | 49 |
| Point | 24.9638 | 88.5569 | Human | Bangladesh | 66 |
| Polygon | 24.9649 | 88.9410 | Human | Bangladesh | 111 |
| Point | 24.9963 | 88.7061 | Human | Bangladesh | 38 |
| Point | 25.0161 | 89.0861 | Bat | Bangladesh | 118 |
| Point | 25.0695 | 88.9793 | Human | Bangladesh | 49 |
| Point | 25.0994 | 89.0861 | Bat | Bangladesh | 118 |
| Polygon | 25.1121 | 89.0195 | Human/Bat | Bangladesh | 29, 31, 68, 111 |
| Polygon | 25.1294 | 88.8226 | Human | Bangladesh | 110 |
| Polygon | 25.2636 | 89.3970 | Human | Bangladesh | 111 |
| Point | 25.3133 | 89.2298 | Human | Bangladesh | 76 |
| Polygon | 25.3480 | 89.5686 | Human | Bangladesh | 73 |
| Polygon | 25.6069 | 88.6784 | Human | Bangladesh | 75, 82 |
| Point | 25.6145 | 88.9086 | Human | Bangladesh | 82 |
| Point | 25.6729 | 88.5363 | Human | Bangladesh | 82 |
| Point | 25.7094 | 89.2297 | Human | Bangladesh | 82 |
| Point | 25.7208 | 89.2893 | Human | Bangladesh | 76 |
| Point | 25.7824 | 89.3319 | Human | Bangladesh | 82 |
| Point | 25.7922 | 89.4380 | Human | Bangladesh | 76 |
| Point | 25.8043 | 88.5728 | Human | Bangladesh | 82 |
| Polygon | 25.8045 | 88.9230 | Human | Bangladesh | 111 |
| Point | 25.8071 | 89.2417 | Human | Bangladesh | 76 |
| Polygon | 25.8777 | 88.1563 | Human | Bangladesh | 52 |
| Point | 25.8940 | 88.1869 | Human | Bangladesh | 49 |
| Polygon | 25.9405 | 88.8587 | Human | Bangladesh | 81 |
| Point | 25.9409 | 89.6284 | Human | Bangladesh | 76 |
| Polygon | 25.9482 | 89.5755 | Human | Bangladesh | 111 |
| Point | 25.9795 | 89.6969 | Human | Bangladesh | 82 |
| Polygon | 26.0789 | 88.4659 | Human | Bangladesh | 44 |
| Point | 26.0963 | 88.8283 | Human | Bangladesh | 82 |
| Polygon | 26.1015 | 90.0291 | Bat | India | 92 |
| Polygon | 26.1194 | 89.1484 | Human | Bangladesh | 59, 62 |
| Point | 26.1255 | 89.1129 | Human | Bangladesh | 82 |
| Point | 26.1395 | 88.4228 | Human | Bangladesh | 66 |
| Polygon | 26.3367 | 89.4216 | Bat | India | 92 |
| Polygon | 26.3860 | 88.6044 | Human | Bangladesh | 81 |
| Polygon | 26.5700 | 88.8200 | Bat | India | 67 |
| Polygon | 26.6582 | 88.2895 | Human | India | 36 |
| Point | 26.8598 | 88.4369 | Human | Bangladesh | 38 |
| Point | 23.6693 | 89.6285 | Human | Bangladesh | 76 |
| Point | 23.6910 | 89.7711 | Human | Bangladesh | 54 |
| Polygon | 23.7033 | 89.6736 | Human | Bangladesh | 62 |
| Point | 23.7168 | 89.6253 | Human | Bangladesh | 49 |
| Point | 23.7195 | 89.4428 | Human | Bangladesh | 54 |

# Appendix Table S18: Literature search syntax of bat-borne virus in PubMed in the study area

| **Search** | **Query** |
| --- | --- |
| #1 | Virus [Title/Abstract] |
| #2 | (bat [Title/Abstract]) OR (chiroptera [Title/Abstract]) |
| #3 | #1 AND #2 |
| #4 | (South Asia [Title/Abstract]) OR (Southeast Asia [Title/Abstract]) OR (South-East Asia [Title/Abstract]) OR (China [Title/Abstract]) OR (Australia [Title/Abstract]) OR (Papua New Guinea [Title/Abstract]) OR (India [Title/Abstract]) OR (Pakistan [Title/Abstract]) OR (Bangladesh [Title/Abstract]) OR (Nepal [Title/Abstract]) OR (Bhutan [Title/Abstract]) OR (Sri Lanka [Title/Abstract]) OR (Indonesia [Title/Abstract]) OR (Philippines [Title/Abstract]) OR (Vietnam [Title/Abstract]) OR (Thailand [Title/Abstract]) OR (Myanmar [Title/Abstract]) OR (Malaysia [Title/Abstract]) OR (Cambodia [Title/Abstract]) OR (Lao [Title/Abstract]) OR (Singapore [Title/Abstract]) OR (East Timor [Title/Abstract]) OR (Brunei [Title/Abstract]) |
| #5 | #3 AND #4 |

# Appendix Table S19: Mean (95% percentiles) relative contributions of variables (95% percentiles) of host niche models in this study

| **Variable** | ***Pteropus lylei*** | ***Pteropus medius*** | ***Pteropus vampyrus*** | ***Pteropus hypomelanus*** | ***Rousettus leschenaultii*** | ***Rousettus amplexicaudatus*** | ***Hipposideros larvatus*** |
| --- | --- | --- | --- | --- | --- | --- | --- |
| Animal |  |  |  |  |  |  |  |
| Chiroptera richness | 32·0 (31·6, 32·5) | 20·3 (20·1, 20·5) | 21·1 (20·6, 21·6) | 9·3 (9, 9·7) | 5·6 (5·5, 5·8) | 18·8 (18·6, 18·9) | 47·1 (46·9, 47·3) |
| Environment |  |  |  |  |  |  |  |
| Annual mean temperature | 57·4 (56·8, 58·0) | 13·3 (13·2, 13·4) | 0·7 (0·7, 0·7) | 1·0 (1·0, 1·1) | 1·4 (1·3, 1·4) | 0·5 (0·5, 0·6) | 0·5 (0·5, 0·5) |
| Mean diurnal range | 0 | 1·3 (1·2, 1·3) | 4·3 (4·1, 4·4) | 18 (16·9, 19) | 1·5 (1·4, 1·6) | 2·7 (2·6, 2·7) | 4·8 (4·7, 4·9) |
| Isothermality | 2·5 (2·4, 2·7) | 1·8 (1·7, 1·8) | 2·7 (2·6, 2·8) | 3·9 (3·7, 4·1) | 2·4 (2·3, 2·5) | 3·0 (2·9, 3·1) | 5·4 (5·3, 5·6) |
| Temperature seasonality | 0·1 (0·1, 0·1) | 0·8 (0·8, 0·8) | 16·8 (16·1, 17·4) | 2·9 (2·6, 3·2) | 2·1 (2·0, 2·1) | 3·8 (3·7, 3·9) | 2·1 (2·0, 2·2) |
| Max temperature of warmest month | 0 | 1·4 (1·4, 1·4) | 0·9 (0·9, 1·0) | 0·5 (0·5, 0·6) | 2·5 (2·4, 2·6) | 0·8 (0·8, 0·8) | 1·0 (1·0, 1·0) |
| Min temperature of coldest month | 0 | 0·3 (0·3, 0·3) | 3·3 (3·2, 3·4) | 16·4 (15·6, 17·1) | 1·3 (1·3, 1·4) | 3·3 (3·2, 3·4) | 1·9 (1·9, 2·0) |
| Annual range of temperature | 0 | 0·8 (0·8, 0·9) | 16·9 (16·2, 17·6) | 11·9 (11, 12·8) | 2·8 (2·7, 3·0) | 30·0 (29·8, 30·3) | 1·9 (1·9, 2·0) |
| Mean temperature of wettest quarter | 0 | 0·8 (0·8, 0·8) | 0·7 (0·7, 0·8) | 1·0 (1·0, 1·1) | 1·4 (1·3, 1·4) | 0·6 (0·5, 0·6) | 1·0 (1·0, 1·0) |
| Mean temperature of driest quarter | 0·1 (0·1, 0·1) | 4·4 (4·3, 4·6) | 0·8 (0·8, 0·9) | 3·8 (3·4, 4·1) | 2·9 (2·9, 3·0) | 1·1 (1·0, 1·1) | 1·4 (1·3, 1·4) |
| Mean temperature of warmest quarter | 0·1 (0·1, 0·2) | 1·4 (1·4, 1·4) | 0·8 (0·8, 0·9) | 0·6 (0·5, 0·6) | 3·1 (3·0, 3·2) | 0·6 (0·6, 0·6) | 0·8 (0·7, 0·8) |
| Mean temperature of coldest quarter | 0·3 (0·3, 0·4) | 1·5 (1·5, 1·6) | 1·2 (1·2, 1·2) | 2·2 (2·1, 2·3) | 1·2 (1·1, 1·2) | 0·6 (0·6, 0·7) | 1·2 (1·2, 1·3) |
| Annual precipitation | 0·1 (0·1, 0·1) | 0·4 (0·3, 0·4) | 1·8 (1·7, 1·9) | 2·1 (2·0, 2·2) | 1·9 (1·8, 2·0) | 5·1 (5·0, 5·3) | 2·0 (2·0, 2·1) |
| Precipitation of wettest month | 0·2 (0·1, 0·2) | 3·4 (3·3, 3·5) | 1·2 (1·2, 1·3) | 1·5 (1·4, 1·6) | 4·7 (4·4, 5·0) | 0·7 (0·7, 0·7) | 2·3 (2·2, 2·3) |
| Precipitation of driest month | 0·2 (0·2, 0·2) | 2·1 (2·1, 2·2) | 0·9 (0·9, 0·9) | 3·0 (2·8, 3·2) | 4·1 (3·9, 4·3) | 2·6 (2·5, 2·6) | 1·5 (1·4, 1·6) |
| Precipitation seasonality | 0·5 (0·4, 0·6) | 4·6 (4·5, 4·7) | 2·4 (2·3, 2·4) | 2·7 (2·6, 2·9) | 4·8 (4·6, 4·9) | 1·0 (1·0, 1·1) | 1·1 (1·1, 1·1) |
| Precipitation of wettest quarter | 0 | 0·4 (0·4, 0·4) | 1·8 (1·8, 1·9) | 1·2 (1·1, 1·3) | 6·6 (6·3, 6·8) | 0·6 (0·6, 0·6) | 1·7 (1·7, 1·8) |
| Precipitation of driest quarter | 0·1 (0·0, 0·1) | 1·0 (0·9, 1·0) | 0·9 (0·9, 1·0) | 1·9 (1·8, 2·0) | 1·2 (1·2, 1·3) | 0·9 (0·8, 0·9) | 0·8 (0·7, 0·8) |
| Precipitation of warmest quarter | 0·2 (0·2, 0·2) | 1·8 (1·8, 1·9) | 1·6 (1·5, 1·7) | 3·6 (3·4, 3·8) | 6·4 (6·2, 6·5) | 1·3 (1·2, 1·3) | 1·9 (1·8, 1·9) |
| Precipitation of coldest quarter | 0·9 (0·8, 1·0) | 1·0 (1·0, 1·1) | 2·1 (2·0, 2·2) | 2·0 (1·8, 2·1) | 2·9 (2·8, 3·0) | 3·5 (3·5, 3·6) | 2·6 (2·5, 2·7) |
| Elevation | 3·1 (2·8, 3·4) | 2·6 (2·5, 2·6) | 2·3 (2·2, 2·3) | 0·4 (0·4, 0·4) | 3·5 (3·4, 3·6) | 1·5 (1·4, 1·5) | 1·7 (1·6, 1·8) |
| Forest | 0 | 1·0 (1·0, 1·0) | 1·2 (1·1, 1·2) | 1·0 (0·9, 1·1) | 1·4 (1·4, 1·5) | 0·4 (0·4, 0·4) | 1·2 (1·2, 1·3) |
| Grassland | 0 | 0·4 (0·4, 0·4) | 0 | 0 | 0·2 (0·2, 0·2) | 0·6 (0·6, 0·7) | 0·3 (0·2, 0·3) |
| Leaf area index | 0·3 (0·2, 0·4) | 0·5 (0·5, 0·6) | 1·1 (1·1, 1·1) | 1·7 (1·6, 1·9) | 1·9 (1·8, 2·0) | 2·2 (2·1, 2·3) | 1·1 (1·0, 1·1) |
| Mixed cropland and nature vegetation | 0·9 (0·8, 0·9) | 0·4 (0·4, 0·4) | 2·4 (2·4, 2·5) | 1·1 (1·0, 1·1) | 4·3 (4·1, 4·4) | 4·6 (4·5, 4·7) | 1·5 (1·5, 1·6) |
| Mixed tree, shrub and herbaceous | 0 | 3·0 (2·9, 3·1) | 0·4 (0·4, 0·4) | 0·2 (0·2, 0·3) | 11·2 (11, 11·5) | 3·3 (3·1, 3·4) | 4·9 (4·8, 5·0) |
| Vegetation flooded | 0·1 (0·1, 0·2) | 2·8 (2·7, 2·8) | 1·5 (1·4, 1·5) | 0·4 (0·4, 0·4) | 0·9 (0·9, 1·0) | 0·5 (0·5, 0·5) | 1·0 (1·0, 1·1) |
| Shrubland | 0 | 0·7 (0·6, 0·7) | 1·3 (1·2, 1·4) | 1·2 (1·1, 1·3) | 2·3 (2·2, 2·4) | 2·9 (2·8, 3·0) | 2·2 (2·1, 2·2) |
| Sparse vegetation land | 0 | 1·0 (0·9, 1·0) | 0·4 (0·4, 0·5) | 0·1 (0·1, 0·1) | 0·1 (0·1, 0·1) | 0·5 (0·5, 0·6) | 0 |
| Water body | 0 | 0·5 (0·5, 0·5) | 0·9 (0·8, 0·9) | 2·6 (2·5, 2·8) | 0·5 (0·5, 0·5) | 0·6 (0·6, 0·7) | 1·1 (1·0, 1·1) |
| Bare areas | 0 | 0·1 (0·1, 0·1) | 0 | 0 | 0 | 0 | 0 |
| Human activity |  |  |  |  |  |  |  |
| Cropland | 0·7 (0·6, 0·8) | 12·2 (12·1, 12·4) | 4·6 (4·4, 4·7) | 1·2 (1·1, 1·3) | 8·2 (8·1, 8·4) | 0·9 (0·8, 0·9) | 1·1 (1·1, 1·2) |
| Urbanland | 0 | 12·0 (11·8, 12·2) | 0·8 (0·8, 0·9) | 0·5 (0·5, 0·6) | 4·8 (4·7, 4·9) | 0·5 (0·5, 0·6) | 0·8 (0·8, 0·8) |

# Appendix Table S20: Mean (95% percentiles) relative contributions of variables (95% percentiles) of NiV models

| **Variable** | **Spillover model** | **Endemic infection model** |
| --- | --- | --- |
| Animal |  |  |
| *Pteropus lylei* | 0·1 (0·1, 0·1) | 4·9 (4·7, 5·1) |
| *Pteropus medius* | 8·0 (7·2, 8·8) | 14·2 (13·8, 14·6) |
| *Pteropus vampyrus* | 0·1 (0·1, 0·2) | 1·5 (1·4, 1·6) |
| *Pteropus hypomelanus* | 0 | 0·4 (0·4, 0·5) |
| *Rousettus leschenaultii* | 0 | 0·3 (0·3, 0·3) |
| *Rousettus amplexicaudatus* | 0·1 (0·1, 0·1) | 0·3 (0·2, 0·3) |
| *Hipposideros larvatus* | 1·1 (1·0, 1·2) | 3·3 (3·1, 3·5) |
| Mammalian richness | 3·6 (3·2, 4·1) | 6·6 (6·2, 7·1) |
| Chiroptera richness | 0·2 (0·2, 0·3) | 2·9 (2·7, 3·1) |
| Pig density with intensive production | 0·2 (0·1, 0·2) | 0·1 (0·1, 0·2) |
| Environment |  |  |
| Annual mean temperature | 21·6 (20·1, 23·1) | 0·9 (0·8, 1·0) |
| Mean diurnal range | 0·4 (0·3, 0·5) | 1·8 (1·7, 2·0) |
| Isothermality | 0·1 (0·1, 0·1) | 1·9 (1·8, 2·0) |
| Temperature seasonality | 4·2 (3·8, 4·5) | 2·3 (2·2, 2·4) |
| Max temperature of warmest month | 0·1 (0·1, 0·1) | 1·9 (1·7, 2·2) |
| Min temperature of coldest month | 0·5 (0·4, 0·6) | 0·5 (0·4, 0·5) |
| Annual range of temperature | 0·3 (0·3, 0·3) | 0·8 (0·7, 0·8) |
| Mean temperature of wettest quarter | 1·5 (1·3, 1·7) | 6·8 (6·5, 7·1) |
| Mean temperature of driest quarter | 29·6 (29·2, 30) | 15·3 (14·6, 15·9) |
| Mean temperature of warmest quarter | 0·4 (0·3, 0·4) | 8·9 (8·5, 9·3) |
| Mean temperature of coldest quarter | 17·0 (15·5, 18·5) | 2·5 (2·3, 2·6) |
| Annual precipitation | 0·4 (0·3, 0·5) | 1·0 (0·9, 1·0) |
| Precipitation of wettest month | 0·2 (0·1, 0·2) | 0·3 (0·3, 0·3) |
| Precipitation of driest month | 0·5 (0·4, 0·7) | 1·8 (1·7, 1·9) |
| Precipitation seasonality | 2·0 (1·7, 2·2) | 1·5 (1·4, 1·6) |
| Precipitation of wettest quarter | 0·2 (0·2, 0·2) | 0·5 (0·4, 0·5) |
| Precipitation of driest quarter | 0·1 (0·0, 0·1) | 0·7 (0·6, 0·7) |
| Precipitation of warmest quarter | 1·4 (1·3, 1·5) | 3·4 (3·1, 3·6) |
| Precipitation of coldest quarter | 2·9 (2·7, 3·0) | 3·4 (3·2, 3·6) |
| Elevation | 1·1 (0·9, 1·3) | 1·6 (1·5, 1·7) |
| Forest | 0·2 (0·1, 0·2) | 0·3 (0·3, 0·3) |
| Grassland | 0 | 0 |
| Leaf area index | 0·2 (0·2, 0·2) | 0·6 (0·5, 0·7) |
| Mixed cropland and nature vegetation | 0·4 (0·3, 0·4) | 1·4 (1·3, 1·6) |
| Mixed tree, shrub and herbaceous | 0·1 (0·1, 0·1) | 0·5 (0·5, 0·6) |
| Vegetation flooded | 0 | 0·4 (0·3, 0·4) |
| Shrubland | 0·0 (0·0, 0·1) | 0 |
| Sparse vegetation land | 0 | 0 |
| Water body | 0·0 (0·0, 0·1) | 0 (0, 0·1) |
| Bare areas | 0 | 0 (0, 0·1) |
| Date palms (Phoenix sylvestris) | 0·3 (0·2, 0·3) | 0·8 (0·8, 0·9) |
| Human activity |  |  |
| Cropland | 0·1 (0·1, 0·2) | 0·2 (0·2, 0·2) |
| Cropland change | 0·1 (0·1, 0·1) | 0·3 (0·2, 0·3) |
| Urbanland | 0·3 (0·3, 0·3) | 0·5 (0·4, 0·5) |
| Urbanland change | 0·1 (0·0, 0·1) | 0·1 (0·1, 0·1) |
| Gridded population density | 0·1 (0·1, 0·1) | 0·3 (0·3, 0·4) |
| Gridded population change | 0 | 1·2 (1·2, 1·3) |
| GDP change | 0 | 0 |
| GDP | 0 | 0 |
| Human footprint | 0·2 (0·2, 0·2) | 1·1 (1·0, 1·1) |

# Appendix Table S21: Comparison of performance metrics across different cutoff methods for Binary Classification in the NiV endemic infection model using the R “OptimalCutpoints” package.

| **Method** | **cutoff** | **TP** | **FN** | **TN** | **FP** | **Se** | **Sp** | **PPV** | **NPV** | **Po** | **Pe** | **Youden** | **F1** | **Kappa** |
| --- | --- | --- | --- | --- | --- | --- | --- | --- | --- | --- | --- | --- | --- | --- |
| MaxKappa | 0·2853 | 161 | 26 | 1846 | 24 | 0·8610 | 0·9872 | 0·8703 | 0·9861 | 0·9757 | 0·8355 | 0·8481 | 0·9198 | 0·8522 |
| CB | 0·3716 | 156 | 31 | 1852 | 18 | 0·8342 | 0·9904 | 0·8966 | 0·9835 | 0·9762 | 0·8399 | 0·8246 | 0·9056 | 0·8512 |
| MaxEfficiency | 0·3716 | 156 | 31 | 1852 | 18 | 0·8342 | 0·9904 | 0·8966 | 0·9835 | 0·9762 | 0·8399 | 0·8246 | 0·9056 | 0·8512 |
| MinValueSe | 0·3139 | 159 | 28 | 1847 | 23 | 0·8503 | 0·9877 | 0·8736 | 0·9851 | 0·9752 | 0·8367 | 0·8380 | 0·9138 | 0·8482 |
| PrevalenceMatching | 0·2674 | 161 | 26 | 1844 | 26 | 0·861 | 0·9861 | 0·8610 | 0·9861 | 0·9747 | 0·8347 | 0·8471 | 0·9193 | 0·8471 |
| MinValueNPVPPV | 0·2302 | 162 | 25 | 1842 | 28 | 0·8663 | 0·9805 | 0·8526 | 0·9866 | 0·9742 | 0·8335 | 0·8513 | 0·9219 | 0·8452 |
| ValuePPV | 0·2270 | 162 | 25 | 1841 | 29 | 0·8663 | 0·9845 | 0·8482 | 0·9866 | 0·9737 | 0·8331 | 0·8508 | 0·9216 | 0·8427 |
| NPVEqualPPV | 0·6911 | 139 | 48 | 1866 | 4 | 0·7433 | 0·9979 | 0·9720 | 0·9749 | 0·9747 | 0·8522 | 0·7412 | 0·8520 | 0·8289 |
| PROC01 | 0·7320 | 135 | 52 | 1868 | 2 | 0·7219 | 0·9989 | 0·9854 | 0·9729 | 0·9737 | 0·8546 | 0·7209 | 0·8381 | 0·8195 |
| ObservedPrev | 0·0900 | 168 | 19 | 1807 | 63 | 0·8984 | 0·9663 | 0·7273 | 0·9896 | 0·9601 | 0·8172 | 0·8647 | 0·9311 | 0·7819 |
| MeanPrev | 0·0833 | 169 | 18 | 1803 | 67 | 0·9037 | 0·9642 | 0·7161 | 0·9901 | 0·9587 | 0·8152 | 0·8679 | 0·9330 | 0·7764 |
| MaxSpSe | 0·0308 | 175 | 12 | 1746 | 124 | 0·9358 | 0·9337 | 0·5853 | 0·9932 | 0·9339 | 0·7902 | 0·8695 | 0·9348 | 0·6849 |
| ROC01 | 0·0292 | 177 | 10 | 1742 | 128 | 0·9465 | 0·9316 | 0·5803 | 0·9943 | 0·9329 | 0·7878 | 0·8781 | 0·9390 | 0·6839 |
| Youden | 0·0292 | 177 | 10 | 1742 | 128 | 0·9465 | 0·9316 | 0·5803 | 0·9943 | 0·9329 | 0·7878 | 0·8781 | 0·9390 | 0·6839 |
| MinValueSp | 0·0134 | 181 | 6 | 1631 | 239 | 0·9679 | 0·8722 | 0·4310 | 0·9963 | 0·8809 | 0·742 | 0·8401 | 0·9176 | 0·5383 |

**Note**:

TP: Number of True Positives; FN: Number of False Negatives; TN: Number of True Negatives; FP: Number of False Positives;

Se = TP / (TP + FN); Sp = TN / (TN + FP);

PPV = TP / (TP + FP); NPV = TN / (TN + FN);

Po = (TP + FN) / (TP + FN + TN + FP); Pe = (FP + TN) / (TP + FN + TN + FP);

Youden = Se + Sp - 1; F1 = 2 * (PPV * Se) / (PPV + Se); Kappa = (Po - Pe) / (1 - Pe)

Reference: Šimundić AM. Measures of Diagnostic Accuracy: Basic Definitions. *EJIFCC*. 2009; **19**: 203-211.

# Appendix Table S22: The absolute out-of-sample performance between ecological model and sensitivity analysis.

| **Model** | **MAE** | **MSE** | **RMSE** | **R^2^** |
| --- | --- | --- | --- | --- |
| Date palms (*Phoenix sylvestris*) | 0·0172 | 0·0028 | 0·053 | 0·9258 |
| *Pteropus lylei* | 0·0022 | 0·0005 | 0·0215 | 0·9269 |
| *Pteropus medius* | 0·0123 | 0·0014 | 0·0372 | 0·9598 |
| *Pteropus vampyrus* | 0·0114 | 0·0011 | 0·0338 | 0·929 |
| *Pteropus hypomelanus* | 0·0096 | 0·0015 | 0·0382 | 0·8104 |
| *Rousettus leschenaultii* | 0·0214 | 0·0016 | 0·0403 | 0·8558 |
| *Rousettus amplexicaudatus* | 0·0109 | 0·0014 | 0·0376 | 0·8535 |
| *Hipposideros larvatus* | 0·0199 | 0·0015 | 0·0386 | 0·9261 |
| NiV spillover model | 0·0011 | 0·0001 | 0·0094 | 0·9700 |
| NiV endemic infection model | 0·0022 | 0·0002 | 0·0132 | 0·9415 |

**Note**:

MAE: the Mean Average Error

MSE: the Mean Squared Error

RMSE: the Root Mean Squared Error

R^2^: the coefficient of determination

# Appendix Figure S1: Timeline of NiV cases from 1999 to 2021 (A) and radar chart (B) illustrating the monthly cumulative NiV case numbers.

# Appendix Figure S2: Chord diagram representing affected countries and host diversity according to the infectious cases.

# Appendix Figure S3: Geographical distribution of eight bat species linked to NiV infection

**Note:** Multiple methods including PCR/RT-PCR/RT-qPCR/qRT-PCR/virus isolation.

Appendix Figure S4: Root-to-tip plot of the genetic distance against sampling time of the NiV phylogeny. Genetic divergence was based on a ML tree generated using the dataset of 66 NiV complete genomes. The genetic distances from the root to the tips of the ML tree are plotted against the date of isolation. The regression line is plotted according to the best fitting root which minimises the sum of the squared residuals from the regression line. The x-intercept of the regression line represents the tMRCA, while the gradient of the line represents the clock rate. The correlation coefficient (R^2^) estimates the dispersion of the residuals around the regression line. The plot was generated using “TempEst”.

Appendix Figure S5: Bayesian skyline plot of effective population size. The y-axis is the product of effective population size and generation length in a log scale while the x-axis is the time before present. The median estimate (solid dark blue line) and 95% highest probability density (HPD) limits (light blue area) are shown.

# Appendix Figure S6: Case fatality rates of NiV infection based on subgroup meta-analysis by clades.

# Appendix Figure S7: Case fatality rates of NiV infection based on subgroup meta-analysis by countries

# Appendix Figure S8: Case fatality rates of NiV infection based on subgroup meta-analysis by publish year

# Appendix Figure S9: Case fatality rates of NiV infection based on subgroup meta-analysis by mean age

# Appendix Figure S10: Case fatality rates of NiV infection based on subgroup meta-analysis by male proportion

# Appendix Figure S11: Case fatality rates of NiV infection based on subgroup meta-analysis by risk of bias

# Appendix Figure S12: Case fatality rates of NiV infection based on subgroup meta-analysis by GDP per capita

# Appendix Figure S13: Case fatality rates of NiV infection based on subgroup meta-analysis by HDI index

# Appendix Figure S14: Case fatality rates of NiV infection based on subgroup meta-analysis by Gini index

# Appendix Figure S15: Case fatality rates of NiV infection based on subgroup meta-analysis by travel time to healthcare facilities

# Appendix Figure S16: Funnel plot of meta-analysis of CFR (not sub-grouped)

Appendix Figure S17: Funnel plots of subgroup meta-analysis of CFR. (A) subgroup by clade, (B) subgroup by country, (C) subgroup by publish year, (D) subgroup by mean age, (E) subgroup by male proportion, (F) subgroup by risk of bias, (G) subgroup by GDP per capita, (H) subgroup by HDI index, (I) subgroup by Gini index, (J) subgroup by travel time to healthcare facilities.

Appendix Figure S18: Bubble plot with a fitted meta-regression for the variation of the case fatality rate of NiV infection. Meta-regression analysis of CFR according to: (A) mean age (showing a statistically significant downward trend), (B) GDP per capita (showing a statistically significant downward trend), (C) human development index (HDI) (showing a statistically significant downward trend), (D) travel time to healthcare facilities (showing a statistically significant downward trend).

Appendix Figure S19: Meta-analysis of clinical manifestation proportion. (I) fever; (II) headache; (III) chills; (IV) cough; (V) altered consciousness; (VI) drowsiness; (VII) seizure; (VIII) nausea; (IX) vomiting; (X) diarrhea; (XI) dyspnea; (XII) myalgia.

Appendix Figure S20: Funnel plots of clinical subgroup meta-proportion of NiV infections. (A) fever; (B) headache; (C) chills; (D) cough; (E) altered consciousness; (F) drowsiness; (G) seizure; (H) nausea; (I) vomiting; (J) diarrhea; (K) dyspnea; (L) myalgia.

Appendix Figure S21: Positive detection rate of NiV in reservoir hosts based on subgroup meta-analysis.

# Appendix Figure S22: Funnel plot of meta-analysis of NiV detection rate in reservoir hosts (not sub-grouped)

Appendix Figure S23: Funnel plots of subgroup meta-analysis of NiV detection rate in reservoir hosts. (A) subgroup by species, (B) subgroup by country, (C) subgroup by detection method, (D) subgroup by publish year.

Appendix Figure S24: Geographic range and occurrences of *Pteropus lylei* according to IUCN Red List of Threatened Species. The colored polygon represents the residential extent. The red points represent the occurrences of the bat species.

**Reference**:

1. Waldien, D.L. & Tsang, S.M. 2021. *Pteropus lylei*. The IUCN Red List of Threatened Species 2021: e.T18734A22082429. https://dx.doi.org/10.2305/IUCN.UK.2021-1.RLTS.T18734A22082429.en.

2. GBIF.org (09 November 2021) GBIF Occurrence Download https://doi.org/10.15468/dl.csrjza

Appendix Figure S25: Geographic range and occurrences of *Pteropus medius* according to IUCN Red List of Threatened Species. The colored polygon represents the residential extent. The red points represent the occurrences of the bat species.

**Reference**:

1. Tsang, S.M. 2020. *Pteropus giganteus* (errata version published in 2021). The IUCN Red List of Threatened Species 2020: e.T18725A194134899. https://dx.doi.org/10.2305/IUCN.UK.2020-3.RLTS.T18725A194134899.en.

2. GBIF.org (09 November 2021) GBIF Occurrence Download https://doi.org/10.15468/dl.z4y22x

Appendix Figure S26: Geographic range and occurrences of *Pteropus vampyrus* according to IUCN Red List of Threatened Species. The colored polygon represents the residential extent. The red points represent the occurrences of the bat species.

**Reference**:

1. Mildenstein, T., Abdul Aziz, S., Paguntalan, L., et.al. 2022. *Pteropus vampyrus*. The IUCN Red List of Threatened Species 2022: e.T18766A22088824. https://dx.doi.org/10.2305/IUCN.UK.2022-2.RLTS.T18766A22088824.en.

2. GBIF.org (09 November 2021) GBIF Occurrence Download https://doi.org/10.15468/dl.esaysq

Appendix Figure S27: Geographic range and occurrences of *Pteropus hypomelanus* according to IUCN Red List of Threatened Species. The colored polygon represents the residential extent. The red points represent the occurrences of the bat species.

**Reference**:

1. Tsang, S.M. 2020. *Pteropus hypomelanus*. The IUCN Red List of Threatened Species 2020: e.T18729A22081642. https://dx.doi.org/10.2305/IUCN.UK.2020-2.RLTS.T18729A22081642.en.

2. GBIF.org (09 November 2021) GBIF Occurrence Download https://doi.org/10.15468/dl.h8zqkh

Appendix Figure S28: Geographic range and occurrences of *Rousettus leschenaultii* according to IUCN Red List of Threatened Species. The colored polygon represents the residential extent. The red points represent the occurrences of the bat species.

**Reference**:

1. Bouillard, N., Cretan, S. & Waldien, D.L. 2021. *Rousettus leschenaultii*. The IUCN Red List of Threatened Species 2021: e.T19756A22001287. https://dx.doi.org/10.2305/IUCN.UK.2021-3.RLTS.T19756A22001287.en.

2. GBIF.org (09 November 2021) GBIF Occurrence Download https://doi.org/10.15468/dl.sejh75

Appendix Figure S29: Geographic range and occurrences of *Rousettus amplexicaudatus* according to IUCN Red List of Threatened Species. The colored polygon represents the residential extent. The red points represent the occurrences of the bat species.

**Reference**:

1. Waldien, D.L., Wilson, Z., Adleson, S., et.al. 2019. *Rousettus amplexicaudatus*. The IUCN Red List of Threatened Species 2019: e.T19754A22001514. https://dx.doi.org/10.2305/IUCN.UK.2019-3.RLTS.T19754A22001514.en.

2. GBIF.org (09 November 2021) GBIF Occurrence Download https://doi.org/10.15468/dl.7mjcm5

Appendix Figure S30: Geographic range and occurrences of *Hipposideros larvatus* according to IUCN Red List of Threatened Species. The colored polygon represents the residential extent. The red points represent the occurrences of the bat species.

**Reference**:

1. Srinivasulu, C. & Srinivasulu, A. 2020. *Hipposideros larvatus*. The IUCN Red List of Threatened Species 2020: e.T85646564A22091287. https://dx.doi.org/10.2305/IUCN.UK.2020-3.RLTS.T85646564A22091287.en.

2. GBIF.org (09 November 2021) GBIF Occurrence Download https://doi.org/10.15468/dl.b8m2p9

Appendix Figure S31: Geographic range and occurrences of *Pipistrellus pipistrellus* according to IUCN Red List of Threatened Species. The colored polygon represents the residential extent. The red points represent the occurrences of the bat species.

**Reference**:

1. Godlevska, L., Bücs, S., Kruskop, S.V., et.al. 2020. *Pipistrellus pipistrellus* (errata version published in 2021). The IUCN Red List of Threatened Species 2020: e.T85333513A196581936. <https://dx.doi.org/10.2305/IUCN.UK.2020-2.RLTS.T85333513A196581936.en>.

2. GBIF.org (09 November 2021) GBIF Occurrence Download https://doi.org/10.15468/dl.4avgqt

Appendix Figure S32: Palm occurrences used as the background to generate pseudo-absence data in the date palms (*Phoenix sylvestris*) ecological niche models. The occurrence data are from three datasets, including GBIF, iDigBio and iNaturalist.

**Reference**:

GBIF.org (06 January 2024) GBIF Occurrence Download https://doi.org/10.15468/dl.4nkujh

Appendix Figure S33: Bat occurrences used as the background to generate pseudo-absence data in the host ecological niche models. The occurrence data are from five bat datasets, including DarkCideS 1.0, Bat Eco-Interactions, GBIF, iDigBio and iNaturalist.

**Reference**:

GBIF.org (09 November 2021) GBIF Occurrence Download https://doi.org/10.15468/dl.av69xa

Appendix Figure S34: Human virus occurrences used as the background to generate pseudo-absence data in the NiV spillover model. The occurrence data are from two human virus datasets, including Chikungunya virus and Dengue virus.

Appendix Figure S35: Bat-borne virus occurrences used as the background to generate pseudo-absence data in the NiV reservoir host model. The occurrence data are from four bat virus datasets, including Coronavirus (from two bat-coronavirus articles), Global Biotic Interaction (GloBI), PREDICT project in USAID’s Emerging Pandemic Threats program, and PubMed.

Appendix Figure S36: Predicted ecological niche of date palms (*Phoenix sylvestris*). (A) Ecological niche of *Phoenix sylvestris*; (B) Map illustrating the uncertainty of predicted niche of *Phoenix sylvestris* represented by the SD of each pixel. Each pixel represented a 10×10 km area. (C) The relative influence of predictors on occurrence probability. (D) Effect plots of predictors based on BRT models ordered by mean relative contributions, which are presented in parentheses. The mean curves (red) and 95% percentiles (orange/blue) show the influence on predicting the probability of occurrence. The histograms (grey) show the frequency distributions of the predictors. (E) Predictive performance of ecological niches of *Phoenix sylvestris*.

Appendix Figure S37: Predicted ecological niches of NiV reservoir hosts. (A) Study area of study, i.e., India, Pakistan, Bangladesh, Nepal, Bhutan, Sri Lanka, Indonesia, Philippines, Vietnam, Thailand, Myanmar, Malaysia, Cambodia, Lao, Singapore, East Timor, Brunei, China, Australia and Papua New Guinea; (B) ecological niche of *Pteropus lylei*, mainly distributed in Thailand and Cambodia; (C) ecological niche of *Pteropus medius*; mainly distributed in the South Asian subcontinent; (D) ecological niche of *Pteropus vampyrus*, mainly distributed in Malay Archipelago; (E) ecological niche of *Pteropus hypomelanus*, mainly distributed in Malay Archipelago; (F) ecological niche of *Rousettus leschenaultii*; mainly distributed in the South Asian subcontinent and the Indochina Peninsula; (G) ecological niche of *Rousettus amplexicaudatus*; mainly distributed in the Malay Archipelago; (H) ecological niche of *Hipposideros larvatus*, mainly distributed in the Indochina Peninsula and the Malay Archipelago. For both panels, redder colors indicate larger values and bluer colors indicate smaller values.

Appendix Figure S38: Uncertainty maps for ecological niche model of NiV reservoir hosts by the SD of each grid. (A) Map illustrating the uncertainty of ecological niche of *Pteropus lylei*; (B) Map illustrating the uncertainty of ecological niche of *Pteropus medius*; (C) Map illustrating the uncertainty of ecological niche of *Pteropus vampyrus*; (D) Map illustrating the uncertainty of ecological niche of *Pteropus hypomelanus*; (E) Map illustrating the uncertainty of ecological niche of *Rousettus leschenaultii*; (F) Map illustrating the uncertainty of ecological niche of *Rousettus amplexicaudatus*; (G) Map illustrating the uncertainty of ecological niche of *Hipposideros larvatus*. Each pixel represented a 10× 10 km area.

Appendix Figure S39: The relative contribution of top nine predictors and response curve in the BRT models for *Pteropus lylei*. (A) The relative influence of predictors on occurrence probability. (B) Effect plots of predictors based on BRT models ordered by mean relative contributions, which are presented in parentheses. The mean curves (red) and 95% percentiles (orange/blue/green) show the influence on predicting the probability of occurrence. The histograms (grey) show the frequency distributions of the predictors.

Appendix Figure S40: The relative contribution and response curve of top nine predictors in the BRT models for *Pteropus medius*. (A) The relative influence of predictors on occurrence probability. (B) Effect plots of predictors based on BRT models ordered by mean relative contributions, which are presented in parentheses. The mean curves (red) and 95% percentiles (orange/blue/green) show the influence on predicting the probability of occurrence. The histograms (grey) show the frequency distributions of the predictors.

Appendix Figure S41: The relative contribution and response curve of top nine predictors in the BRT models for *Pteropus vampyrus*. (A) The relative influence of predictors on occurrence probability. (B) Effect plots of predictors based on BRT models ordered by mean relative contributions, which are presented in parentheses. The mean curves (red) and 95% percentiles (orange/blue/green) show the influence on predicting the probability of occurrence. The histograms (grey) show the frequency distributions of the predictors.

Appendix Figure S42: The relative contribution and response curve of top nine predictors in the BRT models for *Pteropus hypomelanus*. (A) The relative influence of predictors on occurrence probability. (B) Effect plots of predictors based on BRT models ordered by mean relative contributions, which are presented in parentheses. The mean curves (red) and 95% percentiles (orange/blue) show the influence on predicting the probability of occurrence. The histograms (grey) show the frequency distributions of the predictors.

Appendix Figure S43: The relative contribution and response curve of top nine predictors in the BRT models for *Rousettus leschenaultii*. (A) The relative influence of predictors on occurrence probability. (B) Effect plots of predictors based on BRT models ordered by mean relative contributions, which are presented in parentheses. The mean curves (red) and 95% percentiles (orange/blue/green) show the influence on predicting the probability of occurrence. The histograms (grey) show the frequency distributions of the predictors.

Appendix Figure S44: The relative contribution and response curve of top nine predictors in the BRT models for *Rousettus amplexicaudatus*. (A) The relative influence of predictors on occurrence probability. (B) Effect plots of predictors based on BRT models ordered by mean relative contributions, which are presented in parentheses. The mean curves (red) and 95% percentiles (orange/blue) show the influence on predicting the probability of occurrence. The histograms (grey) show the frequency distributions of the predictors.

Appendix Figure S45: The relative contribution and response curve of top nine predictors in the BRT models for *Hipposideros larvatus*. (A) The relative influence of predictors on occurrence probability. (B) Effect plots of predictors based on BRT models ordered by mean relative contributions, which are presented in parentheses. The mean curves (red) and 95% percentiles (orange/blue) show the influence on predicting the probability of occurrence. The histograms (grey) show the frequency distributions of the predictors.

Appendix Figure S46: The relative contribution and response curve of top nine predictors in the NiV spillover model. (A) The relative influence of predictors on occurrence probability. (B) Effect plots of predictors based on BRT models ordered by mean relative contributions, which are presented in parentheses. The mean curves (red) and 95% percentiles (orange/blue) show the influence on predicting the probability of occurrence. The histograms (grey) show the frequency distributions of the predictors.

Appendix Figure S47: The relative contribution and response curve of top nine predictors in the NiV endemic infection model. (A) The relative influence of predictors on occurrence probability. (B) Effect plots of predictors based on BRT models ordered by mean relative contributions, which are presented in parentheses. The mean curves (red) and 95% percentiles (orange/blue) show the influence on predicting the probability of occurrence. The histograms (grey) show the frequency distributions of the predictors.

Appendix Figure S48: Predictive performance of ecological niches of NiV reservoir hosts. (A) *Pteropus lylei*; (B) *Pteropus medius*; (C) *Pteropus vampyrus*; (D) *Pteropus hypomelanus*; (E) *Rousettus leschenaultii*; (F) *Rousettus amplexicaudatus*; (G) *Hipposideros larvatus*.

Appendix Figure S49: Predictive performance of ecological niches of NiV model. (A) NiV spillover model; (B) NiV endemic infection model.

Appendix Figure S50: Geographic representation of binary risky areas based on NiV endemic infection model. Red represents risky areas, gray represents no risk, and the cutoff value was based on the method of MaxKappa in the R 'OptimalCutpoints' package.

Appendix Figure S51: The sensitivity analysis of ecological niche model. (A) date palms (*Phoenix sylvestris*) (B) *Pteropus lylei*; (C) *Pteropus medius*; (D) *Pteropus vampyrus*; (E) *Pteropus hypomelanus*; (F) *Rousettus leschenaultii*; (G) *Rousettus amplexicaudatus*; (H) *Hipposideros larvatus*; (I) NiV spillover model; (J) NiV endemic infection model.

Appendix Figure S52: Plots of two variables which may be related to NiV transmission. (A) Pig density with intensive production; (B) Probability of *Phoenix sylvestris* occurrence.

# Appendix Figure S53: Plots of main covariates (except for bats) in the BRT model

# Appendix Text S5: References included in the NiV study (Reference ID)

1. CDC. Update: outbreak of Nipah virus-Malaysia and Singapore, 1999. *MMWR Morb Mortal Wkly Rep* 1999; **48**: 335–7.
2. Chua KB, Goh KJ, Wong KT, et al. Fatal encephalitis due to Nipah virus among pig-farmers in Malaysia. *Lancet* 1999; **354**: 1257–9.
3. Kay-Sin TAN MRCP C-TTFM, Khean-Jin GOH MRCP. Epidemiological aspects of Nipah virus infection. 1999. *Neurol J Southeast Asia* 1999; **4**: 77–8.
4. Lee KE, Umapathi T, Tan CB, et al. The neurological manifestations of Nipah virus encephalitis, a novel paramyxovirus. *Ann Neurol* 1999; **46**: 428–32.
5. Paton NI, Leo YS, Zaki SR, et al. Outbreak of Nipah-virus infection among abattoir workers in Singapore. *Lancet* 1999; **354**: 1253–6.
6. Ahmad K. Malaysia culls pigs as Nipah virus strikes again. *Lancet* 2000; **356**: 230.
7. Amal NM, Lye MS, Ksiazek TG, et al. Risk factors for Nipah virus transmission, Port Dickson, Negeri Sembilan, Malaysia: results from a hospital-based case-control study. *Southeast Asian J Trop Med Public Health* 2000; **31**: 301–6.
8. Chua KB, Lam SK, Tan CT, et al. High mortality in Nipah encephalitis is associated with presence of virus in cerebrospinal fluid. *Ann Neurol* 2000; **48**: 802–5.
9. Goh KJ, Tan CT, Chew NK, et al. Clinical features of Nipah virus encephalitis among pig farmers in Malaysia. *N Engl J Med* 2000; **342**: 1229–35.
10. Parashar UD, Sunn LM, Ong F, et al. Case-control study of risk factors for human infection with a new zoonotic paramyxovirus, Nipah virus, during a 1998–1999 outbreak of severe encephalitis in Malaysia. *J Infect Dis* 2000; **181**: 1755–9.
11. Ali R, Mounts AW, Parashar UD, et al. Nipah virus among military personnel involved in pig culling during an outbreak of encephalitis in Malaysia, 1998–1999. *Emerg Infect Dis* 2001; **7**: 759–61.
12. Chua KB, Lam SK, Goh KJ, et al. The presence of Nipah virus in respiratory secretions and urine of patients during an outbreak of Nipah virus encephalitis in Malaysia. *J Infect* 2001; **42**: 40–3.
13. Mounts AW, Kaur H, Parashar UD, et al. A cohort study of health care workers to assess nosocomial transmissibility of Nipah virus, Malaysia, 1999. J *Infect Dis* 2001; **183**: 810–3.
14. Sahani M, Parashar UD, Ali R, et al. Nipah virus infection among abattoir workers in Malaysia, 1998–1999. *Int J Epidemiol* 2001; **30**: 1017–20.
15. Tambyah PA, Tan JH, Ong BK, Ho KH, Chan KP. First case of Nipah virus encephalitis in Singapore. *Intern Med J* 2001; **31**: 132–3.
16. Wong SC, Ooi MH, Wong MN, Tio PH, Solomon T, Cardosa MJ. Late presentation of Nipah virus encephalitis and kinetics of the humoral immune response. *J Neurol Neurosurg Psychiatry* 2001; **71**: 552–4.
17. Yob JM, Field H, Rashdi AM, et al. Nipah virus infection in bats (order Chiroptera) in peninsular Malaysia. *Emerg Infect Dis* 2001; **7**: 439–41.
18. Chan KP, Rollin PE, Ksiazek TG, et al. A survey of Nipah virus infection among various risk groups in Singapore. *Epidemiol Infect* 2002; **128**: 93–8.
19. Chong HT, Kunjapan SR, Thayaparan T, et al. Nipah encephalitis outbreak in Malaysia, clinical features in patients from Seremban. *Can J Neurol Sci* 2002; **29**: 83–7.
20. Chua KB, Koh CL, Hooi PS, et al. Isolation of Nipah virus from Malaysian Island flying-foxes. *Microbes Infect* 2002; **4**: 145–51.
21. Lim CC, Lee KE, Lee WL, et al. Nipah virus encephalitis: serial MR study of an emerging disease. *Radiol* 2002; **222**: 219–26.
22. Tan CT, Goh KJ, Wong KT, et al. Relapsed and late-onset Nipah encephalitis. *Ann Neurol* 2002; **51**: 703–8.
23. Wong KT, Shieh WJ, Kumar S, et al. Nipah virus infection: pathology and pathogenesis of an emerging paramyxoviral zoonosis. *Am J Pathol* 2002; **161**: 2153–67.
24. FAO/WHO. Japanese encephalitis/Nipah outbreak in Malaysia. *Global Forum of Food Safety Regulators Marrakech*. Morocco; 2002.
25. Sim BF, Jusoh MR, Chang CC, Khalid R. Nipah Encephalitis: A report of 18 patients from Kuala Lumpur Hospital. *Neurol J Southeast Asia* 2002; **7**: 13–18.
26. ICDDR B. Outbreaks of encephalitis due to Nipah/Hendra-like viruses, Western Bangladesh. *Health and Science Bulletin (HSB)* 2003; **1**: 1–6.
27. Lim CC, Lee WL, Leo YS, et al. Late clinical and magnetic resonance imaging follow up of Nipah virus infection. *J Neurol Neurosurg Psychiatry* 2003; **74**: 131–3.
28. Hsu VP, Hossain MJ, Parashar UD, et al. Nipah virus encephalitis reemergence, Bangladesh. *Emerg Infect Dis* 2004; **10**: 2082–7.
29. WHO. Nipah virus outbreaks in Bangladesh, January-April 2004. 2004. https://www.who.int/publications/i/item/WER7917 (accessed Dec 16 2022).
30. WHO. Nipah-like virus in Bangladesh. 2004. https://www.who.int/emergencies/disease-outbreak-news/item/2004_02_12-en (accessed Dec 16 2022).
31. WHO. Outbreak news: Dengue fever, Nipah-like fever in Banglades, Avian influenza, influenza and international health regulations. 2004. https://www.who.int/publications/i/item/who-wer7910 (accessed Dec 16 2022).
32. AbuBakar S, Chang LY, Ali AR, Sharifah SH, Yusoff K, Zamrod Z. Isolation and molecular identification of Nipah virus from pigs. *Emerg Infect Dis* 2004; **10**: 2228–30.
33. ICDDR B. Nipah virus outbreak from date palm juice. *Health and Science Bulletin (HSB)* 2005; **3**: 1–5.
34. Reynes JM, Counor D, Ong S, et al. Nipah virus in Lyle's flying foxes, Cambodia. *Emerg Infect Dis* 2005; **11**: 1042–7.
35. Wacharapluesadee S, Lumlertdacha B, Boongird K, et al. Bat Nipah virus, Thailand. *Emerg Infect Dis* 2005; **11**: 1949–51.
36. Chadha MS, Comer JA, Lowe L, et al. Nipah virus-associated encephalitis outbreak, Siliguri, India. *Emerg Infect Dis* 2006; **12**: 235–40.
37. Epstein JH, Rahman SA, Zambriski JA, et al. Feral cats and risk for Nipah virus transmission. *Emerg Infect Dis* 2006; **12**: 1178–9.
38. Luby SP, Rahman M, Hossain MJ, et al. Foodborne transmission of Nipah virus, Bangladesh. *Emerg Infect Dis* 2006; **12**: 1888–94.
39. Gurley ES, Montgomery JM, Hossain MJ, et al. Person-to-person transmission of Nipah virus in a Bangladeshi community. *Emerg Infect Dis* 2007; **13**: 1031–7.
40. Gurley ES, Montgomery JM, Hossain MJ, et al. Risk of nosocomial transmission of Nipah virus in a Bangladesh hospital. *Infect Control Hosp Epidemiol* 2007; **28**: 740–2.
41. Homira N, Rahman M, Hossain MJ, et al. Nipah outbreak with person-to-person transmission in Bangladesh, 2007. *Am J Trop Med Hyg* 2007; **77**: 80.
42. Sejvar JJ, Hossain J, Saha SK, et al. Long-term neurological and functional outcome in Nipah virus infection. i2007; **62**: 235–42.
43. Shirai J, Sohayati AL, Mohamed Ali AL, Suriani MN, Taniguchi T, Sharifah SH. Nipah virus survey of flying foxes in Malaysia. Jarq-Japan Agricultural Research Quarterly 2007; **41**: 69–78.
44. IEDCR. Outbreak list of 2007. 2007. https://old.iedcr.gov.bd/website/index.php/outbreak/85-outbreak-list-of-2007 (accessed Dec 16 2022).
45. Homaira N, Rahman M, Hossain MJ, et al. Evidence of person-to-person transmission of Nipah virus through casual contact. *Int J Infect Dis* 2008; **12**: E99.
46. Hossain MJ, Gurley ES, Montgomery JM, et al. Clinical presentation of Nipah virus infection in Bangladesh. *Clin Infect Dis* 2008; **46**: 977–84.
47. Montgomery JM, Hossain MJ, Gurley E, et al. Risk factors for Nipah virus encephalitis in Bangladesh. *Emerg Infect Dis* 2008; **14**: 1526–32.
48. IEDCR. List of outbreak investigations in 2008. 2008. https://old.iedcr.gov.bd/website/index.php/outbreak/86-list-of-outbreak-investigations-in-2008 (accessed Dec 16 2022).
49. Luby SP, Hossain MJ, Gurley ES, et al. Recurrent zoonotic transmission of Nipah virus into humans, Bangladesh, 2001-2007. *Emerg Infect Dis* 2009; **15**: 1229–35.
50. Umapathi T, Lim CCT. Progressive upper limb weakness - a delayed effect of Nipah virus infection? *J Neurol Sci* 2009; **285**: S188.
51. Chua KB. Epidemiology, surveillance and control of Nipah virus infections in Malaysia. *Malays J Pathol* 2010; **32**: 69–73.
52. Homaira N, Rahman M, Hossain MJ, et al. Nipah virus outbreak with person-to-person transmission in a district of Bangladesh, 2007. *Epidemiol Infect* 2010; **138**: 1630–6.
53. Homaira N, Rahman M, Hossain MJ, et al. Cluster of Nipah virus infection, Kushtia District, Bangladesh, 2007. *PloS One* 2010; **5**: e13570.
54. ICDDR B. Nipah outbreak in Faridpur District, Bangladesh, 2010. *Health Science Bulletin (HSB)* 2010; **8**: 6–11.
55. Rahman SA, Hassan SS, Olival KJ, et al. Characterization of Nipah virus from naturally infected *Pteropus vampyrus* bats, Malaysia. *Emerg Infect Dis* 2010; **16**: 1990–3.
56. Wacharapluesadee S, Boongird K, Wanghongsa S, et al. A longitudinal study of the prevalence of Nipah virus in *Pteropus lylei* bats in Thailand: evidence for seasonal preference in disease transmission. *Vector Borne Zoonotic Dis* 2010; **10**: 183–90.
57. IEDCR. List of outbreak investigations in 2010 (Updated 31 December 2010). 2010. https://old.iedcr.gov.bd/website/index.php/outbreak/88-list-of-outbreak-investigations-in-2010-updated-31-december-2010 (accessed Dec 16 2022).
58. Arankalle VA, Bandyopadhyay BT, Ramdasi AY, et al. Genomic characterization of Nipah virus, West Bengal, India. *Emerg Infect Dis* 2011; **17**: 907–9.
59. ICDDR B. Nipah outbreak in Lalmonirhat district, 2011. *Health Science Bulletin (HSB)* 2011; **9**: 13–18.
60. Sohayati AR, Hassan L, Sharifah SH, et al. Evidence for Nipah virus recrudescence and serological patterns of captive *Pteropus vampyrus*. *Epidemiol Infect* 2011; **139**: 1570–9.
61. Stone R. Breaking the chain in Bangladesh. *Science* 2011; **331**: 1128–31.
62. IEDCR. List of outbreak investigations in 2011 (Updated 07 May 2012). 2011. https://old.iedcr.gov.bd/website/index.php/outbreak/90-list-of-outbreak-investigations-in-2011-updated-07-may-2012 (accessed Dec 16 2022).
63. Abdullah S, Chang L-Y, Rahmat K, Goh KJ, Tan CT. Late-onset Nipah virus encephalitis 11 years after the initial outbreak: A case report. *Neurol Asia* 2012; **17**: 71–4.
64. ICDDR B. Nipah transmission from bats to humans associated with drinking traditional liquor (tari) in northern Bangladesh, 2011. *Health Science Bulletin (HSB)* 2012; **10**: 16–20.
65. Lo MK, Lowe L, Hummel KB, et al. Characterization of Nipah virus from outbreaks in Bangladesh, 2008–2010. *Emerg Infect Dis* 2012; **18**: 248–55.
66. Rahman MA, Hossain MJ, Sultana S, et al. Date palm sap linked to Nipah virus outbreak in Bangladesh, 2008. *Vector Borne Zoonotic Dis* 2012; **12**: 65–72.
67. Yadav PD, Raut CG, Shete AM, et al. Detection of Nipah virus RNA in fruit bat (*Pteropus giganteus*) from India. *Am J Trop Med Hyg* 2012; **87**: 576–8.
68. IEDCR. List of outbreak investigations in 2012 (Updated 01 Jan 2013). 2012. https://old.iedcr.gov.bd/website/index.php/outbreak/118-outbreak.
69. Breed AC, Meers J, Sendow I, et al. The distribution of henipaviruses in Southeast Asia and Australasia: Is wallace's line a barrier to Nipah virus? *PloS One* 2013; **8**: e61316.
70. Sazzad HMS, Hossain MJ, Gurley ES, et al. Nipah virus infection outbreak with nosocomial and corpse-to-human transmission, Bangladesh. Emerg Infect Dis 2013; **19**: 210–7.
71. Sendow I, Ratnawati A, Taylor T, et al. Nipah virus in the fruit bat *Pteropus vampyrus* in Sumatera, Indonesia. *PLoS One* 2013; **8**: e69544.
72. Wacharapluesadee S, Ngamprasertwong T, Kaewpom T, et al. Genetic characterization of Nipah virus from Thai fruit bats (*Pteropus lylei*). *Asian Biomed* 2013; **7**: 813–9.
73. IEDCR. List of outbreak investigations done by IEDCR in 2013. 2013. https://old.iedcr.gov.bd/website/index.php/outbreak/140-outbreak (accessed Dec 16 2022).
74. IEDCR. List of outbreak investigations done by IEDCR in 2014. 2014. https://old.iedcr.gov.bd/website/index.php/outbreak/151-outbreak (accessed Dec 16 2022).
75. IEDCR. Nipah situation in 2014. 2014. https://old.iedcr.gov.bd/website/pdf/files/nipah/Nipah%20Infection_27.02.2014.pdf (accessed Dec 16 2022).
76. Hahn MB, Gurley ES, Epstein JH, et al. The role of landscape composition and configuration on *Pteropus giganteus* roosting ecology and Nipah virus spillover risk in Bangladesh. *Am J Trop Med Hyg* 2014; **90**: 247–255.
77. Ching PK, de los Reyes VC, Sucaldito MN, et al. Outbreak of *Henipavirus* infection, Philippines, 2014. *Emerg Infect Dis* 2015; **21**: 328–31.
78. Naser AM, Hossain MJ, Sazzad HM, et al. Integrated cluster- and case-based surveillance for detecting stage III zoonotic pathogens: an example of Nipah virus surveillance in Bangladesh. *Epidemiol Infect* 2015; **143**: 1922–30.
79. Sazzad HMS, Luby SP, Stroeher U, et al. Exposure-based screening for Nipah virus encephalitis, Bangladesh. *Emerg Infect Dis* 2015; **21**: 349–51.
80. IEDCR. List of outbreak investigations done by IEDCR in 2015. 2015. https://old.iedcr.gov.bd/website/index.php/outbreak/27-outbreak (accessed Dec 16 2022).
81. IEDCR. Nipah infection in 2015. 2015. https://old.iedcr.gov.bd/website/pdf/files/nipah/Nipah%20Infection%20in%202015_Website%20update%20Feb%204%202015_for%20website.pdf (accessed Dec 16 2022).
82. Chakraborty A, Sazzad HMS, Hossain MJ, et al. Evolving epidemiology of Nipah virus infection in Bangladesh: evidence from outbreaks during 2010–2011. *Epidemiol Infect* 2016; **144**: 371–80.
83. Epstein JH, Anthony SJ, Islam A, et al. Nipah virus ecology and infection dynamics in its bat reservoir, *Pteropus medius*, in Bangladesh. *Int J Infect Dis* 2016; **53**: 20–1.
84. Hegde ST, Sazzad HM, Hossain MJ, et al. Investigating rare risk factors for Nipah virus in Bangladesh: 2001–2012. *Ecohealth* 2016; **13**: 720–8.
85. Islam MS, Sazzad HM, Satter SM, et al. Nipah virus transmission from bats to humans associated with drinking traditional liquor made from date palm sap, Bangladesh, 2011–2014. *Emerg Infect Dis* 2016; **22**: 664–70.
86. Wacharapluesadee S, Samseeneam P, Phermpool M, et al. Molecular characterization of Nipah virus from *Pteropus hypomelanus* in Southern Thailand. *Virol J* 2016; **13**: 53.
87. Ajith Kumar AK, Anoop Kumar AS. Deadly Nipah outbreak in Kerala: Lessons learned for the future. *Indian J Crit Care Med* 2018; **22**: 475–6.
88. Hassan MZ, Sazzad HMS, Luby SP, et al. Nipah virus contamination of hospital surfaces during outbreaks, Bangladesh, 2013–2014. *Emerg Infect Dis* 2018; **24**: 15–21.
89. Sadanadan R, Arunkumar G, Laserson KF, et al. Towards global health security: response to the May 2018 Nipah virus outbreak linked to Pteropus bats in Kerala, India. *BMJ Glob Health* 2018; **3**: e001086.
90. Swathy SS, Sidharthan M, Issudeen M, Shibukumar TM, Kumar A, Tharayil HM. Psychological interventions during Nipah viral outbreak in Kozhikode District, 2018. *Indian J Psychol Med* 2018; **40**: 387–9.
91. Thulaseedaran NK, Kumar KGS, Kumar J, et al. A case series on the recent Nipah epidemic in Kerala. *J Assoc Physicians India* 2018; **66**: 63–7.
92. Yadav P, Sudeep A, Gokhale M, et al. Circulation of Nipah virus in *Pteropus giganteus* bats in northeast region of India, 2015. *Indian J Med Res* 2018; **147**: 318–20.
93. WHO. Nipah virus in India, 2018. 2018. https://www.who.int/emergencies/disease-outbreak-news/item/31-may-2018-nipah-virus-india-en (accessed Dec 16 2022).
94. Anam AM, Ahmad J, Huq SMR, Rabbani R. Nipah virus encephalitis: MRI findings. *J Roy Coll Physic Edinburgh* 2019; **49**: 227–8.
95. Anderson DE, Islam A, Crameri G, et al. Isolation and full-genome characterization of Nipah viruses from bats, Bangladesh. *Emerg Infect Dis* 2019; **25**: 166–70.
96. Arunkumar G, Abdulmajeed J, Santhosha D, et al. Persistence of Nipah virus RNA in semen of survivor. *Clin Infect Dis* 2019; **69**: 377–8.
97. Arunkumar G, Chandni R, Mourya DT, et al. Outbreak investigation of Nipah virus disease in Kerala, India, 2018. *J Infect Dis* 2019; **219**: 1867–78.
98. Mourya DT, Yadav P, Sudeep AB, et al. Spatial association between a Nipah virus outbreak in India and Nipah virus infection in Pteropus bats. *Clin Infect Dis* 2019; **69**: 378–9.
99. Thomas B, Chandran P, Lilabi MP, et al. Nipah virus infection in Kozhikode, Kerala, South India, in 2018: Epidemiology of an outbreak of an emerging disease. *Indian J Community Med* 2019; **44**: 383–7.
100. Yadav PD, Shete AM, Kumar GA, et al. Nipah virus sequences from humans and bats during Nipah outbreak, Kerala, India, 2018. *Emerg Infect Dis* 2019; **25**: 1003–6.
101. Chandni R, Renjith TP, Fazal A, et al. Clinical manifestations of Nipah virus-infected patients who presented to the emergency department during an outbreak in Kerala state in India, May 2018. *Clin Infect Dis* 2020; **71**: 152–7.
102. Duong V, Cappelle J, Hul V, et al. Circulation of Nipah virus at the human-flying fox interface in Cambodia. *Int J Infect Dis* 2020; **101**: 223.
103. Epstein JH, Anthony SJ, Islam A, et al. Nipah virus dynamics in bats and implications for spillover to humans. *Proc Natl Acad Sci USA* 2020; **117**: 29190–201.
104. Lee KH, Nikolay B, Sazzad HMS, et al. Changing contact patterns over disease progression: Nipah virus as a case study. *J Infect Dis* 2020; **222**: 438–42.
105. Saha R. MS, Halder S, Deb J., Patra A. A clinico-epidemiological study of the first outbreak of Nipah virus in India – report from ground zero. *Int J Med Res Rev* 2020; **8**: 252–8.
106. Sahay RR, Yadav PD, Gupta N, et al. Experiential learnings from the Nipah virus outbreaks in Kerala towards containment of infectious public health emergencies in India. *Epidemiol Infect* 2020; **148**: e90.
107. Tan CT, Wong KT. Infections among Contacts of Patients with Nipah Virus, India. *Emerg Infect Dis* 2020; **26**: 1963.
108. Cappelle J, Hoem T, Hul V, et al. Nipah virus circulation at human-bat interfaces, Cambodia. *Bull World Health Organ* 2020; **98**: 539–47.
109. Gokhale MD, Sreelekshmy M, Sudeep AB, et al. Detection of possible Nipah virus infection in *Rousettus leschenaultii* and *Pipistrellus Pipistrellus* bats in Maharashtra, India. *J Infect Public Health* 2021; **14**: 1010–2.
110. Md Azizul Haque MHR. Distinct MRI findings of Nipah encephalitis: Report of two cases. *TAJ* 2021; **34**: 106–8.
111. Rahman MZ, Islam MM, Hossain ME, et al. Genetic diversity of Nipah virus in Bangladesh. *Int J Infect Dis* 2021; **102**: 144–51.
112. Rajeevan K, Sathi PP, Prasannan K, Jithin RG, Anjana AM. Nipah virus infection: Autopsy of a clinical challenge. *Indian J Pathol Microbiol* 2021; **64**: 621–3.
113. Sudeep AB, Yadav PD, Gokhale MD, et al. Detection of Nipah virus in *Pteropus medius* in 2019 outbreak from Ernakulam district, Kerala, *India. BMC Infect Dis* 2021; **21**: 162.
114. Wacharapluesadee S, Ghai S, Duengkae P, et al. Two decades of one health surveillance of Nipah virus in Thailand. *One Health Outlook* 2021; **3**: 12.
115. IEDCR. Yearly distribution of Nipah cases in Bangladesh, 2001–2021. 2022. https://iedcr.gov.bd/surveillances/93c87e70-9c22-4f21-9506-a1161ecf404f (accessed Dec 16 2022).
116. WHO. Nipah virus disease - India. 2021. https://www.who.int/emergencies/disease-outbreak-news/item/nipah-virus-disease---India (accessed Dec 16 2022).
117. Yadav PD, Sahay RR, Balakrishnan A, et al. Nipah Virus Outbreak in Kerala State, India Amidst of COVID-19 Pandemic. *Front Public Health* 2022; **10**: 818545.
118. McKee CD, Islam A, Rahman MZ, et al. Nipah Virus Detection at Bat Roosts after Spillover Events, Bangladesh, 2012–2019. *Emerg Infect Dis* 2022; **28**: 1384–1392.
119. Alam MGS, Billah MM, Sharif AR, et al. Secondary human-to-human transmission of Nipah virus in an ambulance, Northwestern Bangladesh, February 2019. *OSIR* 2022; **15**: 84–90.

# Appendix Text S6: References searched out in PubMed for the distribution of bat reservoirs

1. Chaiyes A, Duengkae P, Suksavate W, et al. Mapping risk of Nipah virus transmission from bats to humans in Thailand. *Ecohealth* 2022; **19**: 175–189.
2. Schloesing E, Chambon R, Tran A, et al. Patterns of foraging activity and fidelity in a southeast Asian flying fox. *Mov Ecol* 2020; **8**: 46.
3. Inagaki T, Yamada S, Fujii H, et al. Characterization of a novel alphaherpesvirus isolated from the fruit bat *Pteropus lylei* in Vietnam. *J Virol* 2020; **94**: e00673–20.
4. Hengjan Y, Sae-Koo N, Phichitrasilp T, et al. Seasonal variation in the number of deaths in *Pteropus lylei* at Wat Pho Bang Khla temple, Thailand. *J Vet Med Sci* 2018; **80**: 1364–1367.
5. Wacharapluesadee S, Duengkae P, Chaiyes A, et al. Longitudinal study of age-specific pattern of coronavirus infection in Lyle's flying fox (*Pteropus lylei*) in Thailand. *Virol J* 2018; **15**: 38
6. Olival KJ, Dick CW, Simmons NB, et al. Lack of population genetic structure and host specificity in the bat fly, *Cyclopodia horsfieldi*, across species of Pteropus bats in Southeast Asia. *Parasit Vectors* 2013; **6**: 231.
7. Olival KJ, Stiner EO, Perkins SL. Detection of *Hepatocystis* sp. in southeast Asian flying foxes (Pteropodidae) using microscopic and molecular methods. *J Parasitol* 2007; **93**: 1538–1540.
8. Gokhale M, Sudeep AB, Mathapati B, et al. Serosurvey for Nipah virus in bat population of southern part of India. *Comp Immunol Microbiol Infect Dis* 2022; **85**: 101800.
9. Sudeep AB, Yadav PD, Gokhale MD, et al. Detection of Nipah virus in *Pteropus medius* in 2019 outbreak from Ernakulam district, Kerala, India. *BMC Infect Dis* 2021; **21**: 162.
10. Khan W, Nisa NN, Khan AR, et al. Roosting ecology and morphometric analysis of *Pteropus medius* (Indian flying fox) in Lower Dir, district, Pakistan. *Braz J Biol* 2021; **81**: 77–82.
11. Islam A, Hossain ME, Rostal MK, et al. Epidemiology and molecular characterization of rotavirus A in fruit bats in Bangladesh. *Ecohealth* 2020; **17**: 398–405.
12. Olival KJ, Latinne A, Islam A, et al. Population genetics of fruit bat reservoir informs the dynamics, distribution and diversity of Nipah virus. *Mol Ecol* 2020; **29**: 970–985.
13. Kudagammana HDWS, Thevanesam V, Chu DKW, Eriyagama NB, Peiris JSM, Noordeen F. Coronaviruses in guano from *Pteropus medius* bats in Peradeniya, Sri Lanka. *Transbound Emerg Dis* 2018; **65**: 1122–1124.
14. Karawita AC, Himsworth CG, Rajapakse RP, Bollinger TK, de S Gunawardena P. Toxocara pteropodis in free-ranging Indian flying foxes (*Pteropus medius*) in Sri Lanka. *J Wildl Dis* 2017; **53**: 414–416.
15. Ch'ng L, Tsang SM, Ong ZA, et al. Co-circulation of alpha- and beta- coronaviruses in *Pteropus vampyrus* flying foxes from Indonesia. *Transbound Emerg Dis* 2022; **69**: 3917-3925.
16. Iida A, Takemae H, Tarigan R, et al. Viral-derived DNA invasion and individual variation in an Indonesian population of large flying fox *Pteropus vampyrus*. *J Vet Med Sci* 2021; **83**: 1068–1074.
17. Khan W, Nisa NN, Ullah S, et al. Morphology and ectoparasite spectrum of *Pteropus vampyrus* (the flying fox) in different parts of Malakand Division, Pakistan. *Braz J Biol* 2020; **80**: 523–528.
18. Hengjan Y, Saputra V, Mirsageri M, et al. Nighttime behavioral study of flying foxes on the southern coast of West Java, Indonesia. *J Vet Med Sci* 2018; **80**: 1146–1152.
19. Hengjan Y, Pramono D, Takemae H, et al. Daytime behavior of *Pteropus vampyrus* in a natural habitat: the driver of viral transmission. *J Vet Med Sci* 2017; **79**: 1125–1133.
20. Rahman SA, Hassan L, Epstein JH, et al. Risk factors for Nipah virus infection among Pteropid bats, Peninsular Malaysia. *Emerg Infect Dis* 2013; **19**: 51–60.
21. Sendow I, Field HE, Adjid A, et al. Screening for Nipah virus infection in West Kalimantan province, Indonesia. *Zoonoses Public Health* 2010; **57**: 499–503.
22. Breed AC, Field HE, Smith CS, Edmonston J, Meers J. Bats without borders: long-distance movements and implications for disease risk management. *Ecohealth* 2010; **7**: 204–212.
23. Perera T, Schwarz F, Muzeniek T, et al. First complete cytochrome B sequences and molecular taxonomy of bat species from Sri Lanka. *Animals (Basel)* 2022; **12**: 1674.
24. Muzeniek T, Perera T, Siriwardana S, et al. Detection of alpha- and betacoronaviruses in *Miniopterus fuliginosus* and *Rousettus leschenaultii*, two species of Sri Lankan Bats. *Vaccines (Basel)* 2021; **9**: 650.
25. Murugavel B, Kelber A, Somanathan H. Light, flight and the night: effect of ambient light and moon phase on flight activity of pteropodid bats. *J Comp Physiol Neuroethol Sens Neural Behav Physiol* 2021; **207**: 59–68.
26. Raut CG, Yadav PD, Towner JS, et al. Isolation of a novel adenovirus from *Rousettus leschenaultii* bats from India. *Intervirology* 2012; **55**: 488–490.
27. Banskar S, Bhute SS, Suryavanshi MV, Punekar S, Shouche YS. Microbiome analysis reveals the abundance of bacterial pathogens in *Rousettus leschenaultii* guano. *Sci Rep* 2016; **6**: 36948.
28. Dovih P, Laing ED, Chen Y, et al. Filovirus-reactive antibodies in humans and bats in Northeast India imply zoonotic spillover. *PLoS Negl Trop Dis* 2019; **13**: e0007733.
29. Zhao S, Yang M, Liu G, et al. Rickettsiae in the common pipistrelle *Pipistrellus pipistrellus* (Chiroptera: Vespertilionidae) and the bat soft tick *Argas vespertilionis* (Ixodida: Argasidae). *Parasit Vectors* 2020; **13**: 10.
30. Liu X, Yan B, Wang Q, et al. Babesia vesperuginis in common Pipistrelle (*Pipistrellus pipistrellus*) and the bat soft tick *Argas vespertilionis* in the People's Republic of China. *J Wildl Dis* 2018; **54**: 419–421.
31. Cappelle J, Furey N, Hoem T, et al. Longitudinal monitoring in Cambodia suggests higher circulation of alpha and betacoronaviruses in juvenile and immature bats of three species. *Sci Rep* 2021; **11**: 24145.
32. Meng X, Liu T, Zhang L, Jin L, Sun K, Feng J. Effects of colonization, geography and environment on genetic divergence in the intermediate leaf-nosed bat, *Hipposideros larvatus*. *Animals (Basel)* 2021; **11**: 733.
33. Arnuphapprasert A, Riana E, Ngamprasertwong T, et al. First molecular investigation of haemosporidian parasites in Thai bat species. *Int J Parasitol Parasites Wildl* 2020; **13**: 51–61.
34. Rajasegaran P, Shazali N, Khan FAA. Microclimate and physiological effects in the roosts of cave dwelling bats: implications in roost selection and conservation in Sarawak, Malaysian Borneo. *Zoolog Sci* 2018; **35**: 521–527.
35. Vongphayloth K, Douangboubpha B, Sanamxay D, et al. New locality records of *Ixodes granulatus* and *Ixodes vespertilionis* (Acari: Ixodidae) from tree-shrews (Scandentia: Tupaiidae) and bats (Chiroptera: Hipposideridae) in Laos*. J Med Entomol* 2018; **55**: 1035–1039.
36. Lim T, Cappelle J, Hoem T, Furey N. Insectivorous bat reproduction and human cave visitation in Cambodia: A perfect conservation storm? *PLoS One* 2018; **13**: e0196554.
37. Chen Y, Liu Q, Su Q, et al. 'Compromise' in echolocation calls between different colonies of the intermediate leaf-nosed bat (*Hipposideros larvatus*). *PLoS One* 2016; **11**: e0151382.
38. Leong WJ, Quek XF, Tan HY, et al. Seroprevalence of Pteropine orthoreovirus in humans remain similar after nearly two decades (2001–2002 vs. 2017) in Tioman Island, Malaysia. *J Med Virol* 2022; **94**: 771–775.
39. Hou SL, Koh FX, Nuryana I, Sitam FT, Tay ST. Molecular detection of *Bartonella* spp. in Malaysian small flying foxes (*Pteropus hypomelanus*). *Trop Biomed* 2018; **35**: 293–299.
40. Aziz SA, Clements GR, McConkey KR, et al. Pollination by the locally endangered island flying fox (*Pteropus hypomelanus*) enhances fruit production of the economically important durian (Durio zibethinus). *Ecol Evol* 2017; **7**: 8670–8684.
41. Javati S, Guernier-Cambert V, Jonduo M, et al. Diversity of *Leptospira* spp. in bats and rodents from Papua New Guinea. *Transbound Emerg Dis* 2022; 10.1111/tbed.14725.
42. Jahari PNS, Mohd Azman S, Munian K, et al. The first complete mitochondrial genome data of Geoffroy's rousette, *Rousettus amplexicaudatus* originating from Malaysia. *Mitochondrial DNA B Resour* 2020; **5**: 3262–3264.
43. Gunawan G, Saragih GR, Umardani Y, Karnati S, Wihadmadyatami H, Kusindarta DL. Morphological study of the lingual papillae in the fruit bat (*Rousettus amplexicaudatus*) by scanning electron microscopy and light microscopy. *Anat Histol Embryol* 2020; **49**: 173–183.
44. Mendoza RVD, Fontanilla IKC. Whole mitochondrial genome of a Geoffroy's Rousette, *Rousettus amplexicaudatus* (Pteropodidae). *Mitochondrial DNA B Resour* 2019; **4**: 3546–3548.
45. Arai S, Taniguchi S, Aoki K, et al. Molecular phylogeny of a genetically divergent hantavirus harbored by the Geoffroy's rousette (*Rousettus amplexicaudatus*), a frugivorous bat species in the Philippines. *Infect Genet Evol* 2016; **45**: 26–32.
46. Hatta Y, Omatsu T, Tsuchiaka S, et al. Detection of *Campylobacter jejuni* in rectal swab samples from *Rousettus amplexicaudatus* in the Philippines. *J Vet Med Sci* 2016; **78**: 1347–1350.
47. Murakoshi F, Recuenco FC, Omatsu T, et al. Detection and molecular characterization of Cryptosporidium and Eimeria species in Philippine bats. *Parasitol Res* 2016; **115**: 1863–1869.
48. Heideman PD, Utzurrum RC. Seasonality and synchrony of reproduction in three species of nectarivorous Philippines bats. *BMC Ecol* 2003; **3**: 11.
49. Sasaki M, Setiyono A, Handharyani E, et al. Molecular detection of a novel paramyxovirus in fruit bats from Indonesia. *Virol J* 2012; **9**: 240.

# Appendix Text S7: References searched out in CNKI for the distribution of bat reservoirs (in Chinese)

1. Shao YG, Liu ZX, Liu Q, Chen Y, Zhang LB. Effect of food size on feeding behavior of fruit bats *Rousettus leschenaulti* and *Cynopterus sphinx*. *Acta Theriol Sin* 2014; **34**: 232-7.
2. Zhou YW, Deng LG, Wang GD, Zhang JR, Li YC, Chen Z. Comparison of the gonadal development of the male *Rousettus leschenaultii* and *Miniopterus magnate* in Hainan island. *J Hainan Normal Univ (Natural Science)* 2014; **27**: 171-4.
3. Shao YG. Size-based Food Selection Pattern of the fulvous fruit bat (*Rousettus leschenaulti*) and short-nosed fruit bat (*Cynopterus sphinx*) and their foraging behavioral response to predators' odor [M]: Jishou University; 2014.
4. Hu YM. The study on feeding behavior of two fruit bats, *Rusettus leschenaulti* and *Cynopterus sphinx*, in Xishuangbanna area, southwest China [M]: Northeast Normal University; 2011.
5. Hu YM, Tang ZH, Cao M, Ma XF. The feeding behavior and food intake of *Rousettus leschenaulti* on Dimocarpus longan. *Chinese J Zool* 2010; **45**: 47-53.
6. Shao WW, Hua PY, Zhou SY, Chen JP. The isolation of new microsatellite loci in *Rousettus leschenaulti* and their applicability in closely related species. *Acta Theriol Sin* 2007; (04): 385-388.
7. Chen Z, Meng YH, Zhou F, Li YC. Activity rhythms and food habits of Leschenault's rousette (*Rousettus leschenaulti*) in Hainan Island. *Acta Theriol Sin* 2007; (02): 112-119.
8. Tang ZH, Sheng LX, Ma XF, Cao M, Zhang SY. Fruit consumption and seed dispersal of Morus macroura by two frugivorous bats in Xishuangbanna, Southwest China. *Acta Ecol Sin* 2007; (05): 1895-1902.
9. Zhu GJ. Diet, Roost type of Greater Short-nosed fruit bat (*Cynopterus sphinx*) and activity of fulvous fruit bat (*Rousettus leschenaultii*) in winter in Hainan island [M]. Hainan Normal University; 2007.
10. Peng L. Bats survey in Guangdong and Macau with diet composition of *Cynopterus Sphinx* and the analysis of sugar types in its foraging fruits [M]: Jishou University; 2019.
11. Lin S, Wang LJ. Effect of expressway reconstruction project on local animals and the assessment. *J Anhui Agr Sci* 2012; **40**: 293-298.
12. Tang ZH. Frugivory and seed dispersal by two species of fruit bats [D]. Northeast Normal University; 2007.
13. Hu KL. Study of brain structures and functions of bats using magnetic resonance technique [M]. Guizhou Normal University; 2007.
14. Zhang J. The investigate of the *Bartonella* spp. infection in bats and its ectoparasite from the southwestern Yunnan province [M]. Dali University; 2018.
15. Meng XF. Research on population genetics and phenotypic differentiation of *Hipposideros larvatus* [D]. Northeast Normal University; 2021.
16. Chi TT. Research on vocal development and vocal learning of pups in *Hipposideros larvatus* [M]. Northeast Normal University; 2019.
17. Feng L, Wu QG, Shi SC, et al. Morphostructure and phylogenesis of Horsfield's leaf-nosed bat (*Hipposideros larvatus*) from Hunan province. *Life Sci Res* 2017; **21**: 515-518+522.
18. Zhao LZ, Bu YZ, He XP, Zou HX, Yu Y, Niu HX. Morphological and cyt b sequence divergence of *Hipposideros larvatus* from Hainan and Guangxi provinces, China. *Acta Theriol Sin* 2014; **34**: 278-85.
19. Yang TY, Hou XF, Wang YX, Zhou J. Bat species diversity and conservation in Libo World Natural Heritage Site of South China Karst. *Biodivers Sci* 2014; **22**: 385-91.
20. Wei L, Gan YM, Li ZQ, Lin ZH, Hong TY, Zhang LB. Comparisons of echolocation calls and wing morphology among six sympatric bat species. *Acta Theriol Sin* 2011; **31**: 155-63.
21. Liu YY. Complete mitochondrial genomes and phylogeny of three Hipposideridae bats. [M]. Henan Normal University; 2019.

# Appendix Text S8: References searched out in PubMed for the occurrence of bat-borne viruses

1. Páez DJ, Giles J, McCallum H, et al. Conditions affecting the timing and magnitude of Hendra virus shedding across pteropodid bat populations in Australia. *Epidemiol Infect* 2017; **145**: 3143-53.
2. Holz PH, Lumsden LF, Druce J, et al. Virus survey in populations of two subspecies of bent-winged bats (*Miniopterus orianae bassanii* and *oceanensis*) in south-eastern Australia reveals a high prevalence of diverse herpesviruses. *PLoS One* 2018; **13**: e0197625.
3. Van Brussel K, Mahar JE, Ortiz-Baez AS, et al. Faecal virome of the Australian grey-headed flying fox from urban/suburban environments contains novel coronaviruses, retroviruses and sapoviruses. *Virology*. 2022; **576**: 42-51.
4. Cappelle J, Furey N, Hoem T, et al. Longitudinal monitoring in Cambodia suggests higher circulation of alpha and betacoronaviruses in juvenile and immature bats of three species. *Sci Rep* 2021; **11**: 24145.
5. Razafindratsimandresy R, Jeanmaire EM, Counor D, Vasconcelos PF, Sall AA, Reynes JM. Partial molecular characterization of alphaherpesviruses isolated from tropical bats. *J Gen Virol* 2009; **90**: 44-7.
6. Peng C, Zhang D, Li C, et al. *Rhinolophus sinicus* virome revealed multiple novel mosquito-borne zoonotic viruses. *Front Cell Infect Microbiol* 2022; **12**: 960507.
7. Armero A, Li R, Bienes KM, et al. *Myotis fimbriatus* virome, a window to virus diversity and evolution in the genus Myotis. *Viruses*. 2022; **14**: 1899.
8. Tan Z, Gonzalez G, Sheng J, et al. Extensive genetic diversity of polyomaviruses in sympatric bat communities: host switching versus coevolution. *J Virol*. 2020; **94**: e02101-19.
9. Zhao K, Zhang W, Li B, et al. Ecological study of cave nectar bats reveals low risk of direct transmission of bat viruses to humans. *Zool Res*. 2022; **43**: 514-522.
10. Yang XL, Tan CW, Anderson DE, et al. Characterization of a filovirus (Měnglà virus) from Rousettus bats in China. *Nat Microbiol*. 2019; **4**: 390-395.
11. Yuan L, Li M, Li L, et al. Evidence for retrovirus and paramyxovirus infection of multiple bat species in China. *Viruses*. 2014; **6**: 2138-2154.
12. Feng Y, Ren X, Xu Z, et al. Genetic diversity of the Yokose virus, XYBX1332, isolated from bats (*Myotis daubentonii*) in China. *Virol J*. 2019; **16**: 8.
13. Chen YN, Phuong VN, Chen HC, Chou CH, Cheng HC, Wu CH. Detection of the severe acute respiratory syndrome-related coronavirus and alphacoronavirus in the bat population of Taiwan. *Zoonoses Public Health*. 2016; **63**: 608-615.
14. Zheng XY, Qiu M, Guan WJ, et al. Viral metagenomics of six bat species in close contact with humans in southern China. *Arch Virol*. 2018; **163**: 73-88.
15. Zhou H, Ji J, Chen X, et al. Identification of novel bat coronaviruses sheds light on the evolutionary origins of SARS-CoV-2 and related viruses. *Cell*. 2021; **184**: 4380-4391.e14.
16. Liu Y, Zhang S, Zhao J, Zhang F, Hu R. Isolation of Irkut virus from a *Murina leucogaster* bat in China. *PLoS Negl Trop Dis*. 2013; **7**: e2097.
17. Du L, Lu Z, Fan Y, et al. Xi River virus, a new bat reovirus isolated in southern China. *Arch Virol*. 2010; **155**: 1295-1299.
18. Mourya DT, Yadav PD, Basu A, et al. Malsoor virus, a novel bat phlebovirus, is closely related to severe fever with thrombocytopenia syndrome virus and heartland virus. *J Virol*. 2014; **88**: 3605-3609.
19. Dhanze H, Karikalan M, Mehta D, et al. First report on the detection of Japanese encephalitis virus in fruit bats from India. *J Vector Borne Dis*. 2022; **59**: 190-192.
20. Shete AM, Yadav P, Kumar V, et al. Development of polymerase chain reaction-based diagnostic tests for detection of Malsoor virus & adenovirus isolated from Rousettus species of bats in Maharashtra, India. *Indian J Med Res*. 2017; **145**: 90-96.
21. Yadav P, Sarkale P, Patil D, et al. Isolation of Tioman virus from *Pteropus giganteus* bat in North-East region of India. *Infect Genet Evol*. 2016; **45**: 224-229.
22. Lau SK, Woo PC, Li KS, et al. Severe acute respiratory syndrome coronavirus-like virus in Chinese horseshoe bats. *Proc Natl Acad Sci U S A*. 2005; **102**: 14040-14045.
23. Ain-Najwa MY, Yasmin AR, Arshad SS, et al. Exposure to Zoonotic West Nile Virus in Long-Tailed Macaques and Bats in Peninsular Malaysia. *Animals (Basel)*. 2020; **10**: 2367.
24. Tan CS, Noni V, Sathiya Seelan JS, Denel A, Anwarali Khan FA. Ecological surveillance of bat coronaviruses in Sarawak, Malaysian Borneo. *BMC Res Notes*. 2021; **14**: 461.
25. Zana B, Kemenesi G, Buzás D, et al. Molecular Identification of a Novel Hantavirus in Malaysian Bronze Tube-Nosed Bats (*Murina aenea*). *Viruses*. 2019; **11**: 887.
26. Arai S, Kikuchi F, Bawm S, et al. Molecular Phylogeny of Mobatviruses (Hantaviridae) in Myanmar and Vietnam. *Viruses*. 2019; **11**: 228.
27. Taniguchi S, Maeda K, Horimoto T, et al. First isolation and characterization of pteropine orthoreoviruses in fruit bats in the Philippines. *Arch Virol*. 2017; **162**: 1529-1539.
28. Arai S, Taniguchi S, Aoki K, et al. Molecular phylogeny of a genetically divergent hantavirus harbored by the Geoffroy's rousette (*Rousettus amplexicaudatus*), a frugivorous bat species in the Philippines. *Infect Genet Evol*. 2016; **45**: 26-32.
29. Jayme SI, Field HE, de Jong C, et al. Molecular evidence of Ebola Reston virus infection in Philippine bats. *Virol J*. 2015; **12**: 107.
30. Gunawardena PS, Marston DA, Ellis RJ, et al. Lyssavirus in Indian flying foxes, Sri Lanka. *Emerg Infect Dis*. 2016; **22**: 1456-1459.
31. Latinne A, Nga NTT, Long NV, et al. One health surveillance highlights circulation of viruses with zoonotic potential in bats, pigs, and humans in Viet Nam. *Viruses*. 2023; **15**: 790.
32. Arai S, Aoki K, Sơn NT, et al. Đakrông virus, a novel mobatvirus (Hantaviridae) harbored by the Stoliczka's Asian trident bat (*Aselliscus stoliczkanus*) in Vietnam. *Sci Rep*. 2019; **9**: 10239.
33. Arai S, Nguyen ST, Boldgiv B, et al. Novel bat-borne hantavirus, Vietnam. *Emerg Infect Dis*. 2013; **19**: 1159-1161.
34. Inagaki T, Yamada S, Fujii H, et al. Characterization of a novel alphaherpesvirus isolated from the fruit bat *Pteropus lylei* in Vietnam. *J Virol*. 2020; **94**: e00673-20.
35. Iglesias R, Cox-Witton K, Field H, Skerratt LF, Barrett J. Australian bat lyssavirus: analysis of national bat surveillance data from 2010 to 2016. *Viruses*. 2021; **13**: 189.
36. Prada D, Boyd V, Baker ML, O'Dea M, Jackson B. Viral diversity of microbats within the South West Botanical Province of Western Australia. *Viruses*. 2019; **11**: 1157.
37. O'Connor TW, Finlaison DS, Kirkland PD. What can we learn from over a decade of testing bats in New South Wales to exclude infection with Australian bat lyssaviruses? *Aust Vet J*. 2022; **100**: 172-180.
38. Lau SK, Li KS, Huang Y, et al. Ecoepidemiology and complete genome comparison of different strains of severe acute respiratory syndrome-related Rhinolophus bat coronavirus in China reveal bats as a reservoir for acute, self-limiting infection that allows recombination events. *J Virol*. 2010; **84**: 2808-2819.
